# District level inequality in reproductive, maternal, neonatal and child health coverage in India

**DOI:** 10.1186/s12889-020-8151-9

**Published:** 2020-01-14

**Authors:** Basant Kumar Panda, Gulshan Kumar, Ashish Awasthi

**Affiliations:** 1 International Institute for Population Sciences, Mumbai, Maharashtra India; 2 Centre for Chronic Conditions and Injuries, Public Health Foundation of India, Gurugram, India

**Keywords:** Coverage gap index, Districts, India, RMNCH, Spatial, Inequality

## Abstract

**Background:**

As India already missed maternal and child health related millennium development goals, the maternal and child health outcomes are a matter of concern to achieve sustainable development goals (SDGs). This study is focused to assess the gap in coverage and inequality of various reproductive, maternal, neonatal and child health (RMNCH) indicators in 640 districts of India, using data from most recent round of National Family Health Survey.

**Methods:**

A composite index named Coverage Gap Index (CGI) was calculated, as the weighted average of eight preventive maternal and child care interventions at different administrative levels. Bivariate and spatial analysis were used to understand the geographical diversity and spatial clustering in districts of India. A socio-economic development index (SDI) was also derived and used to assess the interlinkages between CGI and development. The ratio method was used to assess the socio-economic inequality in CGI and its component at the national level.

**Results:**

The average national CGI was 26.23% with the lowest in Kerala (10.48%) and highest in Nagaland (55.07%). Almost half of the Indian districts had CGI above the national average and mainly concentrated in high focus states and north-eastern part. From the geospatial analysis of CGI, 122 districts formed hotspots and 164 districts were in cold spot. The poorest households had 2.5 times higher CGI in comparison to the richest households and rural households have 1.5 times higher CGI as compared to urban households.

**Conclusion:**

Evidence from the study suggests that many districts in India are lagging in terms of CGI and prioritize to achieve the desired level of maternal and child health outcomes. Efforts are needed to reduce the CGI among the poorest and rural resident which may curtail the inequality.

## Background

Despite the continued global effort to improve maternal and child health in last 25 years through Millennium Development Goals (MDGs) and Sustainable Development Goals (SDGs), the progress on utilization of maternal and child health services (MCH) and reduction of maternal and child mortality is not equally distributed among and within countries [1, 2]. The fourth goal of MDGs (MDG-4) was set to reduce two-third of under-five mortality rate, whereas the fifth goal (MDG-5) had focused on improving maternal health by cutting 75% of maternal mortality between 1990 and 2015. Out of 75 countries included in the countdown study, only one-third of countries achieved the targets of MDG-4, and only six countries were able to accomplish the MDG-5 [3]. Globally, about 0.3 million mothers died during childbirth or pregnancy-related complexities and about 2.6 million neonatal deaths occurred in 2015 and most of them had taken place in the developing countries [1, 4]. Irrespective of other concerned factors, high impact intervention and health system strengthening remains a prime component to reduce maternal and child mortality, mainly in low and middle income countries (LMICs) [5]. At the same time, the key set of the interventions on maternal and child health remained unacceptably low in many LMICs including India [6–8].

In the last two decades, a substantial improvement in reducing maternal and neonatal mortality was observed in India, with accelerated progress of essential health interventions [9]. Despite this, the country failed to meet MDG targets of maternal and child mortality. The country ranked 143 among 188 countries in the SDG progress indicators, with widespread heterogeneity across the regional level [10, 11]. At the same time, inequality in many maternal and child health indicators is widening over time [12, 13]. Moreover, the disadvantaged population subgroups (i.e., the poorest, the least educated and those residing in rural areas) had lower health care access and coverage, and worse health outcomes [14, 15].

The 2015 countdown analysis, replaced by the countdown 2030, annually monitors the progress in the coverage indicators of the countdown countries at the national level [6, 8, 16]. Though there has been substantial progress achieved by many countries, the achievement was uneven and need more significant acceleration [6, 17–21]. Concerns about health inequity indicate that there is a need for analysis of health indicators at the microgeographic level or the population subgroups. Few studies explored the pattern of reproductive, maternal, neonatal and child health (RMNCH) coverage by focusing equity in utilization in India [7, 22–24]. Some studies also tried to explain the coverage gap in RMNCH services mainly in the high focused states and its association with health outcomes [22, 25]. However, no study was focused on the coverage of essential health services in all the districts of India. Thus, it is vital to assess the coverage gap to help in the precise distribution of resources and effective health programming.

With this background, the study primarily has two broad objectives. First, the study estimates the variation in the coverage gap for essential maternal and child health services through the Coverage Gap Index (CGI) across the districts of India. It further explores the spatial implications of maternal and child health coverage at the sub-national levels and facilitates the identification of pitfall districts. Secondly, the study assesses the inequality in the coverage of these indicators among the wealth quintile and the place of residence.

## Methods

### Data

We used the data from the fourth round of the National Family Health Survey (NFHS-4), the most recent large-scale multistage survey conducted in India in the year 2015–16. The survey provides comprehensive estimates of various maternal and child health indicators on the national and regional level. The study used the multistage stratified random sampling method to select the household. A total of 601,509 households, 699,686 number of women and 112,122 number of men, were interviewed in the survey. Information on 259,627 children born in the last 5 years prior to the survey was collected from their mothers. Details about the sample size, design and sample weights were provided in the final report of NFHS-4 [9].

## Coverage gap index: context, dimensions and indicators

The outcome variable of this study was the Coverage Gap Index (CGI) which is the difference of the Composite Coverage Index (CCI) from universal achievement. CCI was first proposed in 2008 as the weighted average of eight preventive and curative interventions received along the continuum of maternal and child care [19]. The index was calculated at the group level, either for a whole country or by subgroups such as wealth quintiles or geographical regions. The selected indicators are categorized into four groups: first- reproductive services (family planning coverage), second- maternal and newborn care (antenatal care and skilled birth attendant), third- immunization (BCG; three doses of diphtheria, pertussis, and tetanus (DPT3); and measles vaccines) and forth- management of illness (oral rehydration therapy and care-seeking for pneumonia). All the four domains are equally weighted, and within each domain, all indicators have the same weight, except for DPT3, which has a higher weight because three doses are needed. Detail definition of the indicators is presented in Table 1.

**Table 1:** Definition of the selected indicators used in the study

Indicators	Definition
Need for family planning satisfied (FP)	Percentage of currently married women who say that they do not want any more children or that they want to wait 2 or more years before having another child, and are using contraception
Indicators for maternal and newborn care	
Skilled birth attendant (SBA)	Percentage of live births in the five years before the survey attended by skilled health personnel (doctor, nurse, midwife or auxiliary midwife)
Antenatal Care coverage (ANC)	Percentage of women who were attended to at least once during pregnancy by skilled health personnel for reasons related to the pregnancy in the five years before the survey
Indicator of Immunization	
Measles vaccination (MSL)	Percentage of children aged 12–23 months who are immunised against measles
Diphtheria, pertussis and tetanus vaccination (DPT3)	Percentage of children aged 12–23 months who received three doses of diphtheria, pertussis and tetanus
BCG vaccination (BCG)	Percentage of children aged 12–23 months currently vaccinated against BCG
Indicators for the treatment of sick children	
Oral rehydration therapy (ORT)	Percentage of children under five year with diarrhoea in the past two weeks who received oral rehydration therapy (packets of oral rehydration salts, recommended home solution or increased fluids) and continued feeding
Treatment of acute respiratory infection (PNCM)	Percentage of children aged 0–59 months with suspected pneumonia (cough and dyspnoea) who sought care from a health provider

Source: Boerma et al. 2008 [19]; Countdown 2008 equity analysis group

Based on these indicators explained above the CGI is calculated at the district level. The formula for finding the CGI is given below.

$$ CGI=100-\left(\frac{1}{4}\left( FP+\frac{ORT+ ARI}{2}+\frac{SBA+ ANC}{2}+\frac{MSL+2 DPT3+ BCG}{4}\right)\right) $$

*Abbreviations of all the above Indicators are provided in Table 1 and Abbreviation section.

### Socio-demographic development index

A socio-economic demographic development index (SDI) was constructed to understand the development pattern in the districts of India. The variables selected for the construction of SDI were based on existing literature. These indicators includes the asset index, coverage of safe drinking water, sanitation, and electricity, the percentage of female literate and level of urbanization [22]. The asset index was based on 20 assets collected at the household level and estimated for each districts of India. The indicators were calculated and standardized using the formula used to calculate the Human Development Index (HDI) at districts level. Geometric mean was used to estimate the SDI as it reduces the substitutability between the various dimensions.

### Spatial analysis

The spatial distribution map was used to understand the spatial pattern of the CGI in the districts of India. It gives a bird’s eye view of spatial inequality in maternal and child health coverage at the sub-regional level (states and districts). Two techniques had been used for this analysis. First is a simple visual representation and the other being spatial autocorrelation. The first technique used to provide the distribution of any indicators across the districts of India. The second technique used to elucidate the degree to which one area is similar to or different from its neighboring area [26].

Global Moran’s I statistic was used to understand whether there was a spatial autocorrelation among the regional distribution. The Moran’s I value ranges from − 1 to + 1. Positive value signifies spatial clustering, and negative values indicate no clustering and zero indicate no or random spatial clustering [27, 28].

Local indicator of spatial association (LISA) measure of local Moran’s I, which was used to identify the presence or absence of significant spatial clusters or outliers for each geographical unit. We used the GeoDa 1.14.0 software package for this analysis with 999 permutations and a pseudo-*p*-value for a cluster of < 0.05 specified.

### Inequality

To understand the inequality among the various population subgroup, this study used absolute as well as a relative measure of inequality. For wealth-related inequality, the difference between the fifth (Q5) and first wealth quintile (Q1) was calculated. The ratio between both the fifth and first quintiles was also calculated to understand the relative inequality. Similarly, for the place of residence, the differential in the rural and urban areas and rural-urban inequality ratio was calculated. Stata 15.1 was used for statistical analysis and a *p*-value < 0.05 was considered significant for a two tailed alternate hypothesis.

## Results

**Fig. 1:**
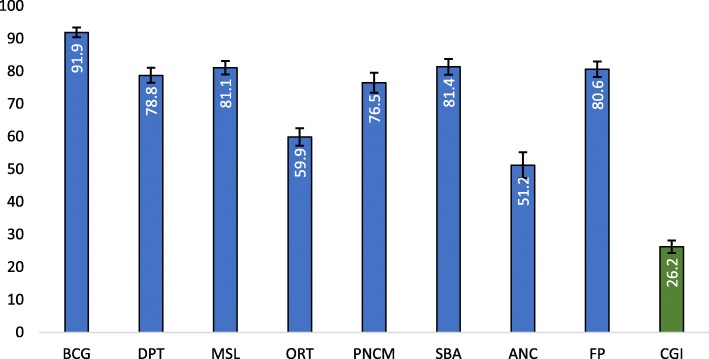
Percentage coverage of CGI and essential interventions in India. Source: Authors generated the figure

**Fig. 2:**
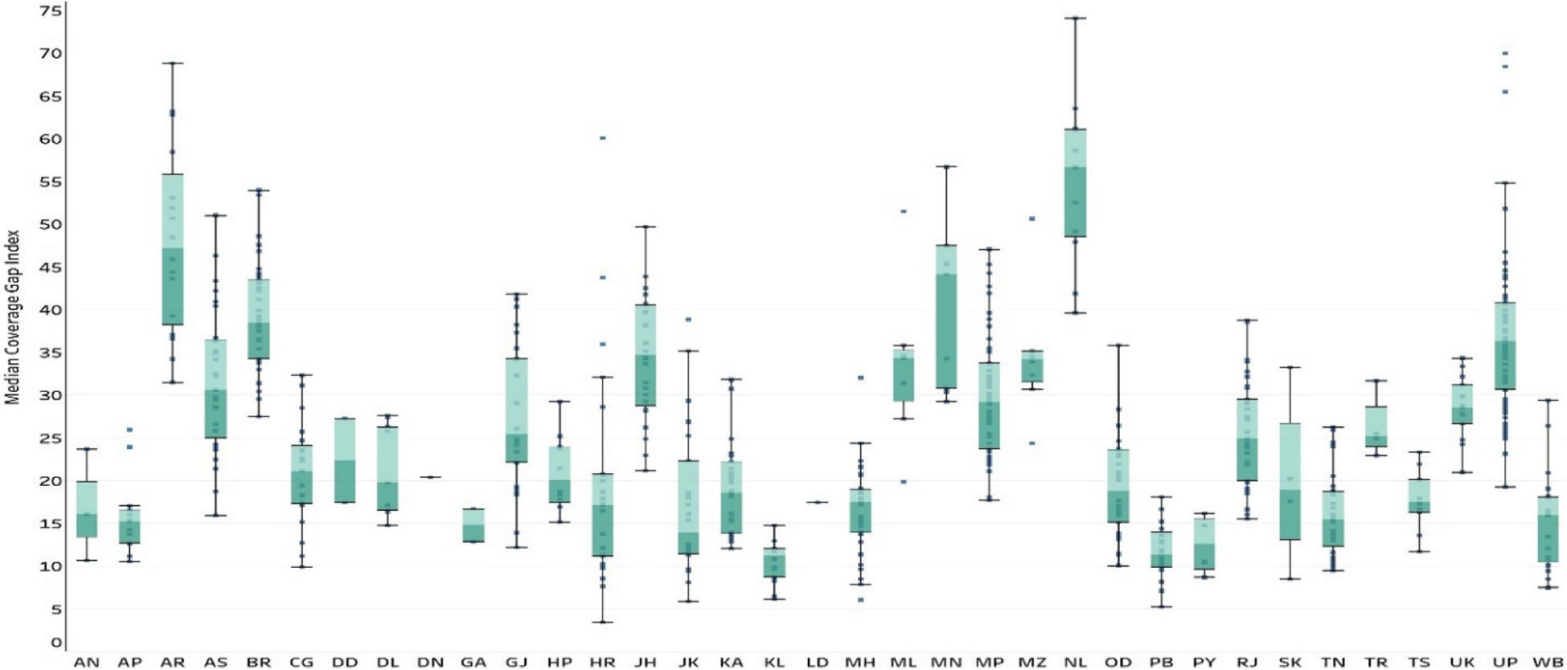
Coverage Gap Index (CGI) of India and its states in 2015–16. Source: Authors generated the figure. * AN: Andaman and Nicobar Islands, AP: Andhra Pradesh, AR: Arunachal Pradesh, AS: Assam, BR: Bihar, CG: Chhattisgarh, DN: Dadra and Nagar Haveli, DD: Daman and Diu, DL: Delhi, GA: Goa, GJ: Gujarat, HR: Haryana, HP: Himachal Pradesh, JK: Jammu and Kashmir, JH: Jharkhand, KA: Karnataka, KL: Kerala, LD: Lakshadweep, MP: Madhya Pradesh, MH: Maharashtra, MN: Manipur, ML: Meghalaya, MZ: Mizoram, NL: Nagaland, OD: Odisha, PY: Puducherry, PB: Punjab, RJ: Rajasthan, SK: Sikkim, TN: Tamil Nadu, TS: Telangana, TR: Tripura, UP: Uttar Pradesh, UK: Uttarakhand, WB: West Bengal

Figure 1 presents the pattern of CGI and its components in India. The CGI in India found to be 26.23% (95% CI: 25.27,27.20), which varies across the indicator. Among the various RMNCH indicators, the highest coverage had been found for BCG (91.9%) followed by SBA (81.4%) and least coverage for the ANC (51.2%). While presenting the CGI across the states and India, it showed wide variation (See Fig. 2). Lower estimates of CGI was found for Kerala followed by Punjab and Tamil Nadu. Similarly, higher CGI was observed for the Nagaland followed by Arunachal Pradesh and Bihar. Fourteen states, residence of 49% of the Indian population, had higher CGI as compared to the national estimate. These states include the high focused states (EAG states) and the north-eastern states. Among the high focused sates Odisha had the lowest CGI followed by Chhattisgarh whereas, Bihar has the highest CGI followed by Uttar Pradesh.

**Fig. 3:**
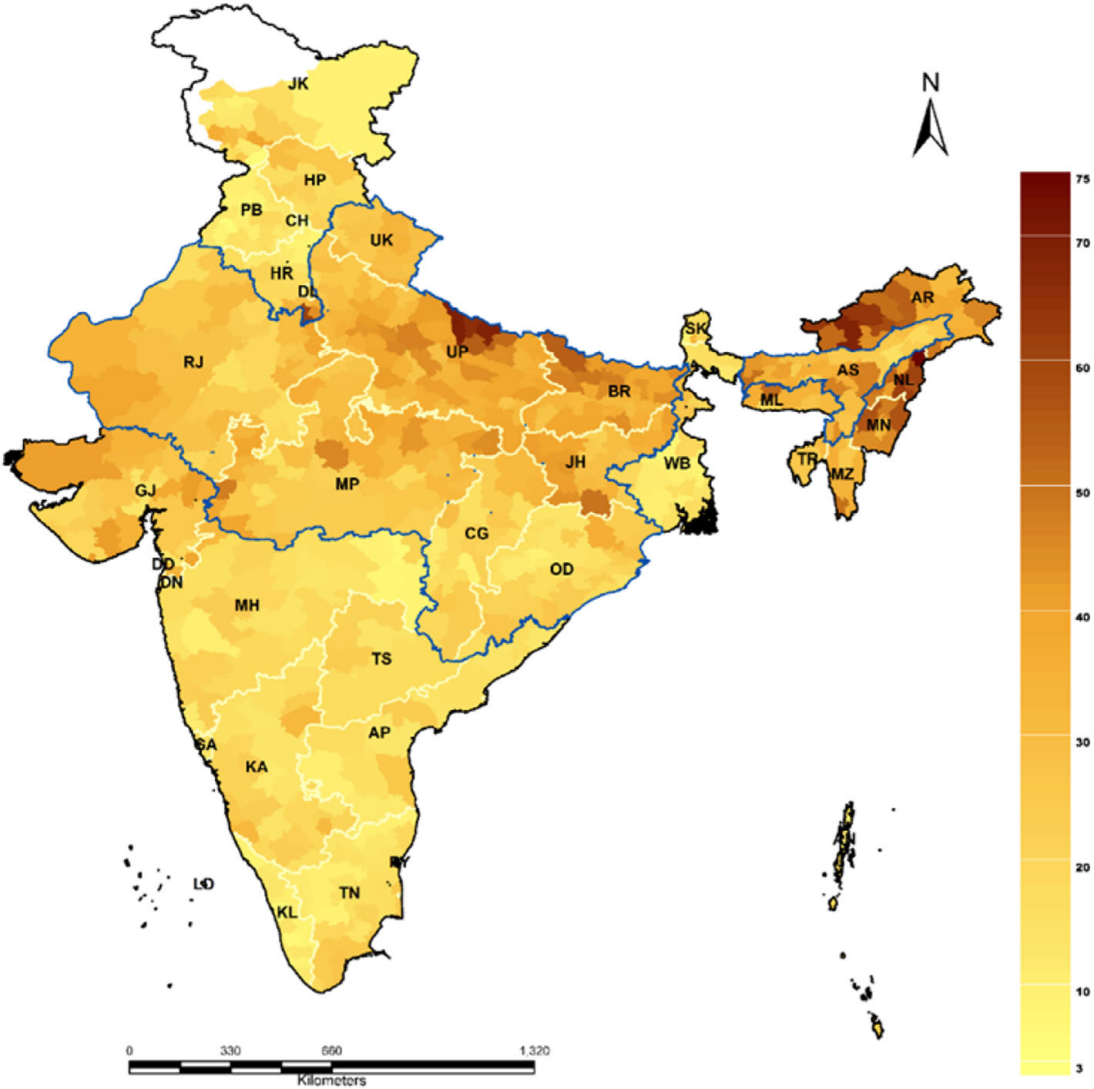
Coverage Gap Index (CGI) in districts of India in 2015–16. Source: Authors generated the map using Arc GIS 10.1

The district specific CGI is presented in Fig. 3. The detailed estimate of CGI and the source indicators are presented in Additional file 1. In India, 308 districts had higher CGI as compared to the national average. Thiry-one districts had CGI less than 10%, while 124 districts ranging between 35% - 50% and 27 districts had CGI more than 50%. Among all the districts, Mon district of Nagaland had the highest CGI (74.1%) followed by Bahraich district (69.9%) of Uttar Pradesh. The top and bottom 20 districts of each indicator as well as CGI were presented in Additional file 2. Among the top 20 districts in terms of CGI, 6 districts were from Nagaland, 5 from Arunachal Pradesh, 4 from Uttar Pradesh, 2 each from Bihar and Manipur and 1 from Haryana.

**Fig. 4:**
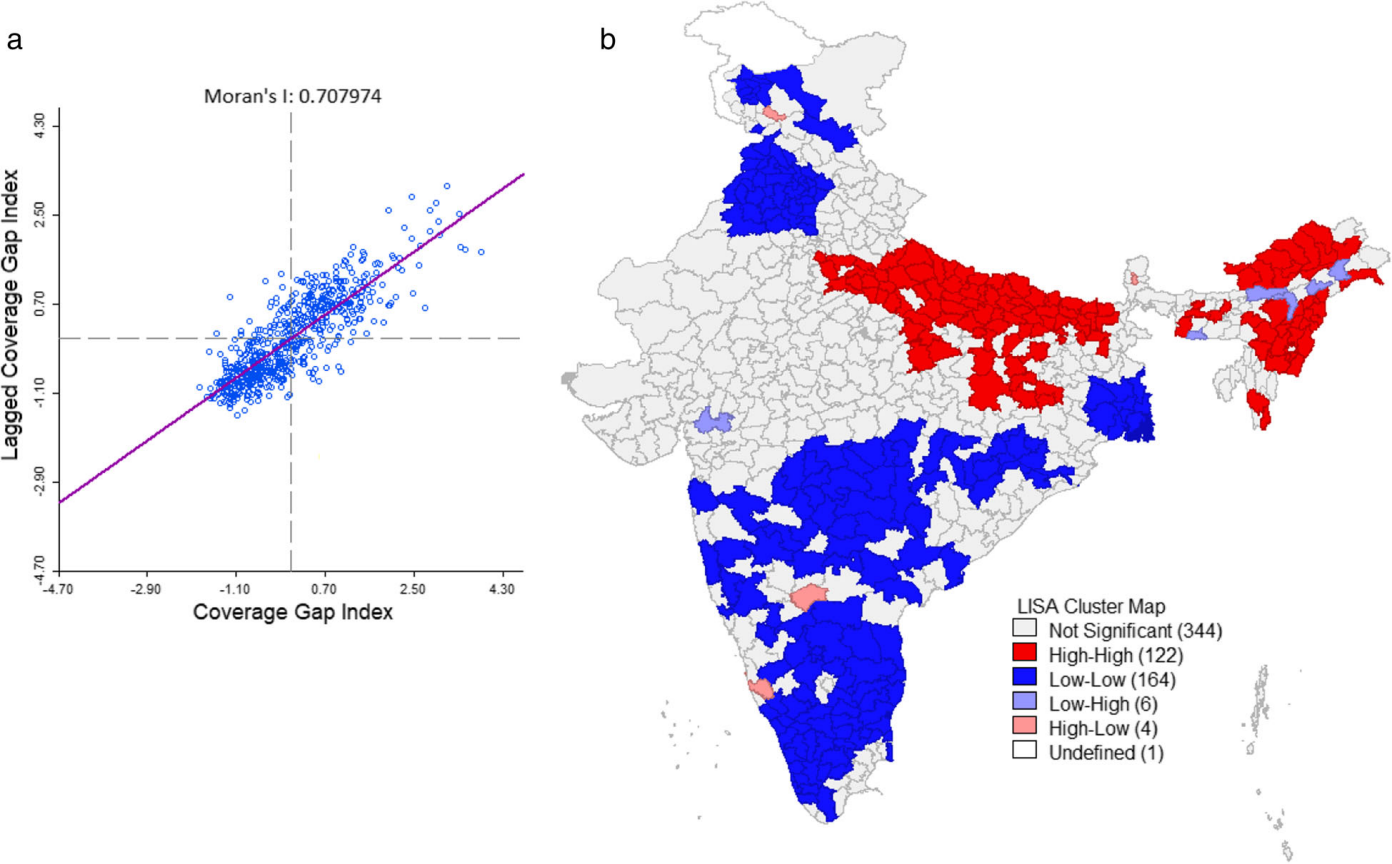
Global Moran’s I and univariate LISA cluster map of CGI in the districts of India 2015–16. **a** Moran’s I scatter plot, **b** Univariate LISA cluster map. Source: Authors generated the map using GeoDa version 1.14.0

Figure 4a presents the Global Moran’s I value of CGI for districts of India. The Global Moran’s I value was 0.70 (*p* < 0.01, 999 permutations), which signifies that there was a high spatial autocorrelation and a significant positive association of CGI among districts of India. Figure 4b presents a univariate LISA cluster map of CGI at the districts of India. Out of 640 districts 122 districts were found as hot spots, which symbolize that these districts had higher CGI with their neighboring districts. The hotspots districts were clustered in the state of, Uttar Pradesh, Madhya Pradesh, Assam, Bihar, Arunachal Pradesh, Nagaland, and Manipur.

### Development pattern and its linkage with the RMNCH in districts of India

**Fig. 5:**
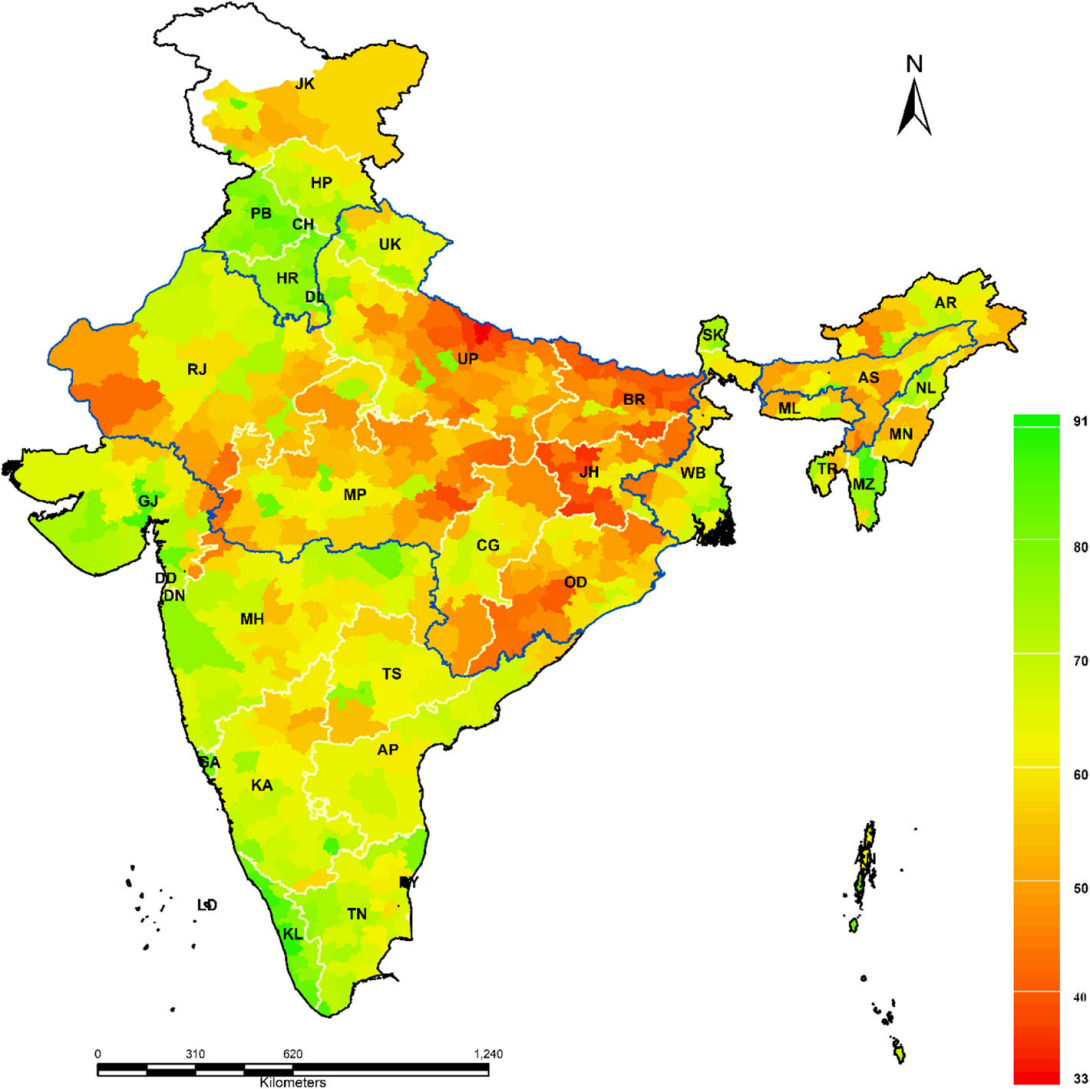
Level of development in districts of India 2015–16. Source: Authors generated the map using Arc GIS 10.1. * AN: Andaman and Nicobar Islands, AP: Andhra Pradesh, AR: Arunachal Pradesh, AS: Assam, BR: Bihar, CH: Chandigarh, CG: Chhattisgarh, DN: Dadra and Nagar Haveli, DD: Daman and Diu, DL: Delhi, GA: Goa, GJ: Gujarat, HR: Haryana, HP: Himachal Pradesh, JK: Jammu and Kashmir, JH: Jharkhand, KA: Karnataka, KL: Kerala, LD: Lakshadweep, MP: Madhya Pradesh, MH: Maharashtra, MN: Manipur, ML: Meghalaya, MZ: Mizoram, NL: Nagaland, OD: Odisha, PY: Puducherry, PB: Punjab, RJ: Rajasthan, SK: Sikkim, TN: Tamil Nadu, TS: Telangana, TR: Tripura, UP: Uttar Pradesh, UK: Uttarakhand, WB: West Bengal

The level of development in districts of India from sociodemographic angle was shown in Fig. 5. The map portrayed the clear picture of inter-districts and intra-state disparity. Districts of Kerala, Goa, western Maharashtra, Gujarat, Punjab, and Haryana had a high level of development (More than 70%) whereas districts of Bihar, Uttar Pradesh, Madhya Pradesh Rajasthan, north-eastern states had a low level of development (Below 40%). Detailed data was given in Additional files 3 and 4.

**Fig. 6:**
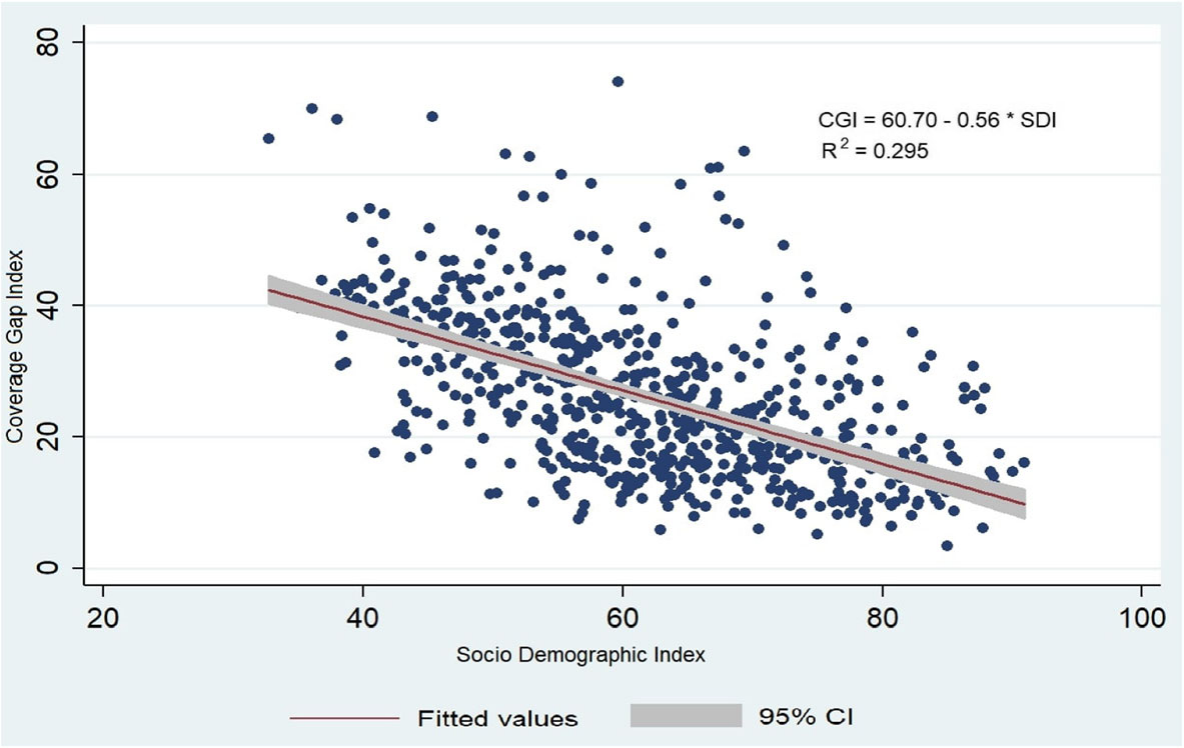
Scatter plot of CGI and SDI by district in India. Source: Authors generated the figure

Figure 6 presents the relationship between the SDI and CGI in the districts of India. The correlation coefficients among the two was − 0.54 (*P* < 0.001), which signifies the higher the development lower is the CGI. The relationship between CGI and SDI was written as

$$ \mathrm{CGI}=60.70-0.56\ast \mathrm{SDI} $$

**Fig. 7:**
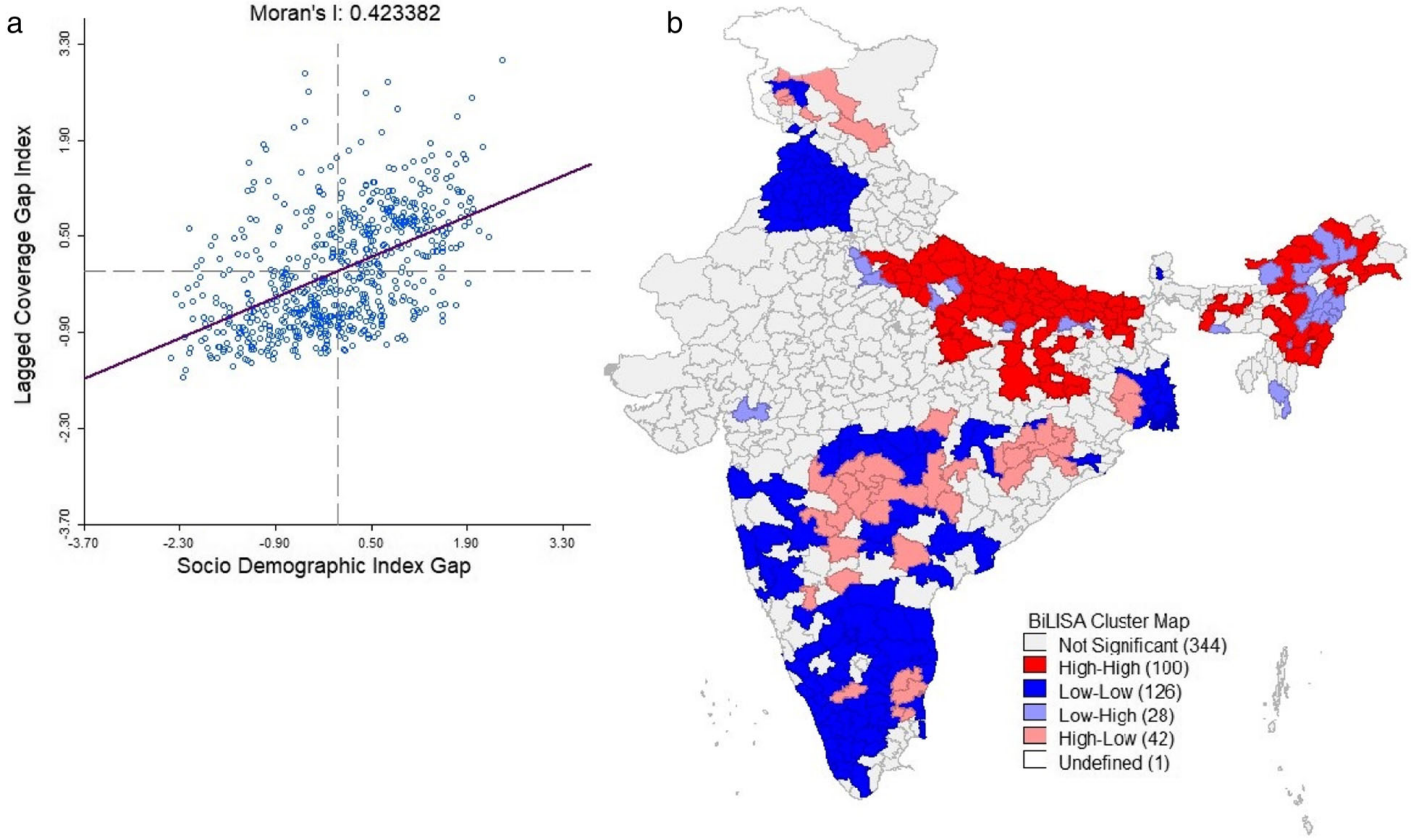
Global Moran’s I and Bivariate LISA map of CGI and SDI gap in districts of India. **a** Moran’s I scatter plot, **b** Bivariate LISA cluster map. Source: Authors generated the map using GeoDa version 1.14.0

Bivariate LISA analysis was performed to understand the relationship between the development and the CGI in the districts of India. This analysis gives compelling evidence to understand the geographical regions which were deprived in the development were also underprivileged in the composite coverage. The global Moran I index was found to be 0.42 (*p* < 0.01, 999 permutation) which signifies the moderate autocorrelation but positive association among the CGI and development status in the districts of India (Fig. 7a). It was observed from Fig. 7b. The bivariate LISA analysis map of CGI versus SDI gap that 100 districts had high-high clusters (Hotspots) and 126 districts had low-low clusters (cold spots).

Table 2 presents the coverage of the selected indicators in different wealth quintile in India. Both absolute and relative inequality was used to understand the pattern of inequality in the selected indicators. The CGI was 2.3 times higher among the poorest as compared to richest. Richest to the poorest gap and the ratio among richest and poorest wealth quintile was highest for the ANC followed by SBA and lowest for the BCG vaccines. Table 3 summarises the CGI and inequality among rural and urban residence in India. The CGI of the urban area was 0.72 times lower as compared to rural areas. The rural-urban CGI difference was high for ANC followed by SBA and ORT.

**Table 2:** Percentage coverage of essential interventions in India and by wealth quintile in NFHS-4, 2015–16

Indicators	Coverage	Poorest (Q_1_)	Poorer	Middle	Richer	Richest (Q_5_)	Difference (Q_5_-Q_1_)	Richest/Poorest Ratio
BCG	91.90 (0.12)	86.99 (0.30)	91.24 (0.27)	93.21 (0.25)	94.84 (0.24)	95.42 (0.25)	8.43	1.10
DPT	78.75 (0.18)	70.40 (0.41)	77.06 0.39)	80.88 (0.39)	83.82 (0.40)	85.66 (0.42)	15.26	1.22
MSL	81.10 (0.17)	73.18 (0.40)	78.93 (0.38)	83.07 (0.37)	85.71 (0.38)	88.79 (0.38)	15.61	1.21
ORT	59.87 (0.33)	53.22 (0.64)	56.93 (0.68)	60.26 (0.73)	65.98 (0.78)	70.71 (0.86)	17.49	1.33
PNCM	76.46 (0.36)	65.91 (0.81)	74.72 (0.75)	79.49 (0.77)	83.52 (0.78)	86.66 (0.82)	17.37	1.25
SBA	81.36 (0.08)	64.15 (0.18)	78.26 (0.17)	86.83 (0.15)	91.76 (0.13)	95.45 (0.11)	31.30	1.49
ANC	51.22 (0.11)	24.99 (0.20)	44.36 (0.24)	57.19 (0.25)	65.77 (0.26)	73.05 (0.26)	48.06	2.92
FP	80.64 (0.07)	71.54 (0.18)	79.92 (0.15)	82.94 (0.14)	82.86 (0.15)	83.56 (0.15)	11.38	1.16
CGI	26.23	37.27	27.97	22.67	19.14	16.16	−21.11	0.43

Numbers shown in table are average proportion of coverage and in parenthesis are standard deviation of coverage

**Table 3:** Percentage coverage of essential interventions in India and by rural and urban area in NFHS-4, 2015–16

Indicators	Rural	Urban	Urban-Rural Difference	Urban/Rural Ratio
BCG	91.37 (0.15)	93.21 (0.23)	1.84	1.02
DPT	78.03 (0.36)	80.55 (0.21)	2.52	1.03
MSL	80.26 (0.34)	83.23 (0.21)	2.97	1.04
ORT	57.01 (0.38)	68.24 (0.65)	11.23	1.20
PNCM	74.45 (0.43)	82.31 (0.69)	6.91	1.09
SBA	78.00 (0.09)	89.99 (0.12)	11.99	1.15
ANC	44.81 (0.13)	66.36 (0.22)	21.55	1.48
FP	79.65 (0.08)	82.48 (0.12)	2.64	1.03
CGI	27.82	19.92	−7.90	0.72

Numbers shown in table are average proportion of coverage and in parenthesis are standard deviation of coverage

## Discussion

As every sixth human being belongs to India, it is important that India must achieve SDG targets for global as well as regional accomplishment. The coverage of RMNCH interventions is crucial for the overall improvement of the health outcome in any population [16]. In this regard, several programs have been formulated to improve maternal and child health in India. The Safe Motherhood and Child Health Program (CSSM), Reproductive and Child Health Program (RCH) and the National Health Mission (NHM) aimed to improve the maternal and child health services coverage across all regions and among various population groups. Despite all the efforts of the Government of India in the last two decades, the coverage remains unacceptably low particularly among few specific population subgroups and in specific geographic regions [22–25, 29, 30].

We presented a detailed analysis of the spatial pattern of CGI at the districts of India. To the best of our knowledge, this is the first study to cover all 640 districts of India. This study was successful in identifying inter-district variation in coverage gap of essential reproductive, maternal and child health intervention which have the foremost importance in SDGs. The study found the spatial clustering of MCH services using geospatial analysis, which is essential for the program and policy perspective. The followings are the salient findings of this paper.

One of the important contributions of this paper was the variation of CGI in the state and districts of India. The CGI in India was found to be 26.23% varied largely across the states of India from 10.48% in Kerala to 55.07% in Nagaland. The RMNCH pattern at districts level showed that CGI varied between 3.41% in Panchkula districts of Haryana to 74.09% in Mon districts of Nagaland. The geographical distribution of CGI reveals that many districts of high focused states such as Uttar Pradesh, Bihar, and Rajasthan and all North-Eastern states had unusually high CGI. This is analogous to the previous studies explaining the districts of high focused states having a higher gap in RMNCH coverage [7, 22, 25]. These districts are burdened mainly with the higher maternal and child morbidity and mortality [31]. A few of the reasons which were discussed in literature were, inadequate availability and accessibility of quality health services near the place of residence, availability of doctors and skilled health persons and poor health care awareness remain the top barriers for utilizing the health services in India [32, 33].

The geospatial pattern of inequality in CGI showed that there was a spatial interlinkage CGI among the districts of India. From the LISA cluster map, 122 districts were identified as hotspots which indicates that these districts had a high CGI gap with their neighboring districts. These districts were mainly concentrated in northern, central and north-eastern regions of India. Similarly, 164 districts had found as cold spots that signify these districts had the lower CGI with their neighboring districts. These patterns may guide the policymakers and planners to understand the spatial dimension of the cluster which had relatively poor coverage in maternal and child health services.

The study attempted to understand the interlinkage of the pattern of development and the CGI in the districts of India. The SDI which is a proxy indicator of development showed an inter districts variation. The pattern of SDI remained shortfall in many districts of the Bihar, Uttar Pradesh, Madhya Pradesh, Assam, and other north-eastern states. The study found the negative association of the SDI with the CGI in the districts of India. The bivariate spatial analysis found 100 districts as hotspots which signifies that these districts had higher CGI and the higher gap in SDI with their neighboring districts. This pattern signifies that the importance of socio-demographic variables such as household economic status, maternal education, sanitation and hygiene practice of household had a significant role in the improvement of maternal and child health care utilization.

One of the significant contributions of this study is to understand the pattern and inequality in CGI among the population subgroup. The study found the distinct gap in CGI among the poorest and richest wealth quintile in India. The CGI in the poorest wealth quintile was 2.5 times higher as compared to the richest wealth quintile. Moreover, we found a significant coverage gap among rural and urban areas. The coverage gap in the urban area was 1.5 times higher as compared to the rural area. It highlights the disparity among the worst off and better off. It is analogous to various national and international studies [8, 21, 34].

Our estimates of RMNCH coverage and inequality provides essential policy recommendations. Research at the micro-level is necessary to strengthen and develop health information systems effectively. Besides this, the improvement of literacy especially among females, empowering women, awareness through media and community participation, are equally important in increasing coverage and reducing the disparity and deprivation among the districts [22, 23].

## Strength and limitations

The main strength of this study was to assess the CGI across all the districts of India. Geospatial visualization of CGI across the districts is a novel attempt to identify the lagging districts. As our study is based on secondary data it has all the inherent limitations of cross-sectional studies. Methodologically, CGI is calculated at a group level, which limits us to estimate the causal factors at the individual level. Another limitation was the selection of assets to compute SDI, as the choice and need of the assets vary by urban and rural which may change the classification of households. Despite this limitation, the asset-based index remains the preferred method to explore gaps between rich and poor in low and middle-income countries.

## Conclusion

The overwhelming targets of the SDG require targeted actions towards universal coverage of health services with equity, which can be accompanied by accelerated progress in underprivileged populations. Based on the findings we identified districts that had a higher gap in CGI and need attention. Developmental strategies at the state and districts level are required to be focused on marginalized person of society. The determination to improve RMNCH services in all population subgroup is essential which have an implication in achieving the SDGs. Monitoring these RMNCH indicators is important for government and policymakers in farming policy especially in the regions with higher needs.

## Supplementary information

**Additional file 1:** Percentage distribution of selected variables and asset index by districts of India 2015–16.

**Additional file 2:** Coverage of various RMNCH indicators in percentages, twenty lowest and highest districts across India, NFHS-4, 2015–16.

**Additional file 3:** Percentage coverage of asset, water, sanitation, female literacy, urbanization and the socio-demographic development index (SDI) by districts of India.

**Additional file 4:** Performance of asset index and percentage coverage of associated assets by districts of India in 2015–16.

## Data Availability

The unit-level data is available from the Demographic Health Survey (DHS) data repository through https://dhsprogram.com/data/dataset/India_Standard-DHS_2015.cfm?flag=0/ and could be accessed upon a data request subject to non-profit and academic interest only. In another case, the corresponding author of the paper may be contacted.

## Abbreviations

ANC: Antenatal Care
BCG: Bacillus Calmette Guerin
CCI: Composite Coverage Index
CGI: Coverage Gap Index
DPT: Diphtheria, Pertussis and Tetanus Toxoids
EAG: Empowered Action Group
HDI: Human Development Index
LISA: Local Indicator of Spatial Association
LMIC: Low and Middle Income Countries
MDGs: Millennium Development Goals
NCD: Non-Communicable Diseases
NFHS: National Family Health Survey
NHM: National Health Mission
ORT: Oral Rehydration Therapy
RCH: Reproductive and Child Health Programme
RMNCH: Reproductive Maternal Neonatal and Child Health
SBA: Skilled Birth Attendant
SDGs: Sustainable Development Goals
SDI: Socio-Economic Demographic Development Index
