# Supplementary material for: District level inequality in reproductive, maternal, neonatal and child health coverage in India
Source: BMC Public Health. 2020 Jan 14;20:58. doi: 10.1186/s12889-020-8151-9 (PMC6961337; doi:10.1186/s12889-020-8151-9)
Supplement: Supplementary file 1 — Additional file 1: Percentage distribution of selected variables and asset index by districts of India 2015–16. [file 12889_2020_8151_MOESM1_ESM.pdf]

S1 Table : Coverage gap index (CGI) and eight continuum of caare indicators by districts of India in 2015-16.

States	Districts	BCG	DPT	Measles	ORT	PNCM	SBA	ANC	FP	CGI
Jammu and Kashmir	Kupwara	98.1	88.9	87.8	56.1	74.1	90.4	87.8	81.0	18.5
	Badgam	96.4	97.7	96.7	40.9	97.3	98.4	95.7	89.9	11.7
	Leh	97.9	89.8	91.4	75.1	100.0	97.2	91.7	87.3	9.6
	Kargil	98.9	89.7	92.9	65.0	75.6	86.6	83.6	87.6	16.0
	Punch	97.8	91.9	89.2	81.6	95.6	81.5	73.8	79.6	15.4
	Rajouri	88.4	68.4	58.4	55.1	69.9	78.6	55.9	58.8	35.1
	Kathua	95.6	95.7	89.8	100.0	100.0	95.4	93.3	88.0	5.9
	Baramula	97.4	93.0	93.4	79.2	81.5	91.1	95.2	84.4	12.0
	Bandipore	97.7	77.3	92.7	54.8	87.7	89.9	90.3	80.5	18.0
	Srinagar	96.7	96.7	89.3	83.5	90.2	99.6	91.0	90.8	8.0
	Ganderbal	96.2	91.1	94.2	85.8	95.9	96.1	94.5	83.4	9.3
	Pulwama	100.0	94.2	95.7	79.7	61.8	93.9	98.3	91.8	11.3
	Shupiyan	98.6	85.3	88.2	70.0	84.8	92.7	96.4	89.6	12.3
	Anantnag	98.7	90.1	92.0	78.6	95.4	93.3	82.5	82.4	12.5
	Kulgam	97.6	94.5	89.4	68.4	81.6	91.6	95.1	87.8	12.5
	Doda	79.9	67.7	65.8	58.1	82.8	56.5	36.9	57.3	38.8
	Ramban	88.6	76.1	71.6	81.3	94.5	64.1	51.0	69.1	26.9
	Kishtwar	93.2	88.5	84.7	83.5	77.8	67.6	54.4	80.5	22.3
	Udhampur	97.2	96.0	83.9	86.6	79.2	86.7	90.5	90.4	11.2
	Reasi	96.5	84.4	79.1	56.9	59.6	62.8	57.7	78.3	29.3
	Jammu	98.3	93.9	88.4	65.4	62.7	95.2	83.9	84.1	17.2
	Samba	84.1	76.2	80.8	64.9	86.0	89.8	76.7	61.1	25.2
	Chamba	93.5	84.1	84.8	63.0	67.4	60.5	47.5	77.4	29.2

**Himachal Pradesh**	Kangra	95.2	85.2	81.0	73.9	100.0	90.9	81.9	72.4	16.9
	Lahul And Spiti	91.7	76.3	91.7	81.0	43.7	76.6	61.5	84.0	25.2
	Kullu	96.0	85.8	92.4	78.3	100.0	73.5	71.3	80.8	16.9
	Mandi	98.5	87.4	94.4	69.4	90.4	67.3	56.1	80.7	21.4
	Hamirpur	88.3	74.2	77.6	77.3	69.7	91.5	73.6	69.5	24.0
	Una	94.1	79.4	75.5	68.7	93.0	85.5	72.3	63.1	23.8
	Bilaspur	92.7	81.3	86.1	86.8	100.0	84.1	58.8	76.3	18.4
	Solan	91.4	89.3	89.6	84.6	82.3	78.4	77.6	88.1	15.1
	Sirmaur	90.8	90.9	98.3	43.7	100.0	76.6	59.8	81.7	21.4
	Shimla	100.0	91.8	97.8	65.2	58.7	78.8	78.9	89.2	18.7
	Kinnaur	100.0	96.3	100.0	55.3	84.4	82.7	67.3	85.8	17.8
**Punjab**	Gurdaspur	100.0	94.9	96.1	78.4	100.0	95.1	63.4	94.0	10.3
	Kapurthala	100.0	100.0	100.0	92.7	96.8	97.0	76.9	90.0	7.1
	Jalandhar	100.0	96.1	97.0	86.7	100.0	97.7	71.4	86.4	9.6
	Hoshiarpur	97.8	95.3	95.2	59.3	91.1	95.9	69.8	88.9	14.3
	Sangrur	97.8	94.1	97.8	72.7	87.8	97.0	89.5	84.2	11.6
	Fatehgarh Sahib	96.4	91.8	94.6	50.2	96.6	95.6	63.7	92.3	15.2
	Ludhiana	94.6	88.1	82.5	72.0	59.8	88.6	69.9	94.2	18.1
	Moga	100.0	100.0	94.0	70.2	100.0	97.9	69.7	92.6	10.0
	Firozpur	96.7	92.3	88.6	63.1	100.0	92.9	66.7	95.4	12.7
	Muktsar	98.3	98.3	96.9	80.6	100.0	97.7	64.9	98.0	8.1
	Faridkot	100.0	100.0	97.8	95.8	93.5	98.8	83.9	93.7	5.2
	Bathinda	95.0	92.6	94.1	47.8	89.6	95.6	66.7	96.0	15.1
	Mansa	97.1	93.6	95.4	65.6	86.0	96.2	65.9	94.1	13.5
	Patiala	100.0	98.9	97.3	64.5	92.8	94.1	63.5	96.2	11.9

	Amritsar	98.5	96.8	91.9	65.8	95.5	92.5	76.1	95.8	10.8
	Tarn Taran	100.0	98.2	98.3	94.8	93.7	95.5	50.1	96.3	9.5
	Rupnagar	100.0	97.4	97.8	65.1	91.6	93.7	74.8	87.8	12.9
	Sahibzada Ajit Singh	98.4	94.4	94.4	75.0	96.6	88.8	79.9	92.3	10.5
	Shahid Bhagat Singh N	98.0	85.5	87.1	79.7	93.7	91.1	54.7	84.9	16.6
	Barnala	100.0	98.7	90.9	78.3	95.3	98.6	57.5	94.2	11.0
**Chandigarh**	Chandigarh	95.9	95.9	95.9	70.6	92.3	93.3	64.5	93.0	12.7
**Uttarakhand**	Uttarkashi	95.1	86.1	95.1	70.7	93.1	65.5	22.2	86.8	24.2
	Chamoli	93.2	76.4	81.6	76.8	57.3	61.8	20.3	81.4	32.1
	Rudraprayag	95.5	85.7	87.2	77.6	78.3	67.6	17.2	84.4	26.7
	Tehri Garhwal	98.5	85.4	85.6	63.0	81.1	71.9	23.8	80.8	27.7
	Dehradun	94.0	85.8	87.5	68.4	87.2	85.2	47.1	84.0	20.9
	Garhwal	96.4	78.2	80.1	74.2	87.5	74.9	36.4	81.3	24.8
	Pithoragarh	96.8	89.9	90.3	40.1	69.4	75.6	30.8	86.4	28.5
	Bageshwar	89.6	89.6	80.5	52.5	58.9	62.3	23.4	76.9	34.3
	Almora	97.8	83.7	85.5	67.5	57.4	69.6	31.7	79.9	29.8
	Champawat	98.9	93.7	86.3	55.4	68.4	74.0	29.0	82.4	27.8
	Nainital	91.6	77.2	78.5	67.5	83.8	70.2	40.4	73.4	28.6
	Udham Singh Nagar	92.4	76.0	78.1	57.8	80.4	72.2	26.6	67.5	33.3
	Hardwar	88.2	75.0	71.9	73.5	92.9	63.6	24.2	70.7	31.2
	Panchkula	100.0	100.0	100.0	100.0	100.0	97.3	81.5	96.9	3.4
	Ambala	100.0	100.0	97.4	85.1	77.2	97.6	64.7	97.8	10.2
	Yamunanagar	100.0	94.7	98.4	91.5	91.0	97.4	75.4	95.0	7.6
	Kurukshetra	100.0	95.8	100.0	91.8	100.0	95.2	65.0	92.2	8.5
	Kaithal	98.5	94.5	93.1	88.3	94.6	89.5	61.0	93.6	11.1

**Haryana**	Karnal	100.0	97.9	93.5	79.2	100.0	96.4	63.1	94.5	9.7
	Panipat	100.0	89.2	85.2	92.6	100.0	86.1	40.3	94.7	13.7
	Sonipat	96.6	81.3	88.7	72.9	100.0	84.1	38.7	93.8	17.9
	Jind	97.7	93.7	92.8	87.2	83.9	94.6	57.1	96.0	12.0
	Fatehabad	98.7	97.5	95.8	74.6	89.0	97.0	60.7	97.2	11.2
	Sirsa	100.0	88.9	87.1	61.5	84.1	91.7	55.9	93.2	17.3
	Hisar	96.9	90.5	83.7	67.2	70.0	93.6	40.5	91.2	20.7
	Bhiwani	96.6	86.7	78.8	68.1	79.3	91.2	53.8	92.1	18.6
	Rohtak	99.0	81.2	85.6	74.7	100.0	89.1	48.4	91.2	16.5
	Jhajjar	97.2	76.9	70.2	62.4	91.3	89.7	52.7	91.9	19.9
	Mahendragarh	100.0	90.1	90.9	82.7	70.3	98.4	44.3	90.9	17.1
	Rewari	94.0	61.4	76.8	28.8	90.0	91.7	28.7	78.8	32.0
	Gurgaon	98.5	45.9	72.3	81.1	96.1	72.7	33.1	78.6	28.6
	Mewat	62.3	25.2	32.3	55.0	72.0	45.6	6.5	34.1	60.0
	Faridabad	84.7	67.6	64.7	61.6	81.8	80.9	30.1	57.9	35.9
	Palwal	68.6	47.7	53.1	52.0	80.4	64.1	19.0	63.1	43.7
**Delhi**	North West	100.0	83.5	88.6	64.6	100.0	78.5	65.7	77.8	19.7
	North	91.3	62.7	84.5	73.8	53.3	92.7	71.2	69.1	27.5
	North East	88.9	73.4	85.6	72.0	63.9	80.4	59.3	79.0	25.7
	East	97.2	78.5	82.1	60.8	71.6	84.3	60.3	68.0	27.4
	New Delhi	95.4	85.9	85.9	83.7	100.0	87.7	74.8	70.3	17.1
	Central	92.8	79.6	82.1	87.3	91.8	99.3	81.2	77.4	26.3
	West	94.2	94.2	94.2	72.0	83.7	94.4	80.6	81.7	14.7
	South West	95.9	94.0	97.1	71.6	73.6	89.9	68.5	87.0	16.5
	South	95.1	92.1	97.3	77.3	100.0	88.6	69.3	72.9	16.3

	Ganganagar	97.8	90.9	93.0	75.2	84.2	90.8	52.1	89.4	16.6
	Hanumangarh	92.2	84.2	89.9	54.8	78.4	90.3	24.7	88.6	24.9
	Bikaner	87.3	69.9	73.8	58.4	87.5	80.8	38.2	89.7	25.7
	Churu	94.1	79.6	83.7	55.6	69.1	86.0	18.3	77.5	31.0
	Jhunjhunun	94.6	82.5	89.4	68.7	86.7	98.0	45.4	83.7	19.9
	Alwar	80.7	69.3	78.4	52.9	92.5	81.0	21.8	83.7	29.5
	Bharatpur	77.5	61.4	70.3	41.9	74.2	79.6	17.2	71.7	38.5
	Dhaulpur	90.8	70.8	77.2	39.4	61.7	85.8	30.7	78.1	33.9
	Karauli	92.8	67.9	79.1	76.1	77.0	83.6	29.3	80.8	27.3
	Sawai Madhopur	82.7	62.9	71.3	42.5	83.3	89.4	33.8	77.0	32.1
	Dausa	96.1	81.4	84.0	61.9	93.3	91.2	28.4	80.0	24.2
	Jaipur	98.0	76.9	87.3	70.4	86.5	95.2	58.7	83.8	19.0
	Sikar	90.7	69.1	82.0	72.6	91.3	95.3	49.1	79.4	22.2
	Nagaur	83.9	62.8	76.1	72.2	75.1	90.9	42.2	78.9	27.4
	Jodhpur	84.9	63.1	75.2	57.5	84.8	78.0	40.2	84.8	28.3
	Jaisalmer	81.3	55.9	56.5	60.5	100.0	62.0	18.4	80.6	34.1
**Rajasthan**	Barmer	68.8	51.4	49.7	55.6	85.0	70.5	16.2	76.3	38.7
	Jalor	74.1	46.6	52.9	57.4	87.7	87.9	31.0	82.0	32.7
	Sirohi	76.8	66.2	65.0	59.4	100.0	86.0	31.7	77.1	29.0
	Pali	93.5	68.6	75.4	41.5	71.6	85.5	47.9	83.1	29.3
	Ajmer	93.8	86.2	83.0	81.8	100.0	88.4	51.4	90.0	15.5
	Tonk	98.7	92.8	93.2	44.5	100.0	95.8	49.5	87.0	18.4
	Bundi	95.8	75.0	85.9	72.4	90.2	93.6	30.3	85.2	22.1
	Bhilwara	96.2	90.7	87.0	58.5	83.0	84.2	41.9	82.1	23.2
	Rajsamand	97.5	78.0	83.8	67.3	78.9	87.4	39.2	82.1	24.3

Dungarpur	93.7	75.8	83.8	84.1	86.3	87.1	45.9	87.0	19.8
Banswara	90.2	70.9	77.4	69.4	75.6	95.6	43.4	85.4	23.8
Chittaurgarh	95.8	60.0	80.7	40.2	100.0	88.8	22.7	76.6	30.8
Kota	97.8	85.3	86.0	72.3	83.1	92.9	58.7	91.1	16.7
Baran	99.0	81.1	95.2	73.2	100.0	96.5	46.2	89.0	16.0
Jhalawar	96.7	85.9	91.4	83.3	79.1	95.3	36.5	88.2	18.7
Udaipur	80.5	65.0	66.3	63.5	100.0	74.8	45.9	80.5	27.1
Pratapgarh	97.5	79.3	83.5	70.3	88.5	90.8	30.7	87.5	21.9
Saharanpur	92.0	81.1	85.9	52.2	83.7	66.9	41.7	87.6	26.3
Muzaffarnagar	88.4	71.4	70.4	62.0	73.1	70.1	37.0	85.5	29.5
Bijnor	95.3	80.1	88.3	60.5	100.0	74.8	24.9	80.9	25.8
Moradabad	85.4	65.2	73.9	39.6	77.8	66.4	44.8	86.9	31.6
Rampur	98.4	74.2	78.0	57.9	76.5	65.5	59.4	87.5	25.4
Jyotiba Phule Nagar	93.1	84.3	84.4	61.6	90.2	71.2	33.1	84.3	25.3
Meerut	88.1	70.7	70.4	60.2	84.4	70.1	47.1	90.6	25.9
Baghpat	94.9	84.6	79.7	66.9	95.0	79.3	50.7	91.1	19.3
Ghaziabad	90.7	76.3	79.4	46.3	78.3	72.0	39.7	89.1	28.0
Gautam Buddha Nagar	93.1	76.7	75.8	57.1	73.1	74.1	51.2	92.2	24.9
Bulandshahr	91.3	79.7	77.7	44.5	100.0	72.2	23.1	81.2	29.2
Aligarh	94.6	79.8	84.4	35.2	68.4	73.9	28.0	83.7	32.2
Mahamaya Nagar	90.1	72.8	72.7	24.5	66.4	79.6	24.4	83.6	35.4
Mathura	90.4	63.1	72.4	48.8	92.2	75.5	27.5	80.2	31.4
Agra	94.8	74.9	83.4	32.6	71.5	77.7	37.2	86.3	30.6
Firozabad	91.8	72.9	75.7	44.6	72.3	67.6	24.7	74.5	35.6
Mainpuri	88.1	71.6	77.3	49.6	89.0	63.6	13.7	64.9	37.5

	Budaun	88.8	60.5	66.7	43.8	79.4	61.8	12.6	82.4	37.4
	Bareilly	82.4	62.2	63.6	38.8	61.5	62.6	45.5	91.2	34.2
	Pilibhit	96.8	78.9	83.1	45.6	88.4	68.6	42.2	88.6	26.1
	Shahjahanpur	90.6	79.5	76.2	59.1	91.0	54.0	21.6	78.3	31.9
	Kheri	89.9	74.6	76.0	51.2	72.8	58.4	13.5	56.7	41.6
	Sitapur	87.3	54.5	72.9	45.6	73.0	71.9	10.2	72.7	39.9
	Hardoi	80.6	57.8	61.4	51.5	64.3	67.6	10.3	51.9	46.7
	Unnao	86.5	63.7	67.4	18.1	73.1	70.7	11.8	68.3	43.6
	Lucknow	92.8	72.9	79.9	36.2	77.6	88.6	51.6	78.4	28.7
	Rae Bareli	95.0	78.1	76.9	71.1	87.9	82.0	26.4	55.6	32.2
	Farrukhabad	79.8	60.5	60.2	29.8	68.5	53.6	17.2	71.7	44.6
	Kannauj	86.7	60.0	67.4	36.9	63.3	63.3	11.4	62.1	45.5
	Etawah	86.1	63.9	66.9	30.9	65.9	75.8	24.2	74.1	39.3
	Auraiya	81.4	58.8	67.3	37.7	92.4	72.5	11.7	72.4	38.5
	Kanpur Dehat	96.2	68.5	75.8	47.0	93.8	75.0	6.9	72.6	34.7
	Kanpur Nagar	95.4	78.3	74.0	71.6	59.1	79.3	36.0	84.4	27.8
	Jalaun	85.0	72.6	71.9	33.9	34.3	79.9	17.1	76.1	41.4
	Jhansi	98.5	77.7	80.0	56.8	87.3	87.0	39.2	88.9	23.1
**Uttar Pradesh**	Lalitpur	95.6	75.7	82.7	41.6	85.9	85.4	27.0	88.5	27.3
	Hamirpur	82.6	72.2	73.9	33.3	100.0	86.6	18.1	71.5	33.6
	Mahoba	95.6	69.1	77.6	38.3	80.5	91.6	23.6	91.4	28.4
	Banda	94.5	61.5	72.1	55.3	60.9	76.3	6.4	81.8	36.6
	Chitrakoot	95.0	83.3	82.4	62.7	82.3	77.5	16.3	72.1	30.6
	Fatehpur	87.0	69.8	71.5	40.1	66.3	69.0	9.6	69.6	40.8
	Pratapgarh	89.1	69.9	77.8	53.6	76.2	81.1	18.5	65.8	35.7

Kaushambi	88.5	56.9	63.0	41.9	92.4	74.6	12.3	60.6	40.6
Allahabad	90.1	65.5	67.4	54.2	92.7	76.8	29.7	62.5	34.7
Bara Banki	90.4	59.4	59.8	43.7	74.7	62.8	23.6	67.2	40.8
Faizabad	90.9	70.0	66.0	46.2	64.2	77.8	20.1	64.6	39.3
Ambedkar Nagar	93.2	74.6	78.2	35.8	43.4	84.2	37.3	62.2	39.3
Sultanpur	87.8	60.8	74.5	27.2	64.7	74.0	21.8	59.4	43.9
Bahraich	44.0	16.1	27.0	35.8	57.8	36.8	4.3	27.2	69.9
Shrawasti	58.1	27.4	37.3	34.4	63.8	49.6	8.3	22.6	65.5
Balrampur	54.2	18.2	31.4	53.2	72.0	40.1	10.8	8.0	68.4
Gonda	74.6	51.3	53.9	49.8	63.9	57.7	13.5	30.5	54.8
Siddharth Nagar	78.6	48.2	55.9	40.7	71.6	44.3	14.8	49.7	51.7
Basti	91.1	69.4	80.1	50.8	69.5	76.6	19.8	38.5	43.9
Sant Kabir Nagar	89.1	61.4	70.2	38.1	77.6	65.9	32.2	44.5	44.5
Mahrajganj	87.4	70.2	70.5	69.3	79.1	65.3	25.2	55.5	37.6
Gorakhpur	96.8	80.8	86.1	42.3	79.4	76.0	35.2	66.3	32.8
Kushinagar	82.9	60.2	63.5	59.8	88.0	71.9	25.9	56.7	38.5
Deoria	93.0	76.6	74.6	50.3	81.3	85.1	25.6	58.4	35.1
Azamgarh	84.1	65.8	60.1	39.7	84.5	83.1	23.7	59.5	39.0
Mau	88.2	65.3	73.1	39.1	76.4	81.5	39.0	63.9	36.3
Ballia	85.4	61.1	59.8	43.9	86.3	72.1	37.7	59.1	38.5
Jaunpur	91.0	69.5	72.3	38.2	75.0	76.6	28.3	61.6	38.4
Ghazipur	90.9	61.8	73.1	24.7	74.5	77.7	19.2	64.6	41.4
Chandauli	90.9	77.6	81.9	68.9	82.9	77.4	25.1	63.8	31.8
Varanasi	93.3	78.1	77.8	61.1	86.6	85.2	33.3	78.3	26.7
Sant Ravidas Nagar (B	86.8	61.5	67.8	62.5	55.2	85.4	38.7	66.9	35.7

	Mirzapur	90.6	75.3	75.2	49.9	56.2	76.3	15.4	72.8	37.3
	Sonbhadra	80.4	69.8	64.2	46.7	74.6	59.7	22.3	71.5	38.9
	Etah	83.1	62.4	64.0	24.8	56.6	62.5	17.0	80.7	42.7
	Kanshiram Nagar	88.8	63.9	61.8	25.1	60.5	62.8	13.1	83.6	41.5
Bihar	Pashchim Champaran	74.3	65.9	56.7	67.6	51.9	71.0	14.2	16.2	53.9
	Purba Champaran	84.9	69.8	64.9	59.4	59.2	57.4	12.1	20.2	53.4
	Sheohar	94.6	79.5	75.8	54.5	63.1	60.0	13.4	43.3	44.7
	Sitamarhi	87.4	79.8	82.6	51.2	63.6	46.2	16.6	64.2	41.2
	Madhubani	91.9	70.9	72.5	61.8	82.2	62.7	13.8	42.9	42.6
	Supaul	93.7	82.8	82.4	48.9	68.3	69.3	11.0	65.9	37.5
	Araria	89.6	76.7	72.6	61.5	60.1	61.9	16.2	61.7	39.9
	Kishanganj	85.9	73.4	69.0	73.3	86.8	58.0	15.5	33.7	43.5
	Purnia	95.9	86.8	89.5	52.9	65.1	62.1	12.2	59.1	38.8
	Katihar	96.5	85.6	86.4	49.5	51.4	57.2	8.9	55.5	43.1
	Madhepura	95.4	82.1	78.2	46.8	56.6	64.2	9.3	58.5	42.2
	Saharsa	96.8	87.6	88.8	55.8	67.7	63.1	10.1	61.0	37.6
	Darbhanga	91.5	72.0	75.4	54.1	54.9	59.7	9.4	43.3	47.5
	Muzaffarpur	90.1	75.8	79.1	44.4	63.0	65.6	10.9	33.7	48.5
	Gopalganj	92.1	80.9	80.2	50.2	72.7	78.6	20.1	28.8	44.2
	Siwan	85.0	75.7	76.9	52.1	59.1	77.9	21.3	29.4	46.8
	Saran	87.9	71.5	69.0	61.8	64.2	77.0	20.5	25.9	46.9
	Vaishali	93.4	83.8	83.0	63.0	53.9	82.9	19.3	54.6	37.5
	Samastipur	89.3	83.1	75.7	57.4	66.1	75.5	11.6	35.9	44.0
	Begusarai	99.5	89.9	92.4	71.6	86.6	78.8	7.9	63.1	30.4
	Khagaria	97.5	89.3	83.5	53.4	88.0	77.1	13.2	57.4	34.2

	Bhagalpur	98.3	88.4	85.4	70.5	71.1	75.0	20.1	56.0	33.9
	Banka	97.0	80.2	86.7	73.1	62.8	73.0	16.8	59.5	35.4
	Munger	94.0	91.0	83.3	69.9	76.7	85.4	24.4	64.0	29.5
	Lakhisarai	89.3	80.1	68.5	69.4	70.0	67.6	17.9	61.9	36.5
	Sheikhpura	91.7	83.5	82.2	60.4	78.1	81.6	13.4	62.8	33.8
	Nalanda	94.5	82.8	84.7	57.4	54.2	82.6	9.0	57.3	38.7
	Patna	88.8	85.6	84.2	56.4	74.7	84.2	21.7	70.3	31.3
	Bhojpur	93.0	86.7	82.5	47.5	90.2	81.3	16.1	60.2	33.8
	Buxar	89.6	83.8	77.2	65.2	61.3	83.6	23.3	67.9	32.9
	Kaimur (Bhabua)	96.7	90.7	85.2	36.5	69.4	82.9	10.1	64.3	36.4
	Rohtas	99.0	93.6	89.9	53.5	95.2	83.2	13.9	73.3	27.4
	Aurangabad	96.7	95.4	88.3	21.9	69.7	74.0	16.2	62.6	38.1
	Gaya	95.3	81.4	85.5	39.2	71.0	69.0	11.8	66.1	38.1
	Nawada	95.6	82.6	83.0	53.9	63.8	72.0	13.6	59.4	38.3
	Jamui	83.8	71.7	69.3	53.2	68.3	65.3	10.4	54.0	43.3
	Jehanabad	96.5	89.4	88.2	61.1	71.5	83.6	17.0	67.1	31.4
	Arwal	100.0	91.1	91.1	45.6	55.5	70.6	11.5	58.4	39.2
**Sikkim**	North District	100.0	98.0	100.0	46.0	100.0	97.3	73.7	72.3	17.5
	West District	97.1	94.2	89.9	100.0	100.0	95.7	84.9	82.0	8.5
	South District	100.0	97.4	100.0	89.0	100.0	100.0	84.7	76.0	33.2
	East District	98.9	89.6	90.3	78.0	100.0	96.5	67.4	56.2	20.2
	Tawang	65.3	38.8	46.8	31.2	38.1	33.0	14.0	43.4	62.8
	West Kameng	67.7	43.6	47.8	85.8	35.4	56.8	33.3	36.2	51.9
	East Kameng	37.2	17.4	17.4	89.4	39.6	35.8	0.8	19.9	68.7
	Papumpare	80.1	59.4	56.9	82.9	67.3	81.6	26.0	29.6	44.4

Arunachal Pradesh	Upper Subansiri	60.4	31.1	41.9	81.7	80.4	47.0	15.1	44.1	50.7
	West Siang	74.4	44.0	70.8	81.8	53.6	52.0	18.0	26.7	53.1
	East Siang	83.6	59.1	78.8	96.7	35.2	70.7	35.0	62.8	37.1
	Upper Siang	93.8	61.5	40.9	73.4	0.0	69.0	29.2	75.4	43.6
	Changlang	93.8	83.7	83.7	30.7	41.2	52.4	38.9	85.7	36.6
	Tirap	63.0	65.6	63.0	37.7	11.6	34.6	29.8	85.2	48.4
	Lower Subansiri	68.8	56.1	53.0	61.8	0.0	64.1	22.4	33.6	58.4
	Kurung Kumey	49.2	28.6	28.2	75.9	33.5	31.6	7.9	39.2	63.2
	Dibang Valley	82.8	72.8	67.4	90.5	77.4	57.2	22.4	76.5	31.5
	Lower Dibang Valley	74.4	53.0	57.2	51.5	100.0	58.9	23.5	66.6	39.3
	Lohit	84.5	73.7	66.0	41.2	65.3	48.0	49.2	87.0	34.2
	Anjaw	75.4	54.5	56.8	93.4	40.2	57.9	20.2	50.4	45.9
Nagaland	Mon	48.2	31.1	31.0	21.4	23.4	14.4	3.6	37.0	74.1
	Mokokchung	77.1	77.8	75.8	26.8	51.9	78.9	35.8	67.9	39.6
	Zunheboto	71.4	42.3	48.2	28.2	0.0	25.7	5.9	65.1	63.5
	Wokha	50.9	46.9	54.7	33.6	49.6	59.2	25.1	56.4	52.5
	Dimapur	63.3	54.6	47.4	57.7	44.6	61.3	26.5	53.6	49.1
	Phek	74.5	55.9	47.3	54.6	0.0	25.1	6.6	54.0	61.1
	Tuensang	77.7	59.6	56.6	36.2	21.4	34.4	4.6	44.4	61.0
	Longleng	55.4	28.2	37.9	23.4	100.0	17.9	1.5	56.9	58.6
	Kiphire	65.7	44.5	50.2	73.6	10.6	30.0	2.0	64.1	56.7
	Kohima	93.9	77.2	71.2	65.5	18.8	63.0	30.5	63.7	41.9
	Peren	81.1	57.7	51.7	83.1	29.0	56.8	13.1	55.4	47.9
	Senapati (Excluding 3	90.7	71.8	69.3	69.6	33.1	52.6	39.5	37.0	47.4
	Tamenglong	82.0	60.8	56.4	49.7	19.9	42.1	32.7	36.1	56.7

**Manipur**	Churachandpur	84.1	68.4	65.1	62.3	12.1	67.7	60.1	50.9	44.1
	Bishnupur	96.1	87.3	82.3	63.4	38.0	92.5	87.0	54.5	29.2
	Thoubal	90.8	77.4	74.3	64.5	52.9	84.6	79.7	42.4	34.2
	Imphal West	97.2	93.7	87.9	76.4	42.7	95.1	78.8	39.2	30.3
	Imphal East	95.9	81.4	81.0	62.4	47.1	86.1	82.1	53.3	30.7
	Ukhrul	78.6	54.2	50.3	64.2	33.9	43.4	27.0	30.0	56.6
	Chandel	86.2	68.2	58.1	80.7	41.6	62.4	42.9	34.7	45.3
**Mizoram**	Mamit	64.2	54.9	56.8	70.9	100.0	67.6	44.8	60.3	35.1
	Kolasib	64.1	61.5	56.4	83.3	65.3	88.4	66.8	64.9	30.6
	Aizawl	82.8	65.0	60.4	85.6	69.3	97.7	76.6	69.9	24.3
	Champhai	72.6	59.6	64.6	75.0	63.2	86.8	55.9	57.9	34.4
	Serchhip	82.0	72.4	74.3	55.1	35.6	93.8	68.2	69.0	32.3
	Lunglei	69.2	60.9	66.4	72.1	78.7	77.8	49.5	61.1	33.9
	Lawngtlai	70.7	53.4	48.9	49.1	24.5	49.7	38.3	60.1	50.6
	Saiha	79.1	69.1	75.2	61.8	76.2	70.2	48.2	58.6	35.0
**Tripura**	West Tripura	86.0	72.2	72.7	46.4	71.1	89.9	69.8	83.7	25.5
	South Tripura	86.7	74.1	72.8	48.0	82.8	80.6	59.1	88.1	24.9
	Dhalai	62.1	69.8	62.4	79.3	79.9	77.3	66.4	90.9	22.9
	North Tripura	79.6	66.4	63.6	44.7	64.9	65.7	55.9	88.9	31.6
**Meghalay**	West Garo Hills	87.4	70.9	61.7	73.8	63.1	46.9	37.2	73.5	35.8
	East Garo Hills	60.7	53.5	46.3	71.9	53.9	50.2	29.6	38.0	51.4
	South Garo Hills	91.0	93.9	85.1	97.7	80.7	89.0	51.7	70.2	19.8
	West Khasi Hills	91.2	83.2	85.2	82.5	89.5	40.8	44.3	47.4	34.6
	Ribhoi	84.4	71.5	73.2	91.4	94.4	46.4	40.5	51.5	34.3
	East Khasi Hills	92.2	77.5	78.8	87.6	83.5	69.5	72.7	53.2	27.2

	Jaintia Hills	91.0	79.7	76.6	90.2	95.5	46.0	52.7	50.7	31.4
**Assam**	Kokrajhar	82.1	67.7	66.2	0.0	41.5	72.7	39.1	83.6	42.2
	Dhubri	62.8	30.7	38.5	64.2	39.3	55.7	26.0	62.9	51.0
	Goalpara	83.0	64.4	71.9	54.3	65.3	77.2	42.1	73.1	34.1
	Barpeta	67.4	54.7	66.0	28.6	45.5	63.4	47.5	85.2	40.4
	Morigaon	92.2	68.6	79.6	44.0	46.8	72.9	43.1	79.6	34.9
	Nagaon	80.0	55.1	68.1	33.7	86.6	68.8	46.2	77.3	35.1
	Sonitpur	82.6	65.9	70.6	83.4	72.9	89.2	42.0	88.4	24.2
	Lakhimpur	89.3	80.1	78.7	56.7	82.8	85.8	59.0	72.2	25.9
	Dhemaji	96.4	83.5	79.6	57.4	53.1	80.0	49.1	75.6	29.7
	Tinsukia	89.4	83.8	89.8	49.0	72.3	76.9	56.1	83.1	25.8
	Dibrugarh	100.0	83.9	92.6	72.9	78.1	90.7	67.6	80.4	18.7
	Sivasagar	100.0	84.4	88.6	71.7	48.7	88.8	70.8	76.1	23.6
	Jorhat	92.2	84.0	91.4	81.7	77.2	97.1	75.8	82.6	15.9
	Golaghat	90.9	83.7	91.0	66.9	78.4	89.6	62.5	74.1	22.5
	Karbi Anglong	68.5	46.6	53.1	32.8	36.1	55.7	38.1	79.9	46.3
	Dima Hasao	91.4	79.4	86.3	81.3	73.3	60.2	35.1	73.3	29.4
	Cachar	82.2	71.9	68.7	73.7	77.0	75.3	50.9	81.5	26.6
	Karimganj	80.5	72.1	66.1	51.4	100.0	48.9	37.1	79.1	32.4
	Hailakandi	68.3	59.5	59.5	33.1	51.7	56.9	34.5	76.7	43.4
	Bongaigaon	84.7	70.6	79.9	79.3	43.2	70.4	24.2	86.2	32.2
	Chirang	77.7	60.1	68.5	33.1	25.1	62.7	41.2	88.8	40.9
	Kamrup	72.2	58.0	57.4	65.9	59.4	87.7	40.4	66.9	36.3
	Kamrup Metropolitan	93.7	85.5	91.5	86.5	69.4	92.4	56.6	73.0	21.4
	Nalbari	89.4	83.0	79.7	73.9	63.3	84.7	49.2	90.8	22.5

	Baksa	91.6	82.6	81.3	47.6	50.9	89.3	49.8	82.5	28.5
	Darrang	81.2	63.3	61.9	29.7	49.4	69.5	39.9	91.6	36.7
	Udalguri	90.6	79.6	82.0	48.8	50.7	76.4	37.0	88.5	30.5
**West Bengal**	Darjiling	95.4	91.7	89.5	79.9	65.3	94.6	65.9	91.3	16.0
	Jalpaiguri	98.3	91.2	88.9	84.0	75.3	86.5	80.7	78.5	16.5
	Koch Bihar	98.1	95.8	95.8	84.8	94.8	88.7	74.4	88.2	11.0
	Uttar Dinajpur	92.6	75.4	73.5	76.6	69.4	58.4	43.1	79.7	29.3
	Dakshin Dinajpur	96.3	90.9	88.6	68.0	55.1	80.3	68.6	88.8	20.9
	Maldah	94.2	86.9	86.4	61.5	72.4	61.6	52.6	81.9	26.4
	Murshidabad	95.8	91.6	93.8	67.3	68.7	69.8	72.1	91.8	19.0
	Birbhum	100.0	91.4	98.5	93.3	100.0	88.2	78.1	95.1	7.4
	Barddhaman	96.3	91.8	92.9	79.7	88.0	88.0	83.3	94.3	10.8
	Nadia	100.0	100.0	98.4	67.4	100.0	90.8	91.6	91.8	8.4
	North Twenty Four Par	96.7	94.2	93.4	64.5	83.2	93.2	79.3	91.7	13.4
	Hugli	100.0	100.0	95.3	79.3	25.5	91.7	76.6	92.7	18.0
	Bankura	100.0	100.0	98.2	46.6	93.8	87.9	89.5	93.5	12.0
	Puruliya	96.3	93.5	93.9	76.1	63.8	79.9	68.6	89.1	18.1
	Haora	97.5	86.6	83.8	92.5	100.0	92.2	86.6	88.3	9.4
	Kolkata	100.0	94.5	85.2	76.3	91.0	97.3	84.6	91.6	10.1
	South Twenty Four Par	98.3	94.8	98.3	67.4	84.4	67.2	75.6	90.6	16.4
	Paschim Medinipur	100.0	98.1	96.1	92.8	73.9	84.6	84.0	94.2	10.0
	Purba Medinipur	97.5	95.1	95.1	65.6	63.0	87.8	79.3	92.9	15.9
	Garhwa	88.1	74.9	78.3	61.0	60.0	65.4	11.6	59.9	40.5
	Chatra	90.2	58.9	66.9	67.4	73.8	62.4	11.8	65.0	39.6
	Kodarma	95.9	89.2	84.8	46.4	92.0	84.9	57.4	85.4	21.1

Jharkhan	Giridih	97.4	73.0	82.7	68.4	74.6	73.1	35.9	72.6	30.0
	Deoghar	95.3	86.7	81.1	29.6	75.0	62.0	25.5	72.2	36.1
	Godda	93.4	87.4	79.4	55.4	75.0	66.0	28.8	74.8	31.4
	Sahibganj	95.5	76.5	75.5	48.6	55.2	55.2	20.0	67.5	40.5
	Pakur	99.2	87.5	93.2	44.6	51.3	55.5	29.7	65.0	38.1
	Dhanbad	98.7	90.1	84.8	57.0	72.8	76.1	40.2	81.1	26.2
	Bokaro	100.0	87.3	87.0	54.3	66.8	74.1	38.5	76.1	29.2
	Lohardaga	96.1	83.7	77.7	60.0	30.8	75.3	16.7	53.4	42.5
	Purbi Singhbhum	99.1	90.3	86.4	67.0	93.2	85.7	50.6	68.3	23.0
	Palamu	96.2	74.2	78.6	39.1	73.9	66.1	24.1	58.8	39.7
	Latehar	84.3	71.1	70.3	56.5	71.0	60.1	15.0	57.4	41.8
	Hazaribagh	100.0	90.5	97.1	72.6	57.9	83.2	39.1	79.5	24.9
	Ramgarh	98.7	85.8	92.9	51.9	73.2	81.5	34.1	75.9	28.2
	Dumka	97.2	95.9	88.0	51.1	56.4	61.4	29.0	66.4	35.1
	Jamtara	94.9	80.2	74.9	60.0	56.9	73.6	29.3	72.9	33.7
	Ranchi	98.3	94.9	85.4	46.9	75.2	84.0	39.9	71.3	28.1
	Khunti	95.3	95.6	91.2	54.7	54.6	75.9	43.0	68.3	30.8
	Gumla	93.1	75.8	83.5	49.6	59.3	74.2	24.4	51.5	40.7
	Simdega	94.1	76.8	85.9	42.4	69.6	55.6	12.8	51.0	43.8
	Pashchimi Singhbhum	91.8	72.7	67.8	62.7	55.6	40.6	12.6	39.6	49.6
	Saraikela Kharsawan	97.7	91.4	91.3	66.3	64.5	70.9	25.9	56.1	34.3
	Bargarh	92.5	87.5	86.6	100.0	71.4	91.5	63.5	87.9	15.1
	Jharsuguda	95.5	92.6	92.4	72.6	53.4	93.9	74.5	94.1	16.4
	Sambalpur	93.1	86.4	86.5	83.9	87.9	92.2	83.0	85.7	13.2
	Debagarh	96.7	90.2	86.4	73.5	66.2	86.7	58.4	72.2	23.6

**Odisha**	Sundargarh	97.8	95.7	95.7	73.7	78.7	89.4	80.7	87.6	13.7
	Kendujhar	89.7	83.1	83.9	69.3	37.4	72.0	39.4	62.8	35.8
	Mayurbhanj	88.5	83.3	88.5	67.3	68.9	84.9	71.6	73.3	23.6
	Baleshwar	92.9	90.1	87.6	79.9	70.6	87.0	57.3	64.1	24.6
	Bhadrak	96.2	92.3	93.6	74.5	76.7	85.9	34.8	57.4	28.3
	Kendrapara	86.7	85.8	83.3	73.8	77.5	95.8	53.1	82.7	20.5
	Jagatsinghapur	96.2	92.7	96.2	82.7	82.1	96.4	80.4	82.3	13.1
	Cuttack	96.3	93.0	93.5	72.4	82.1	92.9	51.0	77.0	20.0
	Jajapur	96.8	93.5	94.9	63.5	58.8	93.2	50.8	80.1	23.0
	Dhenkanal	100.0	100.0	98.2	69.2	63.2	93.3	69.9	89.1	15.9
	Anugul	100.0	99.2	94.3	69.2	93.1	92.8	68.4	87.0	13.3
	Nayagarh	95.8	93.8	90.1	71.8	82.8	93.1	58.1	85.9	17.0
	Khordha	88.3	81.5	81.1	66.4	65.5	84.0	60.5	84.1	23.7
	Puri	98.3	93.3	94.9	59.7	60.0	97.7	81.8	87.2	17.0
	Ganjam	90.9	77.3	71.2	87.9	73.0	91.8	51.3	77.7	22.8
	Gajapati	77.2	66.4	71.0	63.0	90.3	68.4	49.1	88.9	26.4
	Kandhamal	93.9	93.0	84.2	78.3	84.7	76.8	64.8	86.3	17.6
	Baudh	98.3	98.3	98.3	81.2	92.2	87.7	73.4	89.4	11.3
	Subarnapur	100.0	98.1	96.8	78.3	92.6	94.2	85.3	86.6	10.0
	Balangir	100.0	98.1	96.7	90.2	74.8	91.4	76.5	89.7	11.4
	Nuapada	94.7	93.4	87.8	64.4	84.5	87.0	75.5	88.2	15.9
	Kalahandi	98.3	94.8	98.3	92.1	53.2	76.9	46.7	87.5	20.4
	Rayagada	93.1	87.9	76.5	81.7	61.4	80.0	59.3	85.0	21.9
	Nabarangapur	95.1	87.8	86.5	69.2	63.9	68.6	70.5	90.9	20.9
	Koraput	89.8	82.5	75.6	54.9	63.4	73.2	58.4	86.7	26.4

	Malkangiri	98.3	93.8	85.1	88.9	74.2	77.5	69.3	84.9	16.8
**Chhattisgarh**	Korea (Koriya)	97.1	84.0	86.5	84.7	75.5	77.3	45.1	80.5	22.6
	Surguja	97.4	88.1	94.6	58.7	50.5	66.8	41.7	74.8	31.1
	Jashpur	95.2	79.3	82.6	66.0	52.0	66.6	34.6	77.3	32.3
	Raigarh	94.6	89.8	94.8	84.9	86.1	76.7	67.7	80.6	17.4
	Korba	99.1	91.8	94.4	75.8	58.6	72.7	52.9	81.5	23.5
	Janjgir - Champa	98.7	88.2	95.6	78.9	57.9	69.6	44.3	83.3	24.7
	Bilaspur	97.8	96.8	92.6	79.4	83.0	85.1	60.7	89.3	15.1
	Kabirdham	98.7	81.1	94.1	79.6	45.6	56.1	43.6	85.0	28.5
	Rajnandgaon	100.0	98.3	98.3	78.3	100.0	92.0	64.2	89.5	11.1
	Durg	100.0	98.1	97.4	69.6	68.6	82.5	59.9	88.3	18.3
	Raipur	98.4	90.2	93.7	55.3	65.9	78.6	76.3	84.4	21.1
	Mahasamund	98.6	90.6	92.7	67.3	66.0	84.9	60.9	89.7	19.4
	Dhamtari	97.5	98.7	94.1	85.0	89.6	90.3	76.3	92.8	9.8
	Uttar Bastar Kanker	100.0	95.5	98.5	69.3	79.1	83.0	72.1	82.4	17.1
	Bastar	100.0	92.7	94.2	79.9	55.5	74.8	55.8	78.1	23.5
	Narayanpur	95.4	80.7	85.4	82.0	78.1	71.8	40.9	75.1	25.7
	Dakshin Bastar Dantew	99.1	91.6	87.4	81.2	86.7	65.4	60.5	71.4	22.3
	Bijapur	100.0	93.6	96.1	95.7	65.8	81.6	49.0	84.9	18.3
	Sheopur	93.8	70.2	85.0	46.7	50.0	78.3	18.7	81.5	35.5
	Morena	92.3	69.8	82.0	68.3	87.3	85.2	41.2	83.1	24.4
	Bhind	91.7	78.8	82.4	69.3	66.8	86.7	28.0	80.8	27.7
	Gwalior	98.5	78.0	79.2	55.7	65.1	89.5	36.4	80.9	28.1
	Datia	94.1	72.5	81.7	55.4	71.0	80.6	29.5	82.0	29.9
	Shivpuri	94.1	79.9	81.9	53.3	63.7	87.9	26.0	82.2	29.6

Madhya Pradesh	Tikamgarh	89.1	45.7	63.0	55.7	63.6	69.8	18.7	79.7	38.9
	Chhatarpur	88.5	53.7	67.2	61.3	58.0	74.3	19.4	81.4	36.6
	Panna	66.7	52.2	49.2	42.6	73.8	72.1	13.8	73.0	42.7
	Sagar	85.4	66.7	72.9	66.6	61.7	69.8	16.7	79.7	35.0
	Damoh	90.5	72.5	76.3	72.1	79.9	65.7	24.2	73.6	31.9
	Satna	92.0	80.0	84.7	41.5	60.5	82.6	23.1	80.8	32.8
	Rewa	94.3	72.4	82.0	54.6	74.1	76.9	24.4	81.0	31.0
	Umaria	94.6	81.9	85.9	60.0	77.7	84.9	18.1	82.5	27.8
	Neemuch	88.6	66.4	71.4	69.6	79.3	80.5	33.0	61.0	33.6
	Mandsaur	83.8	60.2	63.3	45.8	82.6	88.4	34.8	55.2	38.0
	Ratlam	92.2	65.1	64.1	70.2	72.1	82.1	38.1	62.2	33.7
	Ujjain	91.9	70.8	88.3	63.5	85.5	86.6	40.4	72.4	27.3
	Shajapur	97.1	87.1	92.8	54.0	100.0	96.6	56.5	83.7	17.9
	Dewas	92.9	86.6	79.5	74.6	70.3	84.9	41.3	83.7	23.6
	Dhar	94.8	88.8	85.7	69.4	82.3	68.1	29.6	84.0	25.4
	Indore	97.0	73.0	82.9	71.5	86.5	95.2	76.1	83.3	17.6
	Khargone (West Nimar)	91.0	71.7	86.6	59.4	81.3	73.9	38.8	91.4	25.4
	Barwani	77.3	55.7	62.3	57.8	63.0	52.0	26.3	85.6	38.0
	Rajgarh	93.3	67.2	71.2	61.9	68.7	91.0	35.9	78.9	29.4
	Vidisha	75.8	58.5	65.4	40.2	75.6	60.3	16.9	57.8	45.3
	Bhopal	94.5	87.4	89.5	71.2	74.4	94.3	56.6	77.7	21.1
	Sehore	98.9	78.0	86.1	74.2	94.4	81.2	40.9	78.1	22.8
	Raisen	96.6	90.3	91.4	43.8	81.1	86.4	52.1	88.5	21.9
	Betul	97.5	90.8	96.2	64.0	67.7	76.1	39.8	88.9	23.4
	Harda	95.4	73.6	88.6	76.8	91.6	78.5	39.9	85.3	22.1

Hoshangabad	97.3	73.9	79.0	79.7	82.1	82.8	46.3	84.4	22.3
Katni	97.3	83.7	85.4	60.1	48.5	75.5	32.7	83.5	30.1
Jabalpur	98.0	88.6	90.0	68.2	89.0	85.4	57.5	86.4	18.1
Narsimhapur	93.7	84.4	75.6	72.1	63.0	76.2	34.3	86.1	26.6
Dindori	97.3	72.6	85.3	61.8	67.5	54.1	23.5	89.4	31.3
Mandla	100.0	80.7	90.5	69.2	67.8	60.1	44.7	89.4	25.4
Chhindwara	96.0	87.1	92.4	56.4	75.8	79.2	41.9	87.8	23.7
Seoni	98.0	93.2	89.8	80.8	22.9	82.6	41.9	91.9	25.1
Balaghat	92.4	92.7	85.9	62.8	86.9	83.8	37.7	86.0	21.9
Guna	94.9	73.4	80.7	52.5	78.3	90.4	31.9	85.0	27.0
Ashoknagar	80.9	59.1	59.6	77.8	64.7	69.7	38.5	81.4	32.2
Shahdol	85.4	74.5	80.7	68.1	70.8	71.2	21.9	79.4	31.5
Anuppur	95.4	80.7	90.8	64.6	62.2	73.5	35.0	79.8	28.9
Sidhi	77.1	59.7	63.8	81.1	57.7	57.4	11.1	63.7	41.9
Singrauli	87.1	69.4	73.5	31.5	56.4	44.8	20.9	71.3	44.3
Jhabua	78.4	46.2	71.7	55.2	72.3	68.8	20.8	42.8	47.0
Alirajpur	82.9	37.0	60.0	81.1	74.2	49.9	21.0	74.4	39.6
Khandwa (East Nimar)	98.9	74.9	89.9	64.4	74.8	82.6	48.5	90.1	22.5
Burhanpur	90.8	67.9	70.0	64.1	63.1	76.9	40.8	84.7	29.7
Kachchh	95.6	72.4	78.4	35.9	81.0	83.0	62.2	68.4	40.3
Banaskantha	72.6	53.8	63.2	30.3	100.0	79.8	59.1	67.9	34.2
Patan	72.5	46.7	54.9	60.3	69.3	94.0	67.5	70.1	32.3
Mahesana	100.0	88.0	83.4	67.8	0.0	86.1	81.3	76.5	29.0
Sabarkantha	91.4	70.5	76.1	55.5	85.4	82.4	66.4	73.6	26.1
Gandhinagar	94.6	79.1	81.2	41.1	100.0	94.1	88.5	81.3	18.3

**Gujarat**	Ahmadabad	90.4	82.9	76.4	68.7	100.0	94.6	95.9	81.8	13.8
	Surendranagar	76.0	64.0	48.2	73.6	100.0	85.6	52.0	77.2	26.0
	Rajkot	94.8	78.6	89.9	44.8	100.0	81.0	86.8	69.8	22.1
	Jamnagar	98.2	88.4	87.9	61.9	100.0	91.2	80.2	68.2	18.6
	Porbandar	91.4	91.4	89.9	44.4	100.0	96.2	56.9	66.9	23.4
	Junagadh	91.2	84.7	76.1	81.8	70.0	93.9	76.2	77.8	19.3
	Amreli	94.4	78.9	82.7	38.5	0.0	83.3	40.8	70.1	41.2
	Bhavnagar	80.1	72.1	64.0	38.0	76.3	93.9	47.2	63.5	34.2
	Anand	99.1	81.3	91.0	45.6	54.5	93.8	78.5	79.0	24.1
	Kheda	80.2	55.3	63.6	50.4	69.1	85.8	49.2	60.0	37.3
	Panchmahal	63.7	47.4	58.6	38.6	74.3	78.4	50.8	57.5	41.8
	Dohad	65.3	42.9	52.0	73.0	92.4	81.8	38.9	64.4	35.4
	Vadodara	95.8	74.0	88.8	54.7	89.2	78.0	70.9	72.2	24.6
	Narmada	95.5	86.3	81.9	56.3	65.5	74.7	70.3	81.6	24.4
	Bharuch	98.3	92.0	77.9	31.9	50.1	82.9	86.4	86.5	24.4
	The Dangs	86.1	72.0	72.4	57.1	39.1	53.7	47.6	73.0	38.2
	Navsari	100.0	90.2	91.5	53.9	100.0	95.5	91.9	87.8	12.2
	Valsad	88.0	80.2	81.7	42.4	65.5	77.8	55.0	68.2	32.2
	Surat	90.4	72.0	72.8	61.4	74.8	95.3	71.6	72.4	24.8
	Tapi	97.8	83.2	91.6	65.4	79.9	86.0	74.5	81.6	19.2
**Daman and Diu**	Diu	95.1	93.2	90.4	82.3	91.2	87.7	79.6	67.1	17.4
	Daman	81.7	69.4	76.4	78.2	100.0	74.0	58.2	61.8	27.2
**Dadra and Nagar Haveli**	Dadra & Nagar Haveli	88.9	75.1	81.7	91.4	87.0	89.5	75.6	66.6	20.4
	Nandurbar	73.7	48.7	70.1	59.0	81.8	62.4	52.5	83.9	32.0
	Dhule	95.3	69.9	85.1	79.7	100.0	82.5	62.5	88.9	17.2

Maharashtra	Jalgaon	85.0	58.4	83.1	73.0	100.0	84.0	64.6	85.7	20.6
	Buldana	91.1	75.8	76.5	89.9	82.6	87.9	74.5	92.6	15.0
	Akola	90.4	72.0	83.5	82.4	94.3	94.4	80.4	89.1	13.9
	Washim	93.1	89.4	79.7	87.4	100.0	82.2	67.5	92.4	12.8
	Amravati	91.0	78.1	89.7	76.6	80.5	88.8	75.7	91.9	15.8
	Wardha	95.5	81.4	80.4	65.4	79.4	95.5	77.3	94.5	15.5
	Nagpur	96.1	89.5	92.1	92.5	88.6	98.1	81.1	89.7	9.6
	Bhandara	100.0	93.2	87.7	94.5	100.0	100.0	83.4	93.4	6.0
	Gondiya	91.3	87.6	85.4	52.0	59.8	93.0	76.0	88.5	20.8
	Gadchiroli	96.9	93.7	87.7	89.1	100.0	94.2	76.6	93.3	8.4
	Chandrapur	96.8	88.6	93.0	100.0	100.0	93.1	79.9	90.5	7.8
	Yavatmal	96.3	88.2	88.4	84.2	85.6	86.7	71.4	90.4	13.9
	Nanded	88.2	76.3	82.9	69.0	88.2	89.2	70.5	85.9	18.7
	Hingoli	96.0	75.2	87.2	57.4	100.0	83.5	63.7	89.9	18.6
	Parbhani	97.3	80.6	87.7	80.9	86.9	89.3	79.3	90.6	13.7
	Jalna	95.1	81.2	89.0	73.5	68.5	91.4	64.2	88.5	19.0
	Aurangabad	94.8	85.1	84.6	49.9	82.6	95.6	70.6	88.1	18.8
	Nashik	91.4	82.7	86.0	87.3	100.0	88.1	58.6	87.1	15.1
	Thane	84.5	62.8	72.3	90.0	91.5	90.7	70.8	87.7	17.5
	Mumbai Suburban	75.0	65.7	69.2	86.3	87.5	97.9	82.0	84.5	17.4
	Mumbai	87.6	50.6	73.9	66.7	68.4	93.4	80.7	82.3	24.4
	Raigarh	94.9	84.4	92.9	84.2	80.6	93.7	68.9	88.2	14.8
	Pune	98.1	92.8	98.1	88.9	81.0	95.5	84.5	89.2	10.1
	Ahmadnagar	94.0	63.2	76.7	59.5	93.6	96.2	63.1	80.4	22.3
	Bid	88.5	75.7	79.6	77.3	100.0	94.7	72.9	89.1	14.6

	Latur	92.8	79.6	87.2	66.9	80.2	88.4	74.9	88.2	18.0
	Osmanabad	88.4	77.3	84.9	71.5	73.4	87.0	74.8	90.7	18.5
	Solapur	96.2	83.3	86.6	76.4	57.3	92.3	73.8	89.0	18.5
	Satara	89.8	70.9	83.8	63.6	77.9	90.0	68.9	84.6	21.6
	Ratnagiri	92.4	78.5	92.4	71.3	86.7	81.3	72.0	72.9	21.5
	Sindhudurg	92.1	84.9	91.6	100.0	91.1	97.3	78.9	82.7	11.3
	Kolhapur	84.9	70.2	77.2	67.6	83.5	83.8	69.1	83.2	22.3
	Sangli	88.5	68.3	85.0	77.6	89.0	95.2	65.5	83.9	18.7
**Telangana**	Adilabad	88.7	81.5	84.3	74.4	76.1	78.0	68.3	87.4	20.1
	Nizamabad	100.0	89.7	89.5	45.0	76.8	75.2	70.1	86.7	21.9
	Karimnagar	100.0	92.0	94.9	76.8	96.4	97.2	72.8	79.6	13.5
	Medak	96.8	89.7	93.7	49.9	43.8	87.0	68.1	89.9	23.3
	Hyderabad	100.0	89.7	87.9	76.2	93.6	99.9	85.5	84.1	11.6
	Rangareddy	94.9	93.0	91.3	65.3	62.0	91.6	77.3	92.4	16.6
	Mahbubnagar	100.0	85.9	85.3	73.3	76.8	86.0	60.2	91.3	17.8
	Nalgonda	95.9	78.9	86.5	56.2	74.7	93.0	72.5	95.3	17.8
	Warangal	97.8	84.1	97.8	61.8	72.4	95.9	84.7	86.8	16.2
	Khammam	100.0	83.8	90.3	41.7	77.8	96.5	79.2	93.9	17.2
**Andhra Pradesh**	Srikakulam	100.0	88.5	88.9	63.7	100.0	97.6	72.7	91.2	12.6
	Vizianagaram	100.0	88.1	92.6	50.7	71.4	95.4	70.4	95.7	17.1
	Visakhapatnam	100.0	93.3	89.3	53.1	100.0	82.3	81.8	92.6	13.7
	East Godavari	96.7	77.2	82.4	62.3	83.1	91.7	77.2	95.6	16.0
	West Godavari	97.0	97.0	94.2	54.0	74.4	93.3	71.5	96.3	15.2
	Krishna	100.0	97.2	100.0	42.8	74.2	95.1	88.2	94.3	14.2
	Guntur	94.3	86.2	87.7	37.0	45.8	95.8	68.3	92.4	23.9

	Prakasam	97.7	89.0	93.4	56.9	100.0	95.7	73.7	93.8	12.7
	Sri Potti Sriramulu N	86.3	73.4	66.9	66.6	89.7	98.4	85.8	95.9	25.9
	Y.S.R.	100.0	94.7	93.7	43.5	79.4	95.9	83.5	91.6	15.4
	Kurnool	95.5	90.1	89.7	40.4	100.0	86.2	71.7	93.4	16.5
	Anantapur	98.3	94.7	89.2	76.1	100.0	89.1	77.5	92.6	10.5
	Chittoor	98.0	87.6	91.1	84.8	100.0	87.9	70.4	93.0	11.1
**Karnataka**	Belgaum	100.0	84.8	84.3	63.8	87.2	98.9	78.5	85.5	15.5
	Bagalkot	97.3	95.6	93.8	52.4	100.0	98.3	79.7	87.5	12.9
	Bijapur	84.8	74.8	72.2	92.4	94.6	93.6	64.3	89.3	15.4
	Bidar	92.8	81.3	81.2	90.8	89.8	98.9	69.1	89.0	13.1
	Raichur	94.2	77.5	84.7	52.4	0.0	89.2	65.4	85.6	31.8
	Koppal	97.1	88.4	91.3	92.5	100.0	91.1	60.5	81.6	13.8
	Gadag	92.4	75.7	69.4	95.7	87.5	92.6	78.1	90.5	13.6
	Dharwad	97.5	83.2	83.4	75.7	46.0	96.0	76.8	87.1	19.7
	Uttara Kannada	97.9	87.8	89.7	73.7	63.5	84.6	79.6	75.2	20.8
	Haveri	98.0	80.0	92.8	61.9	51.4	91.0	68.8	83.8	23.0
	Bellary	98.5	85.8	90.9	76.9	86.8	93.1	80.1	86.0	13.9
	Chitradurga	95.9	76.3	68.0	100.0	84.8	95.1	67.2	91.7	13.9
	Davanagere	100.0	89.4	96.1	63.4	90.6	98.7	88.1	88.0	12.0
	Shimoga	92.4	78.4	63.7	66.4	100.0	91.7	73.8	74.3	20.4
	Udupi	97.6	77.2	88.1	48.3	65.0	96.5	84.2	68.5	24.9
	Chikmagalur	100.0	68.7	81.4	70.5	78.4	83.2	60.9	85.0	22.2
	Tumkur	95.1	79.7	79.5	45.1	100.0	99.0	70.6	85.7	18.4
	Bangalore	80.2	67.8	76.9	54.6	61.0	90.0	48.1	77.2	30.7
	Mandya	100.0	75.7	88.6	57.9	51.4	88.0	83.6	88.9	21.4

	Hassan	97.8	84.6	84.1	68.8	91.0	96.5	86.4	87.9	13.3
	Dakshina Kannada	91.8	86.3	91.8	16.0	82.0	89.3	66.8	57.2	31.7
	Kodagu	94.9	88.6	76.3	69.8	79.0	95.5	82.6	76.9	18.1
	Mysore	84.3	62.9	71.5	54.0	91.6	94.7	65.9	84.9	22.9
	Chamarajanagar	97.6	75.5	82.4	65.9	100.0	95.8	76.8	86.7	15.3
	Gulbarga	100.0	82.7	86.8	65.2	70.0	96.8	84.6	89.4	16.1
	Yadgir	93.6	80.9	83.3	47.4	73.9	89.9	63.6	85.1	23.2
	Kolar	96.9	91.3	92.5	64.2	29.0	97.9	76.6	89.9	20.8
	Chikkaballapura	91.5	85.6	73.3	69.1	100.0	89.4	92.7	89.0	12.8
	Bangalore Rural	100.0	85.5	83.1	61.9	56.1	99.6	75.6	84.6	20.1
	Ramanagara	100.0	74.9	95.3	59.9	78.4	93.2	74.0	85.9	18.8
**Goa**	North Goa	100.0	96.6	93.8	73.3	100.0	99.0	93.9	53.5	16.7
	South Goa	100.0	92.0	98.9	100.0	92.0	95.4	81.5	68.5	12.8
**Lakshadweep**	Lakshadweep	100.0	95.1	93.7	79.5	78.9	100.0	82.3	64.0	17.4
**Kerala**	Kasaragod	98.0	93.5	93.5	93.5	100.0	100.0	90.9	74.0	9.8
	Kannur	98.5	94.3	97.0	88.6	100.0	100.0	93.2	78.3	8.7
	Wayanad	100.0	83.6	83.5	100.0	71.5	99.6	91.7	83.8	11.8
	Kozhikode	100.0	86.9	84.7	100.0	67.0	100.0	94.4	81.5	12.0
	Malappuram	95.1	80.8	78.8	100.0	100.0	100.0	93.2	71.5	12.0
	Palakkad	97.4	89.9	90.1	71.9	100.0	100.0	90.4	83.9	10.8
	Thrissur	100.0	97.4	93.6	100.0	100.0	100.0	83.1	86.9	6.1
	Ernakulam	100.0	90.4	81.6	87.2	51.9	100.0	94.7	83.7	14.7
	Idukki	100.0	96.3	92.2	73.9	100.0	100.0	89.9	89.0	8.2
	Kottayam	100.0	100.0	95.2	100.0	100.0	100.0	85.4	82.8	6.4
	Alappuzha	90.8	90.8	90.8	100.0	100.0	99.0	79.6	72.0	12.0

	Pathanamthitta	92.5	89.1	92.6	75.5	92.5	100.0	90.0	78.5	12.9
	Kollam	100.0	98.5	93.1	82.0	100.0	100.0	88.7	78.6	9.6
	Thiruvananthapuram	100.0	87.7	96.2	77.0	100.0	100.0	89.1	77.5	11.7
**Tamil Nadu**	Thiruvallur	97.7	85.6	87.9	90.3	85.8	99.5	91.6	87.5	9.9
	Chennai	99.2	97.6	89.4	68.8	67.1	99.1	78.3	91.1	14.1
	Kancheepuram	92.2	86.7	70.8	63.2	73.2	100.0	73.6	87.6	18.3
	Vellore	93.8	92.3	83.6	76.4	95.9	100.0	92.5	86.8	10.1
	Tiruvannamalai	94.6	84.0	81.0	70.6	92.8	98.4	83.5	89.7	12.9
	Viluppuram	95.5	88.8	81.8	70.6	83.9	99.6	79.5	88.3	14.1
	Salem	97.2	84.2	87.2	82.3	100.0	98.0	78.7	90.3	10.5
	Namakkal	97.7	85.6	81.7	75.0	89.4	100.0	81.6	86.8	13.2
	Erode	100.0	97.0	89.1	78.0	90.8	100.0	77.2	89.3	10.5
	The Nilgiris	100.0	98.4	90.3	59.5	89.1	99.8	88.8	88.4	11.6
	Dindigul	96.7	92.2	91.6	80.0	90.6	99.8	88.8	89.6	9.4
	Karur	100.0	97.6	95.5	43.7	77.9	100.0	82.9	89.5	15.1
	Tiruchirappalli	90.7	81.0	88.7	78.3	92.6	99.1	85.8	75.7	15.3
	Perambalur	94.3	86.6	84.1	76.1	87.9	100.0	77.7	77.4	16.0
	Ariyalur	92.9	81.7	80.6	72.5	86.5	98.9	79.1	80.1	16.8
	Cuddalore	94.4	85.1	80.9	60.3	94.2	99.5	85.5	81.3	15.6
	Nagapattinam	72.6	54.9	56.6	69.4	74.4	99.2	68.3	87.2	24.4
	Thiruvarur	98.8	82.9	97.0	44.5	92.2	99.6	83.4	80.7	17.3
	Thanjavur	96.6	93.4	93.0	58.4	77.8	99.2	90.2	79.7	15.9
	Pudukkottai	90.3	73.0	76.9	62.6	85.0	99.3	76.9	77.8	20.5
	Sivaganga	98.0	82.1	90.1	83.8	93.5	99.0	86.0	76.3	13.6
	Madurai	96.1	79.5	77.3	70.2	88.0	100.0	69.5	75.8	19.3

	Theni	93.4	77.0	78.5	65.0	83.3	99.2	75.9	82.8	18.5
	Virudhunagar	85.9	66.6	80.5	73.2	73.0	100.0	65.9	71.3	24.4
	Ramanathapuram	89.7	77.3	82.7	50.0	92.8	99.1	65.9	60.7	25.9
	Thoothukkudi	86.5	64.7	78.1	56.4	82.7	96.9	64.8	71.2	26.2
	Tirunelveli	91.6	72.1	91.7	50.3	85.7	97.8	71.0	70.0	24.0
	Kanniyakumari	97.0	71.7	78.4	71.9	84.9	98.9	81.5	76.5	18.8
	Dharmapuri	89.5	79.9	81.0	64.4	82.7	99.7	86.3	87.1	16.0
	Krishnagiri	96.4	82.9	90.3	54.6	100.0	95.8	86.3	88.1	13.9
	Coimbatore	97.7	91.7	93.0	63.9	94.9	99.3	88.5	89.1	11.0
	Tiruppur	100.0	97.9	93.2	59.9	94.9	100.0	89.5	87.3	10.8
**Puducherry**	Yanam	98.1	96.2	89.9	68.4	100.0	99.6	76.3	91.1	10.4
	Puducherry	100.0	97.0	96.2	84.0	82.7	100.0	88.6	90.3	8.6
	Mahe	98.3	98.3	93.4	55.2	95.9	100.0	82.3	71.8	16.1
	Karaikal	100.0	92.0	93.7	66.9	79.4	100.0	87.2	80.1	14.7
**Andaman and Nicobar**	Nicobars	61.1	55.9	46.9	100.0	100.0	98.0	64.9	69.0	23.7
	North & Middle Andama	95.4	95.4	90.9	100.0	66.5	95.5	95.2	84.8	10.6
	South Andaman	86.6	83.6	73.1	78.1	89.5	97.9	93.4	74.5	16.1
