# Supplementary material for: District level inequality in reproductive, maternal, neonatal and child health coverage in India
Source: BMC Public Health. 2020 Jan 14;20:58. doi: 10.1186/s12889-020-8151-9 (PMC6961337; doi:10.1186/s12889-020-8151-9)
Supplement: Supplementary file 2 — Additional file 2: Coverage of various RMNCH indicators in percentages, twenty lowest and highest districts across India, NFHS-4, 2015–16. [file 12889_2020_8151_MOESM2_ESM.docx]

Additional file 2: Coverage of various RMNCH indicators in percentages, twenty lowest and highest districts across India, NFHS-4, 2015–16.

| Family planning | | | |
| --- | --- | --- | --- |
| Lowest | Percentage | Highest | Percentage |
| Balrampur | 8.0 | Muktsar | 98.0 |
| Pashchim Champaran | 16.2 | Ambala | 97.8 |
| East Kameng | 19.9 | Fatehabad | 97.2 |
| Purba Champaran | 20.2 | Panchkula | 96.9 |
| Shrawasti | 22.6 | Tarn Taran | 96.3 |
| Saran | 25.9 | West Godavari | 96.3 |
| West Siang | 26.7 | Patiala | 96.2 |
| Bahraich | 27.2 | Bathinda | 96.0 |
| Gopalganj | 28.8 | Jind | 96.0 |
| Siwan | 29.4 | Sri Potti Sriramulu N | 95.9 |
| Papumpare | 29.6 | Amritsar | 95.8 |
| Ukhrul | 30.0 | Vizianagaram | 95.7 |
| Gonda | 30.5 | East Godavari | 95.6 |
| Lower Subansiri | 33.6 | Firozpur | 95.4 |
| Kishanganj | 33.7 | Nalgonda | 95.3 |
| Muzaffarpur | 33.7 | Birbhum | 95.1 |
| Mewat | 34.1 | Yamunanagar | 95.0 |
| Chandel | 34.7 | Panipat | 94.7 |
| Samastipur | 35.9 | Wardha | 94.5 |
| Tamenglong | 36.1 | Karnal | 94.5 |
| Maternal and newborn care | | | |
| Lowest | Percentage | Highest | Percentage |
| Mon | 9.0 | Ernakulam | 97.4 |
| Longleng | 9.7 | Kozhikode | 97.2 |
| Phek | 15.8 | Badgam | 97.1 |
| Zunheboto | 15.8 | Kannur | 96.6 |
| Kiphire | 16.0 | Malappuram | 96.6 |
| East Kameng | 18.3 | North Goa | 96.5 |
| Tuensang | 19.5 | Vellore | 96.3 |
| Kurung Kumey | 19.8 | Pulwama | 96.1 |
| Bahraich | 20.6 | South Andaman | 95.6 |
| Tawang | 23.5 | Wayanad | 95.6 |
| Balrampur | 25.4 | Thiruvallur | 95.5 |
| Mewat | 26.1 | Kasaragod | 95.5 |
| Pashchimi Singhbhum | 26.6 | North & Middle Andama | 95.4 |
| Shrawasti | 28.9 | Srinagar | 95.3 |
| Siddharth Nagar | 29.5 | Ganderbal | 95.3 |
| Upper Subansiri | 31.1 | Ahmadabad | 95.3 |
| Sitamarhi | 31.4 | Palakkad | 95.2 |
| Tirap | 32.2 | Pathanamthitta | 95.0 |
| Singrauli | 32.9 | Idukki | 95.0 |
| Katihar | 33.0 | Tiruppur | 94.7 |
| Immunisation | | | |
| Lowest | Percentage | Highest | Percentage |
| East Kameng | 22.4 | Panchkula | 100.0 |
| Bahraich | 25.8 | Kapurthala | 100.0 |
| Balrampur | 30.5 | Nadia | 99.6 |
| Kurung Kumey | 33.6 | Bankura | 99.5 |
| Mon | 35.3 | Dhenkanal | 99.5 |
| Mewat | 36.3 | Faridkot | 99.5 |
| Longleng | 37.4 | Ambala | 99.4 |
| Shrawasti | 37.5 | North District | 99.0 |
| Dhubri | 40.7 | Hugli | 98.8 |
| Upper Subansiri | 41.1 | Patiala | 98.8 |
| Tawang | 47.5 | Kottayam | 98.8 |
| Wokha | 49.9 | Rajnandgaon | 98.7 |
| West Kameng | 50.7 | South District | 98.7 |
| Dohad | 50.8 | Tarn Taran | 98.7 |
| Zunheboto | 51.0 | Krishna | 98.6 |
| Kiphire | 51.2 | Moga | 98.5 |
| East Garo Hills | 53.5 | Durg | 98.4 |
| Karbi Anglong | 53.7 | Baudh | 98.3 |
| Alirajpur | 54.2 | Subarnapur | 98.3 |
| Palwal | 54.3 | Balangir | 98.2 |
| Treatment of sick children | | | |
| Lowest | Percentage | Highest | Percentage |
| South District | 10.0 | Malappuram | 100.0 |
| Zunheboto | 14.1 | Kathua | 100.0 |
| Kachchh | 18.0 | Kottayam | 100.0 |
| Amreli | 19.2 | Thrissur | 100.0 |
| Kokrajhar | 20.7 | West District | 100.0 |
| Mon | 22.4 | Panchkula | 100.0 |
| Tirap | 24.6 | Alappuzha | 100.0 |
| Raichur | 26.2 | Chandrapur | 100.0 |
| Phek | 27.3 | Nicobars | 100.0 |
| Tuensang | 28.8 | Bhandara | 97.3 |
| Chirang | 29.1 | Kasaragod | 96.8 |
| Lower Subansiri | 30.9 | Birbhum | 96.6 |
| Sri Potti Sriramulu N | 33.3 | Panipat | 96.3 |
| Mahesana | 33.9 | Haora | 96.2 |
| Jalaun | 34.1 | Koppal | 96.2 |
| Karbi Anglong | 34.4 | South Goa | 96.0 |
| Tawang | 34.6 | Kurukshetra | 95.9 |
| Tamenglong | 34.8 | Sindhudurg | 95.5 |
| Changlang | 36.0 | Kapurthala | 94.8 |
| Upper Siang | 36.7 | Faridkot | 94.6 |
| Coverage Gap Index | | | |
| Lowest | Percentage | Highest | Percentage |
| Panchkula | 3.4 | Mon | 74.1 |
| Faridkot | 5.2 | Bahraich | 69.9 |
| Kathua | 5.9 | East Kameng | 68.7 |
| Bhandara | 6.0 | Balrampur | 68.4 |
| Thrissur | 6.1 | Shrawasti | 65.5 |
| Kottayam | 6.4 | Zunheboto | 63.5 |
| Kapurthala | 7.1 | Kurung Kumey | 63.2 |
| Birbhum | 7.4 | Tawang | 62.8 |
| Yamunanagar | 7.6 | Phek | 61.1 |
| Chandrapur | 7.8 | Tuensang | 61.0 |
| Srinagar | 8.0 | Mewat | 60.0 |
| Muktsar | 8.1 | Longleng | 58.6 |
| Idukki | 8.2 | Lower Subansiri | 58.4 |
| Gadchiroli | 8.4 | Tamenglong | 56.7 |
| Nadia | 8.4 | Kiphire | 56.7 |
| West District | 8.5 | Ukhrul | 56.6 |
| Kurukshetra | 8.5 | Gonda | 54.8 |
| Puducherry | 8.6 | Pashchim Champaran | 53.9 |
| Kannur | 8.7 | Purba Champaran | 53.4 |
| Ganderbal | 9.3 | West Siang | 53.1 |
