# Supplementary material for: District level inequality in reproductive, maternal, neonatal and child health coverage in India
Source: BMC Public Health. 2020 Jan 14;20:58. doi: 10.1186/s12889-020-8151-9 (PMC6961337; doi:10.1186/s12889-020-8151-9)
Supplement: Supplementary file 3 — Additional file 3: Percentage coverage of asset, water, sanitation, female literacy, urbanization and the socio-demographic development index (SDI) by districts of India. [file 12889_2020_8151_MOESM3_ESM.pdf]

S2 Table: Percentage coverage of Asset, water, sanitation, female literacy, urbanization and socio demographic development index (SDI) by districts of India.

States	Districts	Assert Index	Water	Sanitation	Electricity	Female Literacy	Percent Urban	SDI
Jammu and Kashmir	Kupwara	33.7	89.3	38.4	93.3	68.9	21.6	57.5
	Badgam	38.8	97.5	50.0	99.0	58.8	18.4	60.4
	Leh	41.6	76.9	16.7	99.5	71.2	36.5	57.1
	Kargil	32.1	87.6	16.5	97.3	68.4	21.9	53.9
	Punch	44.0	75.0	54.7	96.8	69.4	6.6	57.8
	Rajouri	44.4	66.7	39.6	94.3	71.3	7.4	53.9
	Kathua	54.9	84.9	43.5	99.6	79.7	14.7	62.9
	Baramula	40.9	92.6	62.4	99.8	69.2	22.1	64.5
	Bandipore	38.0	91.5	37.2	95.1	65.6	25.9	58.9
	Srinagar	56.9	97.3	66.6	100.0	74.2	98.9	82.3
	Ganderbal	40.7	95.9	55.8	99.7	63.3	25.7	63.5
	Pulwama	44.0	95.8	66.0	98.7	71.5	5.8	63.6
	Shupiyan	38.7	94.5	57.2	96.7	67.7	7.0	60.3
	Anantnag	36.7	96.2	54.6	96.7	68.5	36.2	64.8
	Kulgam	34.3	98.1	50.5	97.0	64.5	20.2	60.8
	Doda	32.4	86.3	45.5	94.4	54.8	6.5	53.3
	Ramban	31.6	81.7	41.1	89.1	45.5	5.2	49.0
	Kishtwar	36.0	87.1	39.0	87.8	52.0	6.8	51.4
	Udhampur	47.8	64.5	34.6	97.6	71.8	16.9	55.5
	Reasi	45.4	71.8	44.2	90.6	66.6	8.5	54.5
	Jammu	64.6	97.3	64.3	99.9	86.0	51.7	77.3
	Samba	57.4	95.4	44.9	99.3	84.9	5.8	64.6

**Himachal Pradesh**	Chamba	46.4	89.4	75.7	98.7	79.6	7.6	66.2
	Kangra	57.8	97.8	67.1	100.0	95.5	5.6	70.6
	Lahul And Spiti	37.6	97.1	42.4	97.8	83.2	0.0	59.7
	Kullu	42.8	94.7	65.2	99.5	84.1	9.3	65.9
	Mandi	47.7	96.0	71.6	99.5	88.4	6.4	68.3
	Hamirpur	56.7	96.8	81.0	99.5	98.2	7.1	73.2
	Una	57.7	98.8	69.3	99.8	95.4	7.7	71.4
	Bilaspur	54.3	82.8	70.3	99.9	94.5	6.3	68.0
	Solan	56.0	94.3	69.1	98.8	88.8	25.7	72.1
	Sirmaur	51.6	90.8	69.3	98.8	85.0	14.3	68.3
	Shimla	48.9	97.8	74.0	99.7	86.7	27.6	72.4
	Kinnaur	43.3	92.7	67.1	99.1	89.7	0.0	65.3
**Punjab**	Gurdaspur	67.7	99.7	74.6	99.6	89.1	32.1	77.1
	Kapurthala	68.0	100.0	81.7	97.0	87.9	37.4	78.7
	Jalandhar	72.1	99.9	89.2	99.8	90.9	54.5	84.4
	Hoshiarpur	68.2	100.0	76.2	99.8	91.5	22.1	76.3
	Sangrur	67.7	100.0	81.4	99.7	89.1	21.6	76.6
	Fatehgarh Sahib	68.1	100.0	83.0	100.0	90.2	33.1	79.0
	Ludhiana	65.3	100.0	80.8	99.9	86.3	63.1	82.6
	Moga	66.4	100.0	86.9	99.4	79.0	25.6	76.2
	Firozpur	62.3	97.6	77.1	99.7	71.1	28.9	72.8
	Muktsar	65.7	99.7	79.6	99.6	78.4	36.4	76.6
	Faridkot	66.1	98.6	83.7	98.9	76.8	25.7	75.0
	Bathinda	66.9	98.6	85.0	99.5	78.4	46.6	79.1
	Mansa	61.3	99.1	74.9	99.8	71.6	23.5	71.7

	Patiala	68.6	99.5	87.5	99.9	86.0	48.4	81.6
	Amritsar	66.0	100.0	78.3	99.4	80.4	55.2	79.9
	Tarn Taran	66.8	100.0	77.9	99.6	76.6	16.6	72.9
	Rupnagar	68.6	99.8	80.9	100.0	90.4	30.3	78.3
	Sahibzada Ajit Singh	65.3	99.9	79.4	99.5	87.4	58.6	81.7
	Shahid Bhagat Singh N	66.4	99.3	87.7	99.5	76.3	35.0	77.4
	Barnala	66.9	100.0	85.5	99.9	79.6	33.7	77.6
**Chandigarh**	Chandigarh	66.3	100.0	82.9	99.6	86.3	97.0	88.7
**Uttarakhand**	Uttarkashi	38.5	75.1	48.5	93.1	72.2	8.4	56.0
	Chamoli	38.7	93.2	62.4	95.5	86.9	17.2	65.6
	Rudraprayag	40.1	86.5	67.6	98.8	85.4	7.0	64.2
	Tehri Garhwal	44.3	77.4	65.8	98.1	78.4	18.9	63.8
	Dehradun	60.0	99.8	75.6	99.4	84.3	64.7	80.7
	Garhwal	45.7	88.1	66.2	98.1	88.8	15.1	67.0
	Pithoragarh	37.7	83.9	62.7	97.3	82.9	17.2	63.6
	Bageshwar	38.6	83.1	67.3	97.5	84.0	4.4	62.5
	Almora	37.0	84.0	64.8	96.4	83.4	9.4	62.5
	Champawat	40.3	89.8	59.5	91.7	78.3	15.2	62.5
	Nainital	53.2	96.5	73.0	98.6	87.1	43.4	75.3
	Udham Singh Nagar	51.8	98.4	56.1	96.1	69.1	40.0	68.6
	Hardwar	53.1	99.9	56.9	97.8	69.5	45.3	70.4
	Panchkula	71.2	99.5	86.9	99.6	87.7	64.9	85.0
	Ambala	70.1	99.8	89.9	99.8	90.8	46.3	82.8
	Yamunanagar	66.0	100.0	80.1	99.9	86.4	40.6	78.8
	Kurukshetra	66.4	100.0	86.0	99.9	81.3	31.4	77.5

	Kaithal	64.5	99.6	78.0	99.9	74.4	26.4	73.8
	Karnal	65.8	100.0	87.5	99.5	80.8	33.1	77.8
	Panipat	67.3	99.9	91.0	100.0	79.6	50.3	81.3
	Sonipat	64.8	99.6	79.9	99.7	82.7	35.1	77.0
	Jind	61.9	92.4	84.6	99.5	77.5	22.3	73.0
	Fatehabad	63.9	99.8	87.5	99.8	73.3	21.8	74.3
**Haryana**	Sirsa	62.5	99.8	79.4	98.9	71.4	28.7	73.5
	Hisar	61.9	97.2	84.4	99.0	74.2	33.3	75.0
	Bhiwani	61.4	93.1	82.6	99.2	77.1	20.3	72.3
	Rohtak	60.9	99.1	76.6	99.1	80.6	43.3	76.6
	Jhajjar	61.1	98.7	86.4	99.0	86.0	31.2	77.1
	Mahendragarh	59.9	99.1	73.8	98.6	79.7	15.9	71.2
	Rewari	57.6	99.4	68.9	98.6	79.9	33.2	72.9
	Gurgaon	55.3	99.6	66.3	99.8	77.5	79.6	79.7
	Mewat	41.9	94.5	46.7	88.5	40.1	19.8	55.3
	Faridabad	56.1	99.2	78.0	99.2	74.9	86.8	82.4
	Palwal	51.3	96.1	66.3	93.2	63.0	28.2	66.4
	North West	55.4	99.7	65.9	99.5	79.1	98.4	83.0
	North	56.5	100.0	77.8	99.4	85.8	98.4	86.3
	North East	58.9	99.5	75.5	100.0	84.2	100.0	86.4
	East	59.9	99.9	80.8	99.8	86.7	100.0	87.9
**Delhi**	New Delhi	60.0	100.0	67.7	99.6	85.1	100.0	85.4
	Central	59.5	100.0	74.4	99.6	89.0	100.0	87.1
	West	64.5	100.0	87.8	100.0	87.8	100.0	90.0
	South West	58.1	97.9	64.6	99.9	79.9	97.7	83.0

	South	56.6	100.0	73.6	99.6	84.5	100.0	85.7
	Ganganagar	54.0	95.3	73.4	94.1	70.3	28.1	69.2
	Hanumangarh	51.0	98.7	55.0	94.8	66.3	20.7	64.4
	Bikaner	47.6	98.9	64.8	91.5	62.3	38.1	67.2
	Churu	42.2	92.5	73.2	95.3	59.4	27.4	65.0
	Jhunjhunun	50.9	97.7	66.0	96.2	69.5	25.3	67.6
	Alwar	49.8	98.7	40.3	96.9	62.2	19.3	61.2
	Bharatpur	41.2	91.7	34.3	90.7	56.8	22.4	56.2
	Dhaulpur	37.5	94.0	31.4	91.4	61.2	22.7	56.4
	Karauli	34.1	87.4	21.1	94.3	55.2	15.0	51.2
	Sawai Madhopur	36.8	88.1	34.6	86.4	48.3	19.0	52.2
	Dausa	39.7	96.8	36.1	90.4	59.6	13.0	55.9
	Jaipur	55.1	98.8	58.1	98.0	70.7	53.5	72.4
	Sikar	52.8	99.1	61.2	96.0	66.0	26.2	66.9
	Nagaur	43.2	90.7	53.8	90.6	53.5	20.9	58.8
	Jodhpur	48.1	98.2	52.6	91.3	58.5	37.0	64.3
	Jaisalmer	33.9	93.7	36.8	76.4	41.4	12.1	49.1
**Rajasthan**	Barmer	27.0	93.5	20.2	65.6	41.9	6.7	42.5
	Jalor	33.6	93.5	39.3	82.8	45.5	8.7	50.6
	Sirohi	37.5	94.0	42.0	87.2	47.4	22.2	55.1
	Pali	43.3	92.5	50.9	95.1	62.5	21.0	60.9
	Ajmer	52.4	94.6	69.4	98.4	66.2	41.3	70.4
	Tonk	40.6	91.0	32.6	96.7	57.9	22.6	56.9
	Bundi	39.4	93.6	26.6	91.1	55.1	20.2	54.3
	Bhilwara	43.5	91.3	32.7	96.0	51.5	23.9	56.5

Rajsamand	38.7	84.6	32.3	95.2	60.2	14.2	54.2
Dungarpur	32.9	92.4	32.9	78.7	52.9	6.0	49.3
Banswara	27.0	93.6	20.8	67.9	48.3	7.6	44.2
Chittaurgarh	40.7	94.4	32.0	95.3	53.1	18.1	55.6
Kota	52.4	98.3	50.5	98.1	74.9	62.9	72.9
Baran	37.3	92.5	31.4	91.7	60.6	20.5	55.7
Jhalawar	35.1	84.9	36.1	95.0	55.8	15.4	53.7
Udaipur	34.6	80.9	29.2	84.2	54.7	19.0	50.5
Pratapgarh	27.4	85.6	15.1	75.3	48.1	7.4	43.1
Saharanpur	47.2	99.9	50.9	93.4	67.8	32.2	65.2
Muzaffarnagar	49.5	100.0	54.5	93.4	66.6	30.4	65.7
Bijnor	44.1	100.0	60.3	79.6	66.7	23.4	62.3
Moradabad	43.6	100.0	51.6	79.0	53.2	35.8	60.5
Rampur	43.7	100.0	52.6	80.8	50.0	25.8	58.8
Jyotiba Phule Nagar	42.4	100.0	48.8	74.8	59.7	25.9	58.6
Meerut	57.2	100.0	67.1	95.9	70.8	69.1	76.7
Baghpat	53.8	100.0	64.7	92.0	71.9	26.3	68.1
Ghaziabad	54.9	100.0	64.8	97.4	76.1	75.4	78.1
Gautam Buddha Nagar	54.1	100.0	54.5	98.6	76.2	71.7	75.9
Bulandshahr	46.0	99.8	47.7	87.9	60.5	25.4	61.2
Aligarh	46.7	98.7	38.4	84.6	63.6	34.1	61.0
Mahamaya Nagar	45.2	100.0	30.5	86.3	71.5	22.4	59.3
Mathura	45.6	97.4	43.0	95.6	65.7	37.8	64.2
Agra	49.9	99.0	42.7	94.5	66.5	51.2	67.3
Firozabad	39.9	99.3	33.6	85.5	68.6	35.4	60.4

	Mainpuri	37.4	99.4	27.2	81.9	77.3	17.0	56.7
	Budaun	33.2	100.0	30.5	52.4	47.8	20.2	47.4
	Bareilly	43.2	100.0	54.3	69.6	51.9	40.0	59.8
	Pilibhit	35.8	100.0	36.8	51.7	58.0	17.6	50.0
	Shahjahanpur	35.2	100.0	33.4	53.2	56.8	20.9	49.9
	Kheri	27.7	99.4	22.5	41.4	53.4	11.0	42.6
	Sitapur	29.1	98.7	16.4	29.9	57.6	13.1	40.8
	Hardoi	33.3	99.6	28.3	42.2	58.1	16.6	46.3
	Unnao	33.6	97.8	28.3	45.0	66.9	14.4	47.7
	Lucknow	56.2	99.1	66.9	93.5	81.9	67.2	77.5
	Rae Bareli	33.6	98.5	18.4	71.6	68.2	8.1	49.7
	Farrukhabad	36.6	99.9	30.3	62.6	70.7	23.3	53.9
	Kannauj	32.3	99.8	22.0	70.7	66.0	16.5	51.2
	Etawah	39.5	99.2	28.5	91.3	78.5	24.1	60.2
	Auraiya	36.5	98.5	35.6	67.9	75.4	17.9	55.3
	Kanpur Dehat	31.1	98.9	28.4	45.5	73.4	9.4	47.8
	Kanpur Nagar	53.9	99.6	65.2	85.9	84.2	71.0	76.6
	Jalaun	40.5	98.1	48.1	89.0	73.0	29.7	63.1
	Jhansi	43.7	97.1	45.7	88.9	72.9	42.3	65.1
**Uttar Pradesh**	Lalitpur	31.5	94.2	19.7	78.0	59.1	20.2	50.4
	Hamirpur	36.5	99.4	45.0	73.1	69.4	20.0	57.2
	Mahoba	35.4	95.6	35.3	79.2	66.2	24.5	56.0
	Banda	32.9	97.8	27.5	67.3	66.1	17.8	51.6
	Chitrakoot	27.2	94.0	15.8	67.3	58.2	13.5	46.0
	Fatehpur	31.7	97.8	26.4	45.4	64.7	10.4	46.1

Pratapgarh	39.6	92.8	15.8	71.3	65.2	6.6	48.5
Kaushambi	29.9	96.9	18.7	49.0	47.4	10.2	42.0
Allahabad	45.5	92.3	32.7	78.5	66.5	30.6	57.7
Bara Banki	33.6	99.3	20.3	49.7	58.1	13.3	45.7
Faizabad	38.9	99.4	24.2	65.4	68.1	14.1	51.7
Ambedkar Nagar	38.0	99.9	19.8	72.6	73.4	12.2	52.6
Sultanpur	38.5	95.6	16.7	75.0	61.9	6.2	49.0
Bahraich	25.8	99.1	12.9	31.3	39.8	7.5	36.1
Shrawasti	24.4	98.0	10.4	25.6	34.6	3.7	32.8
Balrampur	30.6	98.5	13.7	35.8	41.1	8.2	38.0
Gonda	33.4	100.0	11.1	39.6	50.3	8.6	40.5
Siddharth Nagar	34.9	99.9	14.9	61.9	52.6	6.5	45.1
Basti	38.3	100.0	16.1	66.6	61.2	7.1	48.2
Sant Kabir Nagar	36.4	99.9	15.2	63.1	58.3	8.8	47.0
Mahrajganj	36.5	99.9	19.4	51.7	50.8	6.7	44.2
Gorakhpur	43.1	99.8	34.3	74.8	70.9	20.9	57.3
Kushinagar	36.1	99.5	22.1	48.3	59.5	7.1	45.4
Deoria	40.5	100.0	24.8	70.5	66.3	14.8	52.8
Azamgarh	40.9	100.0	24.7	82.9	75.0	12.5	56.0
Mau	38.7	100.0	26.6	82.9	68.6	25.7	57.1
Ballia	37.2	99.7	24.0	69.8	65.6	11.2	51.2
Jaunpur	40.6	96.0	24.1	78.3	69.0	7.7	52.6
Ghazipur	36.9	99.4	19.1	67.9	65.7	8.8	49.6
Chandauli	38.9	89.7	27.9	70.8	66.6	16.6	51.8
Varanasi	47.9	96.0	48.7	88.4	73.6	48.5	67.2

	Sant Ravidas Nagar (B	38.0	86.4	20.6	80.1	65.1	17.0	51.2
	Mirzapur	35.7	88.1	23.3	68.3	62.5	16.0	49.0
	Sonbhadra	36.8	88.1	23.7	52.4	58.6	18.5	46.3
	Etah	37.8	100.0	23.1	65.9	69.3	16.4	52.1
	Kanshiram Nagar	35.0	99.8	20.7	54.3	57.6	20.5	48.0
Bihar	Pashchim Champaran	25.8	96.3	21.1	46.1	45.8	14.8	41.7
	Purba Champaran	25.4	99.4	20.1	40.4	41.5	8.4	39.2
	Sheohar	23.4	99.6	21.0	59.7	44.6	3.9	42.0
	Sitamarhi	22.8	100.0	20.2	39.4	47.5	7.5	39.5
	Madhubani	26.1	99.6	18.9	53.7	41.9	3.9	40.7
	Supaul	23.3	100.0	15.4	60.9	38.2	4.9	40.4
	Araria	23.9	99.6	12.5	48.6	46.6	7.0	39.7
	Kishanganj	26.9	98.9	15.9	51.1	38.5	8.8	40.0
	Purnia	27.7	99.8	14.3	50.6	49.0	14.0	42.5
	Katihar	26.8	99.5	21.1	31.6	43.4	8.9	38.6
	Madhepura	24.4	100.0	15.0	53.3	35.2	5.1	38.8
	Saharsa	25.6	99.9	16.5	58.3	42.3	8.2	41.8
	Darbhanga	26.6	99.9	27.4	58.4	45.4	9.2	44.5
	Muzaffarpur	32.0	99.9	28.5	72.7	56.0	10.0	49.9
	Gopalganj	31.3	99.0	25.9	56.8	58.5	7.3	46.5
	Siwan	33.4	99.2	23.5	53.2	62.8	5.6	46.3
	Saran	30.6	98.4	25.4	59.0	58.8	9.8	47.0
	Vaishali	32.1	98.7	29.8	63.1	57.7	9.9	48.5
	Samastipur	25.4	98.9	19.1	39.9	51.8	4.8	40.0
	Begusarai	27.3	99.5	34.0	63.6	52.7	19.8	49.5

	Khagaria	26.1	99.8	30.7	54.2	47.3	5.3	43.9
	Bhagalpur	31.5	98.2	32.1	69.2	57.6	23.8	52.1
	Banka	25.8	93.0	14.3	47.2	45.9	3.8	38.3
	Munger	32.8	92.3	34.1	68.8	65.9	26.6	53.4
	Lakhisarai	29.3	94.1	36.7	79.3	54.9	16.7	51.8
	Sheikhpura	29.7	94.5	33.3	79.1	53.4	20.3	51.7
	Nalanda	31.3	98.1	31.0	69.3	50.6	18.6	49.8
	Patna	41.5	99.2	49.9	86.6	66.9	52.6	66.1
	Bhojpur	33.0	99.9	26.2	64.5	59.5	18.0	50.2
	Buxar	34.7	99.9	27.9	65.7	66.3	13.1	51.3
	Kaimur (Bhabua)	32.2	95.4	21.5	73.6	61.6	6.3	48.4
	Rohtas	34.1	99.4	25.7	81.1	67.4	13.8	53.6
	Aurangabad	32.2	98.8	27.1	59.7	61.2	10.1	48.2
	Gaya	28.2	96.8	26.4	71.2	51.6	14.7	48.1
	Nawada	27.4	98.8	28.8	56.7	51.4	13.0	46.0
	Jamui	25.8	78.9	14.8	62.0	46.9	7.8	39.4
	Jehanabad	29.9	99.4	29.2	68.8	58.9	13.4	49.9
	Arwal	26.1	99.1	21.3	47.1	57.6	7.7	43.1
**Sikkim**	North District	39.7	98.2	89.3	99.6	87.8	18.6	72.2
	West District	38.6	94.1	92.9	99.6	86.2	5.3	69.5
	South District	41.6	99.6	93.3	99.8	88.6	18.8	73.6
	East District	42.3	98.3	83.6	99.1	88.4	53.0	77.4
	Tawang	29.3	94.7	48.8	95.4	38.5	10.2	52.8
	West Kameng	36.1	95.8	58.0	98.1	63.3	19.1	61.7
	East Kameng	21.7	78.6	37.7	50.5	51.7	31.7	45.3

**Arunachal Pradesh**	Papumpare	47.2	91.7	67.8	98.9	78.8	60.8	74.2
	Upper Subansiri	28.7	86.2	59.7	88.3	58.8	18.5	56.7
	West Siang	41.8	90.8	73.9	94.4	77.0	29.7	67.9
	East Siang	42.1	98.3	71.7	96.8	81.5	35.4	71.0
	Upper Siang	32.5	92.5	56.2	91.8	74.7	18.0	60.9
	Changlang	38.3	70.2	56.2	78.3	70.0	11.2	54.0
	Tirap	35.1	68.3	54.8	97.9	76.0	21.0	58.9
	Lower Subansiri	38.0	97.8	73.0	98.5	65.9	13.6	64.5
	Kurung Kumey	25.5	92.4	46.7	91.2	47.7	2.5	51.0
	Dibang Valley	32.9	99.4	75.3	91.8	71.7	18.4	64.9
	Lower Dibang Valley	39.0	96.2	68.5	72.5	65.7	22.2	60.7
	Lohit	36.5	92.8	60.2	78.4	69.3	21.1	59.7
	Anjaw	26.0	87.2	45.1	90.4	62.9	4.5	52.7
**Nagaland**	Mon	28.7	72.8	80.9	85.4	72.9	17.3	59.6
	Mokokchung	48.4	95.9	91.1	99.4	97.4	30.9	77.2
	Zunheboto	34.3	91.5	81.0	99.4	89.9	20.1	69.4
	Wokha	39.6	75.9	73.2	97.8	93.5	33.5	68.9
	Dimapur	50.3	79.4	65.2	98.7	85.2	55.7	72.4
	Phek	33.0	96.5	82.5	98.4	80.1	13.6	67.3
	Tuensang	33.0	91.9	72.9	97.1	84.4	21.1	66.7
	Longleng	30.9	41.4	73.3	97.5	83.9	18.3	57.6
	Kiphire	33.3	87.4	80.5	98.2	81.0	23.9	67.4
	Kohima	44.0	84.5	69.1	99.4	91.2	58.8	74.5
	Peren	36.3	62.6	74.2	97.3	82.0	24.9	62.9
	Senapati (Excluding 3	37.0	35.8	59.1	92.7	86.6	3.9	52.5

**Manipur**	Tamenglong	32.7	41.0	61.7	87.9	74.0	17.2	52.4
	Churachandpur	42.3	57.4	65.8	89.0	88.1	8.1	58.5
	Bishnupur	48.9	64.5	37.6	96.4	89.5	51.7	64.8
	Thoubal	48.2	60.0	44.3	85.1	87.5	42.2	61.2
	Imphal West	55.6	82.7	46.9	97.2	94.8	64.8	73.7
	Imphal East	50.4	73.2	42.7	96.9	89.7	44.3	66.2
	Ukhrul	31.8	45.5	59.3	79.2	88.9	18.6	53.9
	Chandel	39.4	42.2	61.3	93.9	80.7	13.7	55.2
**Mizoram**	Mamit	39.7	83.3	65.9	86.0	81.7	18.7	62.5
	Kolasib	51.4	97.2	86.6	98.8	95.1	70.2	83.2
	Aizawl	58.9	96.3	90.9	99.7	99.0	80.6	87.6
	Champhai	46.3	97.7	87.0	99.8	97.6	42.4	78.5
	Serchhip	51.0	96.2	91.9	99.6	99.3	64.5	83.8
	Lunglei	47.6	94.1	77.6	99.1	94.4	42.8	75.9
	Lawngtlai	34.1	81.6	65.3	74.3	70.9	20.1	57.7
	Saiha	44.5	93.1	79.7	99.3	94.9	46.4	76.3
**Tripura**	West Tripura	43.8	97.6	68.9	96.1	90.1	43.4	73.3
	South Tripura	37.6	91.5	61.0	92.3	86.3	17.2	64.3
	Dhalai	33.6	66.0	49.2	88.5	78.7	11.7	54.6
	North Tripura	35.8	64.3	45.7	86.0	87.4	20.1	56.5
**Meghalay**	West Garo Hills	41.6	51.5	37.2	92.9	80.0	8.5	51.9
	East Garo Hills	37.2	40.8	40.9	86.3	75.7	14.0	49.2
	South Garo Hills	46.6	67.9	75.4	98.9	88.5	10.7	64.7
	West Khasi Hills	30.7	72.7	79.6	89.8	89.2	12.5	62.4
	Ribhoi	30.1	70.5	56.2	85.9	78.8	7.5	54.8

	East Khasi Hills	37.9	90.9	65.6	95.3	91.9	50.1	72.0
	Jaintia Hills	32.1	77.4	55.5	85.4	77.5	9.4	56.2
**Assam**	Kokrajhar	35.6	76.5	38.8	74.1	71.3	6.3	50.4
	Dhubri	32.1	90.5	32.8	69.4	64.9	10.6	50.1
	Goalpara	37.8	87.2	45.5	71.3	74.9	15.9	55.4
	Barpeta	36.9	98.5	34.0	72.3	70.4	10.3	53.7
	Morigaon	36.5	98.0	34.6	77.7	79.2	9.7	56.0
	Nagaon	39.2	92.9	45.3	83.4	79.7	12.4	58.8
	Sonitpur	40.8	71.3	58.2	79.6	75.8	8.6	55.7
	Lakhimpur	39.1	73.4	47.9	77.6	83.8	12.5	55.7
	Dhemaji	35.0	91.7	38.6	63.5	78.2	8.1	52.5
	Tinsukia	39.5	96.0	50.6	76.3	66.6	22.6	58.6
	Dibrugarh	40.9	98.8	54.5	75.4	76.5	20.0	61.0
	Sivasagar	42.4	96.2	54.5	78.7	81.8	10.4	60.7
	Jorhat	46.0	88.2	63.1	87.1	85.3	23.6	65.5
	Golaghat	42.8	94.3	59.1	86.8	83.5	11.3	63.0
	Karbi Anglong	36.4	53.6	41.5	81.7	68.1	12.5	49.0
	Dima Hasao	33.5	50.5	56.6	79.2	76.8	31.8	54.7
	Cachar	38.3	61.6	36.8	67.5	79.8	18.4	50.4
	Karimganj	36.1	63.2	38.5	71.3	82.9	10.0	50.3
	Hailakandi	32.4	51.2	32.0	55.4	79.9	8.2	43.2
	Bongaigaon	39.9	74.5	45.5	88.4	75.1	16.3	56.6
	Chirang	36.7	71.0	32.1	72.0	69.1	8.5	48.2
	Kamrup	41.3	94.0	52.0	88.8	79.9	11.0	61.2
	Kamrup Metropolitan	52.7	83.6	60.5	95.0	87.3	83.1	77.0

	Nalbari	41.4	98.4	50.4	84.0	82.4	10.7	61.2
	Baksa	39.9	83.1	53.2	81.8	77.9	1.4	56.2
	Darrang	36.3	97.0	45.1	77.0	73.9	8.3	56.3
	Udalguri	38.2	81.2	53.3	84.8	71.0	5.5	55.7
West Bengal	Darjiling	42.8	72.8	60.9	95.4	81.7	40.6	65.7
	Jalpaiguri	35.6	85.8	48.7	89.9	70.7	26.2	59.5
	Koch Bihar	34.3	98.9	49.5	90.0	76.5	10.4	59.9
	Uttar Dinajpur	32.4	98.1	31.8	90.0	54.7	12.4	53.2
	Dakshin Dinajpur	34.4	100.0	42.7	93.1	75.6	13.3	59.8
	Maldah	33.9	91.2	43.6	94.1	71.4	14.3	58.1
	Murshidabad	33.6	100.0	50.6	92.5	73.6	21.7	62.0
	Birbhum	32.6	97.5	28.0	96.0	71.9	13.6	56.6
	Barddhaman	38.0	96.9	46.6	94.4	73.1	41.9	65.2
	Nadia	40.0	99.9	68.5	95.6	80.0	27.5	68.6
	North Twenty Four Par	45.7	100.0	68.9	97.0	89.2	59.3	76.7
	Hugli	41.2	100.0	54.6	95.2	82.5	38.7	68.7
	Bankura	33.2	97.9	29.5	89.4	71.2	10.2	55.2
	Puruliya	24.9	82.7	12.3	79.3	54.2	16.0	44.9
	Haora	45.9	99.8	59.6	97.8	85.9	62.3	75.2
	Kolkata	50.1	100.0	50.1	99.8	86.2	100.0	81.0
	South Twenty Four Par	35.3	99.9	51.7	87.4	82.8	25.9	63.8
	Paschim Medinipur	36.0	96.5	40.4	95.7	79.0	11.7	59.9
	Purba Medinipur	38.3	99.8	66.8	97.5	84.2	11.7	66.4
	Garhwa	25.0	89.1	10.8	49.9	52.8	4.8	38.8
	Chatra	26.1	66.7	13.1	42.0	56.5	5.9	35.0

Jharkhan	Kodarma	39.8	80.6	28.6	91.3	63.4	23.4	54.5
	Giridih	31.8	65.6	17.7	90.1	55.1	9.8	45.0
	Deoghar	33.6	87.4	22.3	80.4	60.6	21.4	51.0
	Godda	25.4	84.9	17.1	78.3	48.2	5.3	43.2
	Sahibganj	26.7	80.1	24.7	72.4	46.5	14.9	44.2
	Pakur	25.5	89.5	13.3	77.6	44.2	8.5	43.1
	Dhanbad	39.7	79.9	28.6	95.9	69.8	59.6	62.2
	Bokaro	38.9	81.4	35.7	87.3	66.2	50.9	60.1
	Lohardaga	29.5	71.5	19.4	81.9	63.1	12.1	46.3
	Purbi Singhbhum	40.7	91.7	43.4	91.5	74.6	55.6	66.2
	Palamu	26.3	91.0	17.2	62.4	59.4	12.6	44.8
	Latehar	23.2	76.4	12.0	53.3	55.7	6.7	37.9
	Hazaribagh	37.7	67.5	26.7	96.4	66.6	18.4	52.2
	Ramgarh	38.7	70.1	38.7	94.9	71.2	43.8	59.6
	Dumka	24.6	89.3	12.1	69.1	56.0	7.2	43.1
	Jamtara	27.8	90.5	14.0	81.4	56.1	9.3	46.5
	Ranchi	40.3	76.0	37.7	92.2	77.2	42.3	61.0
	Khunti	24.9	53.5	13.7	68.6	60.7	8.4	38.3
	Gumla	26.8	47.5	16.3	77.0	64.9	6.9	39.9
	Simdega	26.0	60.0	8.5	54.9	62.0	9.5	36.8
	Pashchimi Singhbhum	23.2	69.3	16.1	68.0	52.5	15.3	40.7
	Saraikela Kharsawan	33.8	81.5	27.0	85.5	67.4	33.6	54.8
	Bargarh	33.8	96.8	26.4	83.5	77.8	9.0	54.5
	Jharsuguda	42.6	93.4	40.3	91.8	81.7	39.1	64.8
	Sambalpur	36.1	89.2	33.7	87.6	79.0	28.8	59.1

Odisha	Debagarh	29.9	85.6	30.9	81.5	73.5	5.6	51.2
	Sundargarh	39.9	89.5	37.9	86.0	72.9	35.2	60.2
	Kendujhar	30.3	85.8	20.5	75.0	68.7	16.2	49.4
	Mayurbhanj	26.3	81.5	18.1	73.9	62.4	7.1	44.9
	Baleshwar	34.8	98.0	37.1	88.7	80.5	11.6	58.4
	Bhadrak	31.5	99.5	22.8	87.2	80.1	12.3	55.6
	Kendrapara	33.9	98.9	25.6	92.4	85.8	5.1	57.0
	Jagatsinghapur	36.4	97.3	30.7	92.9	88.8	9.4	59.2
	Cuttack	40.5	92.0	38.8	91.6	89.0	26.8	63.1
	Jajapur	37.7	88.2	30.7	93.2	81.2	7.9	56.5
	Dhenkanal	36.4	59.4	33.3	88.7	79.9	10.2	51.3
	Anugul	39.0	77.2	35.6	87.4	77.0	17.4	55.6
	Nayagarh	35.9	80.2	31.4	93.4	82.6	8.2	55.3
	Khordha	45.3	85.0	47.0	96.4	84.2	54.8	68.8
	Puri	40.0	96.2	40.1	95.0	86.9	15.4	62.3
	Ganjam	35.5	90.7	40.7	89.4	70.4	21.4	58.0
	Gajapati	24.9	69.4	38.2	87.7	49.2	13.5	47.1
	Kandhamal	23.7	61.4	16.4	77.6	58.0	8.2	40.9
	Baudh	30.4	88.8	16.4	86.9	72.4	3.8	49.8
	Subarnapur	31.9	96.7	15.9	89.1	77.8	7.2	53.1
	Balangir	32.6	94.3	14.1	82.6	67.0	11.3	50.3
	Nuapada	30.1	95.4	20.2	82.3	56.5	5.5	48.3
	Kalahandi	25.9	94.1	14.9	67.1	50.4	7.0	43.2
	Rayagada	27.4	91.7	23.4	79.1	39.2	16.0	46.1
	Nabarangapur	21.5	98.4	16.1	69.0	42.9	8.1	42.7

	Koraput	22.8	84.7	18.2	76.0	42.2	14.9	43.1
	Malkangiri	25.3	89.5	16.7	86.0	36.8	7.7	43.7
**Chhattisgarh**	Korea (Koriya)	33.5	75.1	23.0	90.7	67.9	30.4	53.4
	Surguja	28.4	72.6	18.0	92.9	59.8	10.9	47.1
	Jashpur	26.7	83.3	14.9	84.1	69.1	7.3	47.6
	Raigarh	37.3	92.9	26.7	95.1	77.1	14.2	57.2
	Korba	41.0	80.6	34.1	93.9	72.7	36.7	59.8
	Janjgir - Champa	41.4	95.3	28.9	98.2	77.5	13.7	59.1
	Bilaspur	44.6	92.0	39.9	99.3	74.3	29.0	63.2
	Kabirdham	34.5	93.5	20.8	95.2	58.2	11.0	52.2
	Rajnandgaon	45.5	92.9	44.8	98.9	81.3	20.6	64.0
	Durg	48.3	98.5	44.7	99.1	81.2	39.9	68.6
	Raipur	43.8	95.3	38.6	98.0	75.0	38.4	64.8
	Mahasamund	36.5	96.8	22.0	98.0	71.1	10.4	55.8
	Dhamtari	41.0	95.8	54.4	98.2	80.8	23.3	65.6
	Uttar Bastar Kanker	38.2	98.0	38.5	95.8	74.1	11.9	59.4
	Bastar	27.1	94.5	17.5	85.9	50.5	13.7	48.2
	Narayanpur	28.7	92.5	15.0	77.6	56.3	18.1	48.0
	Dakshin Bastar Dantew	24.6	95.7	22.4	86.8	41.1	18.3	48.1
	Bijapur	31.8	93.9	21.6	91.6	72.3	12.1	53.9
	Sheopur	34.1	93.1	15.4	83.1	47.8	14.8	48.0
	Morena	45.4	92.5	36.9	87.9	62.7	28.6	59.0
	Bhind	42.7	93.2	30.6	87.9	65.7	32.0	58.7
	Gwalior	52.5	97.3	56.1	95.5	71.8	66.7	73.3
	Datia	41.7	91.9	33.5	89.6	65.4	23.2	57.6

Madhya Pradesh	Shivpuri	36.9	67.1	22.6	87.3	54.9	19.6	48.1
	Tikamgarh	31.8	74.1	13.7	84.9	58.0	16.4	46.5
	Chhatarpur	32.5	72.8	14.5	77.0	59.1	21.6	46.3
	Panna	31.0	78.1	19.5	79.7	63.5	14.0	47.6
	Sagar	33.3	83.6	26.7	83.9	75.9	29.2	55.5
	Damoh	30.7	78.5	22.0	85.7	66.8	23.3	51.2
	Satna	37.9	91.5	28.9	88.9	71.1	21.9	56.7
	Rewa	34.3	88.0	24.1	86.9	68.3	16.6	53.0
	Umaria	30.7	69.0	18.2	79.1	61.4	19.1	46.3
	Neemuch	41.7	77.7	32.0	97.1	62.4	28.3	56.5
	Mandsaur	40.1	72.7	28.1	97.0	62.6	23.7	54.0
	Ratlam	36.7	91.2	33.5	93.5	61.5	31.5	58.0
	Ujjain	44.9	93.2	51.7	97.4	65.0	40.9	65.5
	Shajapur	41.8	86.4	34.7	96.5	58.0	21.5	56.5
	Dewas	47.1	93.6	47.0	98.6	65.6	31.8	64.0
	Dhar	39.9	87.6	35.5	95.7	56.8	25.9	56.9
	Indore	58.1	99.7	74.1	99.1	78.0	81.1	81.7
	Khargone (West Nimar)	42.7	91.0	33.1	97.5	60.8	18.8	57.3
	Barwani	34.2	85.2	21.1	90.2	50.1	20.2	50.2
	Rajgarh	39.7	67.0	19.0	95.0	60.2	19.3	50.0
	Vidisha	31.6	94.2	23.1	84.7	65.8	27.3	54.4
	Bhopal	57.2	97.4	61.7	98.3	79.9	80.7	79.2
	Sehore	48.7	89.7	47.0	98.0	63.3	20.4	61.2
	Raisen	42.9	95.1	39.0	92.8	72.8	27.4	61.6
	Betul	37.5	85.7	30.5	92.0	74.0	20.7	56.7

Harda	43.5	87.9	51.3	96.5	67.5	23.7	61.7
Hoshangabad	47.0	91.2	46.2	94.4	74.7	32.1	64.2
Katni	32.8	90.6	23.1	82.3	70.6	18.9	53.1
Jabalpur	44.5	96.7	47.2	94.6	81.3	56.5	70.1
Narsimhapur	35.0	97.3	39.5	91.6	74.2	22.7	60.1
Dindori	19.3	71.3	6.9	73.4	56.5	4.8	38.7
Mandla	25.4	62.9	15.0	76.9	66.3	13.6	43.4
Chhindwara	36.0	82.3	33.6	89.3	71.4	26.8	56.6
Seoni	30.4	78.4	24.3	81.9	71.6	14.3	50.1
Balaghat	35.7	76.4	28.1	88.2	80.7	15.3	54.1
Guna	40.4	82.9	27.8	93.1	55.3	25.7	54.2
Ashoknagar	40.3	91.7	19.8	88.3	58.0	18.0	52.7
Shahdol	29.5	66.6	16.6	75.3	59.2	17.6	44.1
Anuppur	33.2	69.4	21.7	79.0	64.4	25.6	48.9
Sidhi	29.8	70.3	9.8	77.0	61.9	8.5	42.9
Singrauli	32.1	56.5	15.8	71.0	54.0	21.3	41.8
Jhabua	24.1	85.3	12.9	88.6	29.0	9.8	41.6
Alirajpur	30.0	89.1	17.6	92.5	29.9	9.4	44.7
Khandwa (East Nimar)	40.7	78.4	34.1	90.3	62.5	21.6	54.6
Burhanpur	39.8	93.9	40.0	90.7	67.9	38.5	61.8
Kachchh	41.7	95.0	61.5	95.4	62.6	34.8	65.2
Banaskantha	35.1	98.9	38.7	87.3	59.8	14.3	55.7
Patan	37.9	97.3	56.3	93.2	65.1	22.1	62.0
Mahesana	45.7	96.6	66.1	96.8	81.1	28.3	69.1
Sabarkantha	39.3	95.7	46.5	95.2	73.0	16.1	61.0

**Gujarat**	Gandhinagar	44.3	99.6	62.3	94.8	72.4	49.8	70.5
	Ahmadabad	54.4	99.0	88.5	99.2	86.9	84.7	85.4
	Surendranagar	43.3	89.7	47.7	95.6	69.0	30.8	62.7
	Rajkot	51.5	98.3	72.3	98.6	84.5	60.3	77.6
	Jamnagar	47.3	98.3	68.0	98.0	76.3	47.6	72.6
	Porbandar	48.8	94.2	72.3	99.3	81.9	47.4	74.0
	Junagadh	47.2	98.9	75.1	99.3	80.2	34.9	72.6
	Amreli	49.6	98.1	74.8	98.9	78.6	27.1	71.2
	Bhavnagar	46.3	96.9	65.8	99.5	70.2	45.5	70.7
	Anand	43.2	99.7	64.2	97.6	81.7	31.4	69.6
	Kheda	37.6	99.0	51.3	94.5	77.2	23.8	63.9
	Panchmahal	36.6	81.2	39.8	85.9	69.6	17.6	55.1
	Dohad	31.0	73.3	21.9	78.5	55.7	10.8	45.2
	Vadodara	45.4	97.7	60.7	97.0	76.7	53.2	71.8
	Narmada	35.8	98.1	34.7	91.3	74.7	11.8	57.7
	Bharuch	44.4	98.8	66.4	96.1	77.4	37.4	70.1
	The Dangs	26.3	71.2	19.3	87.0	71.2	10.5	47.6
	Navsari	47.9	95.4	68.6	97.8	87.0	31.7	71.4
	Valsad	45.4	92.5	61.2	98.3	79.5	39.2	69.3
	Surat	49.2	99.9	77.4	98.3	85.8	79.0	81.6
	Tapi	39.0	93.9	38.6	91.6	73.6	9.6	57.7
**Daman and Diu**	Diu	50.8	99.5	71.4	99.8	84.1	48.7	75.7
	Daman	41.7	99.3	58.2	100.0	85.2	83.9	78.1
**Dadra and Nagar Haveli**	Dadra & Nagar Haveli	40.2	93.2	35.4	97.4	69.2	51.0	64.4
	Nandurbar	28.0	84.1	23.3	74.1	54.2	10.5	45.7

	Dhule	39.1	94.0	31.9	87.3	69.3	23.1	57.4
	Jalgaon	42.9	96.7	42.1	90.2	80.5	26.5	63.1
	Buldana	41.4	87.8	45.7	96.0	82.8	21.7	62.6
	Akola	44.3	99.2	46.4	93.8	92.9	34.0	68.4
	Washim	40.1	88.2	41.5	94.0	84.8	17.2	61.0
	Amravati	45.7	95.9	64.4	92.7	90.8	35.6	70.9
	Wardha	47.0	93.7	56.9	95.3	89.4	32.0	69.0
	Nagpur	54.3	97.7	71.1	97.2	94.3	69.8	80.7
	Bhandara	47.7	88.4	67.3	96.3	94.0	29.4	70.5
	Gondiya	42.9	79.0	55.4	96.2	90.8	15.6	63.3
	Gadchiroli	38.0	86.3	32.6	93.5	78.3	12.7	56.9
	Chandrapur	44.2	85.7	53.9	91.6	84.3	33.4	65.5
	Yavatmal	40.1	81.0	43.6	89.6	82.5	20.5	59.5
	Nanded	38.2	92.4	43.8	88.2	79.4	26.3	61.4
	Hingoli	37.7	86.6	40.0	84.3	75.3	13.1	56.2
	Parbhani	39.8	93.0	34.0	91.7	74.4	30.7	60.6
**Maharashtra**	Jalna	36.0	76.8	33.9	84.0	74.1	17.8	53.8
	Aurangabad	45.4	94.8	45.3	95.7	81.0	47.4	68.2
	Nashik	45.3	91.1	52.8	92.2	88.3	55.1	70.8
	Thane	48.0	93.3	64.7	90.6	82.7	81.9	76.9
	Mumbai Suburban	43.2	99.6	26.3	92.1	86.3	100.0	74.6
	Mumbai	46.4	99.9	39.1	99.2	93.3	100.0	79.7
	Raigarh	50.1	96.5	75.4	94.0	87.2	54.5	76.3
	Pune	50.6	97.4	63.3	95.9	89.8	64.4	76.9
	Ahmadnagar	46.0	90.1	50.8	92.2	87.2	21.6	64.7

	Bid	37.5	88.9	39.5	90.6	73.3	20.8	58.4
	Latur	36.3	96.1	39.7	93.1	81.7	20.7	61.3
	Osmanabad	36.8	92.2	27.3	87.8	82.4	13.3	56.6
	Solapur	42.3	88.3	48.9	88.9	82.8	31.4	63.8
	Satara	43.7	93.7	62.5	93.6	89.6	21.5	67.4
	Ratnagiri	39.8	85.8	67.5	96.1	89.9	16.1	65.9
	Sindhudurg	43.3	75.3	77.6	98.2	94.5	14.6	67.2
	Kolhapur	44.8	95.6	66.9	96.1	88.4	33.5	70.9
	Sangli	45.9	96.1	68.6	91.6	88.4	30.0	70.1
**Telangana**	Adilabad	36.5	91.0	30.0	96.5	55.1	28.0	56.2
	Nizamabad	36.4	99.9	42.3	97.7	61.0	26.6	60.6
	Karimnagar	41.4	93.9	53.8	98.8	69.9	31.9	65.0
	Medak	36.4	99.6	53.3	97.5	60.6	30.0	62.9
	Hyderabad	54.6	99.7	72.6	100.0	82.8	100.0	85.0
	Rangareddy	49.3	100.0	51.2	99.5	76.7	76.7	75.6
	Mahbubnagar	33.2	97.9	32.8	97.8	49.0	14.0	54.1
	Nalgonda	39.3	99.6	50.1	97.3	60.6	21.2	61.4
	Warangal	38.9	93.3	46.8	97.4	69.8	31.2	62.9
	Khammam	43.3	98.7	50.3	97.2	63.4	26.2	63.2
	Srikakulam	37.8	81.5	33.2	97.3	64.6	16.0	55.1
	Vizianagaram	39.6	94.1	31.9	98.1	59.5	23.3	57.8
	Visakhapatnam	44.6	91.4	55.2	98.5	67.1	48.6	67.6
	East Godavari	46.3	97.6	56.8	99.1	72.7	26.5	66.5
	West Godavari	43.7	97.6	61.2	98.7	74.0	20.7	66.0
	Krishna	45.0	91.9	63.1	99.0	76.5	37.3	68.8

**Andhra Pradesh**	Guntur	42.8	98.2	63.2	99.5	62.7	35.6	67.0
	Prakasam	40.0	93.8	53.6	97.7	58.7	19.7	60.6
	Sri Potti Sriramulu N	42.0	94.8	52.7	97.9	64.5	29.8	63.6
	Y.S.R.	44.7	99.6	68.5	99.7	64.8	36.4	69.0
	Kurnool	41.8	98.5	52.9	99.7	56.4	33.3	63.8
	Anantapur	40.7	99.5	46.5	99.1	61.9	32.0	63.3
	Chittoor	41.3	99.8	39.8	98.6	73.2	29.6	63.7
**Karnataka**	Belgaum	43.6	99.0	40.2	98.1	77.2	28.9	64.5
	Bagalkot	37.5	98.0	23.0	96.9	65.5	34.3	59.2
	Bijapur	36.2	92.3	22.9	96.1	64.3	26.2	56.3
	Bidar	39.2	98.0	26.7	97.4	74.6	28.7	60.8
	Raichur	37.4	89.9	27.6	97.6	50.4	29.9	55.5
	Koppal	38.5	97.4	48.9	98.2	64.6	22.1	61.6
	Gadag	34.6	94.4	30.2	97.4	71.4	41.1	61.5
	Dharwad	45.0	98.6	62.0	98.1	79.9	61.3	74.1
	Uttara Kannada	44.3	69.5	65.7	96.7	86.1	31.3	65.6
	Haveri	39.1	98.5	53.5	97.1	77.7	23.7	64.9
	Bellary	40.4	98.2	40.6	98.1	58.1	42.5	63.0
	Chitradurga	38.3	99.0	44.0	96.9	78.1	26.3	63.8
	Davanagere	43.2	99.6	64.0	98.0	75.9	37.2	69.6
	Shimoga	45.8	84.6	71.3	95.6	80.4	35.0	68.8
	Udupi	51.7	65.5	89.0	98.4	90.5	29.2	70.7
	Chikmagalur	41.3	91.0	62.7	96.3	78.5	21.3	65.2
	Tumkur	40.1	98.9	52.3	98.1	77.1	25.5	65.4
	Bangalore	57.0	97.8	86.2	99.4	89.6	91.9	87.0

	Mandya	43.2	98.1	56.8	98.9	76.3	19.9	65.5
	Hassan	43.9	97.2	63.9	96.3	78.7	24.1	67.3
	Dakshina Kannada	50.2	86.9	91.3	97.8	89.0	50.7	77.6
	Kodagu	45.8	83.5	82.0	95.8	86.0	12.4	67.6
	Mysore	43.0	98.3	63.6	97.2	74.1	43.2	69.9
	Chamarajanagar	34.8	98.8	32.8	93.8	63.8	18.2	57.0
	Gulbarga	37.9	99.0	29.1	98.2	61.0	39.7	60.8
	Yadgir	33.8	92.6	17.8	97.9	45.4	23.3	51.8
	Kolar	42.8	98.9	58.1	98.7	75.8	39.3	68.9
	Chikkaballapura	40.9	98.4	49.7	97.5	69.8	26.0	63.7
	Bangalore Rural	44.8	99.3	74.3	98.3	80.7	27.4	70.8
	Ramanagara	44.2	97.6	60.8	97.8	74.3	24.0	66.5
**Goa**	North Goa	57.7	96.9	79.8	99.6	93.3	62.7	81.7
	South Goa	57.2	97.2	75.9	100.0	87.7	65.7	80.6
**Lakshadweep**	Lakshadweep	65.8	91.6	99.1	99.9	98.6	78.9	89.0
**Kerala**	Kasaragod	58.7	93.0	97.7	98.5	96.6	39.5	80.6
	Kannur	61.9	92.4	98.8	98.9	99.7	61.4	85.5
	Wayanad	50.1	93.4	95.1	96.0	94.0	4.0	72.1
	Kozhikode	64.5	96.6	99.4	99.2	99.3	65.1	87.4
	Malappuram	62.7	93.8	98.7	99.5	99.2	49.2	83.8
	Palakkad	57.6	93.9	96.6	99.0	98.5	20.7	77.7
	Thrissur	65.9	96.0	99.5	99.7	99.6	65.9	87.8
	Ernakulam	67.9	97.0	99.2	99.9	99.7	66.2	88.3
	Idukki	55.8	93.1	94.2	99.2	95.9	4.2	73.7
	Kottayam	62.7	97.2	98.9	99.6	99.9	25.8	80.7

	Alappuzha	60.7	94.7	97.0	99.5	99.5	52.5	84.0
	Pathanamthitta	61.5	93.7	97.4	99.2	99.7	9.2	76.8
	Kollam	60.3	94.6	97.5	98.2	99.3	46.5	82.7
	Thiruvananthapuram	59.2	93.0	96.8	99.3	99.5	54.5	83.7
Tamil Nadu	Thiruvallur	51.3	99.4	66.6	98.9	89.2	67.8	78.9
	Chennai	59.4	97.8	82.3	99.6	92.3	100.0	88.6
	Kancheepuram	54.7	96.8	68.0	99.7	86.4	66.8	78.7
	Vellore	47.7	98.9	53.4	99.5	84.9	47.4	72.0
	Tiruvannamalai	39.3	99.5	31.0	98.8	78.5	20.3	61.2
	Viluppuram	40.2	99.5	32.4	99.3	74.1	16.5	60.3
	Salem	45.4	95.6	46.2	99.0	77.7	49.1	68.8
	Namakkal	46.7	97.0	49.4	99.4	80.6	41.6	69.1
	Erode	46.4	99.9	61.1	98.4	79.7	53.2	73.1
	The Nilgiris	40.7	95.1	63.0	97.2	89.4	58.1	73.9
	Dindigul	40.9	99.3	42.6	97.1	78.9	39.7	66.4
	Karur	46.6	98.5	47.9	98.7	82.0	43.3	69.5
	Tiruchirappalli	47.1	100.0	46.5	98.9	88.7	48.9	71.7
	Perambalur	41.4	93.8	37.1	98.8	79.6	17.4	61.3
	Ariyalur	38.4	99.3	28.1	98.3	77.3	10.7	58.7
	Cuddalore	41.9	99.5	38.6	98.9	84.0	34.9	66.3
	Nagapattinam	41.5	94.3	43.1	98.4	85.7	23.7	64.5
	Thiruvarur	42.2	99.8	45.2	97.0	89.9	20.8	65.8
	Thanjavur	42.6	99.3	45.8	97.0	86.7	35.6	67.8
	Pudukkottai	40.3	92.6	35.2	97.0	81.8	21.2	61.4
	Sivaganga	43.1	91.4	47.6	98.3	87.1	30.4	66.3

	Madurai	45.3	92.6	54.4	98.8	85.4	64.3	73.5
	Theni	42.1	97.8	47.0	97.4	80.1	63.1	71.3
	Virudhunagar	40.5	97.4	36.4	98.6	78.3	50.1	66.9
	Ramanathapuram	38.6	94.2	44.5	98.2	84.6	29.9	65.0
	Thoothukkudi	43.0	99.0	50.3	98.2	88.3	49.0	71.3
	Tirunelveli	40.7	99.5	45.8	99.1	85.5	50.4	70.2
	Kanniyakumari	49.7	94.9	85.6	99.3	98.5	83.0	85.2
	Dharmapuri	44.2	99.6	37.7	98.4	78.4	22.8	63.5
	Krishnagiri	44.9	99.6	43.8	99.0	76.2	25.1	64.8
	Coimbatore	47.4	97.2	57.6	98.7	84.2	75.1	76.7
	Tiruppur	45.9	99.6	51.5	99.5	81.5	64.5	73.8
**Puducherry**	Yanam	50.9	100.0	73.1	99.5	81.3	100.0	84.1
	Puducherry	50.9	99.8	62.5	99.6	88.1	70.8	78.6
	Mahe	57.9	90.0	98.2	99.7	99.8	100.0	90.9
	Karaikal	49.8	100.0	69.1	99.5	92.6	49.8	76.8
**Andaman and Nicobar**	Nicobars	44.9	99.8	86.7	99.4	85.1	0.0	69.3
	North & Middle Andama	41.6	90.4	49.8	92.5	91.0	3.6	61.5
	South Andaman	51.4	96.2	83.6	98.7	90.7	63.3	80.7
