# Supplementary material for: District level inequality in reproductive, maternal, neonatal and child health coverage in India
Source: BMC Public Health. 2020 Jan 14;20:58. doi: 10.1186/s12889-020-8151-9 (PMC6961337; doi:10.1186/s12889-020-8151-9)
Supplement: Supplementary file 4 — Additional file 4: Performance of asset index and percentage coverage of associated assets by districts of India in 2015–16. [file 12889_2020_8151_MOESM4_ESM.pdf]

S3 Table: Asset index and percentage coverage of associated assets by districts of India in 2015-16.

States	District	Chair	Table	Cot	Radio	Bicycle	Watch	Mattress	Presser Cooker	Motor Cycle	Electric Fan	Swing Machine	Colour TV	Refrigerator	Mobile	Car/Truck	Internet	AC	Washing Machine	Computer	Water Purifier	Assert Index
Jammu and Kashmir	Kupwara	34	25.2	32	54.8	7.1	75.9	91.1	80.4	4.4	45.8	15.6	43.7	21.6	96.3	11.5	29.9	1.9	17.7	6.3	6.9	33.7
	Badgam	21.8	20.9	27.5	62.5	16.5	76.5	99.8	91.2	12.7	68.6	19.1	66.8	29.9	98.4	15.5	30.4	2.7	29.3	11.8	6.6	38.8
	Leh	54	69.9	60	69	6.4	75.5	98.6	97.6	5.9	8.9	18.9	78.8	31.2	94.9	29	21.2	0.2	39.7	9.4	2.3	41.6
	Kargil	24.1	21.5	24.2	67.2	4.6	78.4	85.8	89.5	4.9	10	22.5	62.2	10.9	96.3	17.6	21.2	0.5	15.4	10.3	3.3	32.1
	Punch	96.6	91.5	96.1	43.8	5.3	64.3	94.3	84.2	13.3	61.9	31.1	48	32.7	96.8	9	14	11.3	22.2	5.1	1.6	44
	Rajouri	92.7	85.3	94.9	37	6.8	65.7	82.2	77.3	17.4	71.3	42.3	51	39.7	94.9	9.6	16.3	15.2	22.1	6.3	2.3	44.4
	Kathua	90.6	82.5	98.6	16.9	18.5	88.9	69.8	93.3	37.1	86.6	67.4	77	63.5	97.8	11.5	28.1	50.4	28.8	8.5	19	54.9
	Baramula	45.6	37.5	43.2	58.6	11.9	78	90.2	93.8	10.1	69.9	18.8	75.4	36.6	95.1	13	20.4	4	33.8	9.1	6.5	40.9
	Bandipore	30.8	24.7	29.2	56.1	21.1	78.6	98.6	85.1	10	63	19.4	68.1	25.2	95.3	11	36.5	3.8	22.4	7.1	5.9	38
	Srinagar	63.2	62.3	66.5	62.7	19.2	91.9	99.1	99.3	35.9	96.1	22.4	92.7	80.4	98.9	25.7	48.9	15.1	76.4	21.9	7.8	56.9
	Ganderbal	32.5	25.8	33.1	61.1	25.9	79.7	99.7	90.6	17.4	73.3	16.5	67.6	36.3	96.9	14.9	31.2	1.6	31.4	11.3	4.1	40.7
	Pulwama	29	30	34	60.3	12.9	85.1	99.2	97.1	17.7	80	18.9	67.3	41.7	98	15.8	40.9	4	37.1	11.2	21.8	44
	Shupiyan	25.1	18.4	25.2	69	7.5	78.7	98.4	91.8	23.3	50.2	18.8	59.8	26.3	98.5	19.1	36.5	1.8	31.1	9.6	11.6	38.7
	Anantnag	23.4	20.6	29.3	49.1	7.9	72.3	97.9	91.7	10.2	58.6	21.5	62.4	26.8	95.7	13.1	35.2	1.9	30	9.3	7.4	36.7
	Kulgam	18	16.4	21.6	63.2	8.1	75.7	98.9	92.9	10	46.2	17.2	53.8	13.9	98.2	12.3	33.1	0.5	19.7	7.4	7.1	34.3
	Doda	68.6	50.9	78.5	24.6	3.5	57.3	51.3	80.3	5.1	37.8	33.8	38.1	20.4	93.9	5.5	19.2	1.3	6.5	3.1	0.5	32.4

	Ramban	59.2	43.3	77.9	31.4	2.2	69.9	53.5	86	2.8	24.5	30.4	35.8	15.6	93	5.5	18.4	1.9	7.7	2.9	0.7	31.6
	Kishtwar	65.4	49.5	75.9	29.8	6.4	74.9	57.8	89.8	8.2	36.8	39.6	46.4	26.7	90.1	7	26	3.4	12	7.5	1.1	36
	Udhampur	85.1	77.8	97.9	22.3	9.8	86.1	62.7	85	19.9	74.2	69.4	62	50.8	96.1	8.9	19.1	37.1	16.9	4.4	9.2	47.8
	Reasi	88.6	81.2	97.6	41.4	5.5	69.5	95	87.1	18.2	69	44.3	53.1	35.4	96.9	9.7	12.7	24.5	16.8	4	1.3	45.4
	Jammu	92.1	87.3	97.2	14.4	31.7	90	81.8	94.2	54.2	95.8	66.3	88.3	82.2	97.2	27.3	43.3	71.5	52.9	24.9	32	64.6
	Samba	87.1	79.7	95.5	14.1	31.1	82.7	69.5	89.9	42.6	90.8	72.2	76.4	74.1	93.6	14.1	26.3	57.3	40.3	9.5	29.5	57.4
	Chamba	90.3	79.7	94.6	11.4	2.2	76.8	90.4	97.1	19.7	51.6	64.2	78.8	49.6	96.4	10.6	25	2.8	21.5	8.7	1.4	46.4
	Kangra	95.6	90.7	97.3	18.2	15.1	87.9	94.7	98.4	40.4	93.5	80.2	90.8	78	97.3	14.1	35.4	14.2	40.2	13.5	9.5	57.8
	Lahul And Spiti	46.9	52.7	86.3	19.4	2	86.4	93.5	99.6	10.8	0.7	39.5	83.3	18.4	95.9	17.3	19.7	0	5.8	5.2	3.5	37.6
	Kullu	75.8	62	85	13	4.2	75.7	89.2	96.5	10.5	30.6	57.1	82.7	39	95.2	16.6	27.6	1.3	18	10.8	4.1	42.8
	Mandi	89.6	76.8	92.4	15.2	3.8	73.3	89.8	96.9	20.7	58.3	75.5	79.7	48.2	95.6	12.9	21.9	1.8	22.9	9.4	8.2	47.7
**Himachal Pradesh**	Hamirpur	97.9	92.3	99.1	20.1	7.7	88.2	95.6	97.1	35.4	95.7	86.3	87.4	74.5	96.5	14.9	5.2	9.2	47	11.9	14	56.7
	Una	93	88.4	98.5	15.1	30	84.5	86.7	93.8	52.2	97.4	84.1	87.9	74	96.8	15.7	5.7	27.2	39	10.4	15.5	57.7
	Bilaspur	95.6	90.6	97.9	15.8	7.6	81.6	93.3	96.1	27.3	97.1	84.1	81.4	69.7	97.4	14.8	4.8	11.5	35	11.8	13.7	54.3
	Solan	83.1	76.3	95.8	20.5	8.6	82.2	92.8	96.4	36.3	84.2	67.8	83.9	65.6	98.7	23.1	45.6	25	41.1	19.6	14.8	56
	Sirmaur	86.6	76	91.2	16.1	14.1	75.4	84.5	97.3	35.8	78.1	62.5	75.8	59.4	95.5	14.6	33.4	23.2	30.2	13.2	10.6	51.6
	Shimla	80.1	75.6	91.5	26.6	3.2	77.8	95.7	99.4	9.1	16	60.9	84.6	54.4	99.2	31	54.2	1.3	40.1	22.5	2.3	48.9
	Kinnaur	79.1	77.4	89.7	23.8	1.5	73.8	94.8	98.5	3.5	6.3	46.5	82.8	34.6	96.5	22.8	38.6	0	27.8	10.4	0.6	43.3
	Gurdaspur	94.7	93.6	99.1	2.9	73.3	96.7	92.6	90.8	70.1	98.9	71.9	93.7	86.1	97	14.1	35.2	57.3	52.6	16.5	41.9	67.7
	Kapurthala	91.4	90	97.3	5.3	73.3	94.1	93.6	90.3	71.2	96.1	76.8	90.7	85.2	96.8	18.6	41.8	64.1	59.5	16.3	38.2	68

**Punjab**	Jalandhar	96.7	97.6	99.1	6.1	72.2	98	97.7	94.9	78.4	99.7	79.8	95.6	92.7	99.2	26.9	53.1	77	68.9	31.9	24.7	72.1
	Hoshiarpur	93.3	92.5	98.9	3.8	76	95.7	93.8	92.8	75.2	98.5	78.8	95.4	87.4	98.4	15.9	37.7	65.4	51.7	18.2	31.5	68.2
	Sangrur	94.2	93.7	98.6	7	77	96.2	96.8	93.8	69.9	98.9	81.1	91.8	90	97.2	15.4	36	64.7	48.6	13.2	28.9	67.7
	Fatehgarh Sahib	88.8	89.8	98.5	2.9	74.7	95.5	92.3	89.6	70.7	99.1	73.2	90.9	88.6	97.3	21.9	39.3	68.3	52.6	16.8	39.4	68.1
	Ludhiana	87.1	85	97.2	2.6	68	93.9	90.1	88.8	67.3	99.1	72.4	90.1	81.9	98	20.7	48.7	65.2	53.5	23.8	19.2	65.3
	Moga	90.8	90.3	99	3.5	74.4	96.7	90	80.5	67.5	99.2	70.2	93.3	86.4	97.4	22.5	32	61.9	50.1	21.3	33.5	66.4
	Firozpur	90.8	89.2	98.1	2.3	78	94.1	86.7	71.4	61.5	98.3	69.1	91.1	76.6	96.2	12.6	29.4	54.7	36.6	12.6	29.1	62.3
	Muktsar	87.2	88	99.6	3.6	73.7	96.6	88.2	81.6	62.8	98.4	70.7	90.8	80.4	96.6	24.1	35.7	64.4	48.6	19.1	34.6	65.7
	Faridkot	86.1	89.3	99.7	4.6	72.3	95.4	88.5	79.1	71	99.4	73	92	85.6	96.5	23	35.9	66.4	45.1	14.7	35.7	66.1
	Bathinda	87.8	89	98.8	5.1	70.1	95.6	92.1	75.9	68.3	98.8	73.5	90.8	84.9	97.6	23.5	43.3	68	53.6	24.3	31.6	66.9
	Mansa	87.3	88.9	98.2	3.2	75.4	93.9	88	66.3	57.7	97.9	73	87.4	77.8	95.7	15.6	25.4	56.9	39.5	12.9	23.7	61.3
	Patiala	91.1	88.3	96.9	6.6	62.2	96.9	90.9	87.8	72	99.2	74.9	91.9	86.7	97.1	25.3	42.6	67.7	60.7	24.4	38.6	68.6
	Amritsar	92.9	91.8	98.8	1.8	67	96	92.7	87.4	67.5	98.4	70.9	93.4	86.7	97.6	14.3	38.9	56	53.9	17.1	31.4	66
	Tarn Taran	91.6	90.3	99.5	3.3	79.9	98.4	91.6	84.5	66.8	99	73.3	92.2	81.9	97	18.1	36.9	56.2	46.6	15.3	40.7	66.8
	Rupnagar	95.7	96.2	99.5	3.1	68	95.4	97.5	95.8	74.7	99.1	80.8	94.2	88	98.2	13.7	40.3	70.4	53.3	19.4	29.3	68.6
	Sahibzada Ajit Singh	84.9	84.9	95.3	5	55.6	92.7	93.1	90.7	70.9	98.4	69.1	89.5	84.7	98.7	29.6	53.4	67.7	55.8	29.8	11.1	65.3
	Shahid Bhagat Singh	91.5	90.1	97.9	3	71.7	91.4	90.2	80.7	66.1	98.5	76.8	89	82.7	98	17.8	35.5	65.7	52.4	17.7	38.7	66.4
	Barnala	89.7	90.1	99	4.5	74.7	95.6	89.7	75.9	69.8	98.7	73.8	90.6	84.6	97.5	20.7	35.8	63.4	51.8	18	40.1	66.9
**Chandigarh**	Chandigarh	87.7	86.4	95.9	5.8	50.9	93.4	93.5	95.1	65.1	98.4	62.4	92.8	82.7	98.4	33.3	59.3	79.3	63.5	39.4	4.8	66.3
	Uttarkashi	79.2	64.7	89.3	4	2.8	72.9	94	91.7	14.7	42.3	24.6	62.4	27.2	91.7	3.5	20.5	2.9	9.1	7.2	1.6	38.5

**Uttarakhand**	Chamoli	80.6	68	94.1	6.7	2.6	73.3	96.3	91.7	6.4	40.4	32.4	64.9	23.4	93.9	4.1	16	1.5	9.7	5.7	0.3	38.7
	Rudraprayag	86.2	73.2	94.9	3.8	1.3	74.2	97.3	96.1	6.8	52.9	30.3	71.6	23	96.5	3	17.8	2.3	5.1	5.5	0	40.1
	Tehri Garhwal	89.8	73.9	97.4	3.2	5.9	78.9	98.7	97.1	20	58.2	33.6	73.6	33.9	95.2	4.9	27.7	11.3	12.5	8.1	3	44.3
	Dehradun	90	83.4	93.4	3.2	33	92.7	97.9	97.3	59.4	94.6	54.2	87.2	68.9	98.4	16.2	43.5	36.1	44.6	25.9	20.5	60
	Garhwal	91.2	77.3	98.2	6.3	11.2	85.7	95.5	92	17.6	64.9	36.3	77.2	32.6	95	4.3	23.1	12.8	12.6	10.8	7.9	45.7
	Pithoragarh	81.2	60.7	87.2	5.5	1.5	84.5	94.4	92.6	7.8	34.4	24.1	70.4	21.9	92.1	2.5	10.8	2	10.2	4.9	1.2	37.7
	Bageshwar	81.5	57.3	87	3	2.8	81.9	95.1	91.1	9.5	52.7	25.3	66.6	25.8	94.7	2.4	14.7	2.8	7.7	6.2	1.3	38.6
	Almora	72.7	60.7	89.6	5.6	3	79.8	94.5	88.7	8.3	31.6	29.4	67.5	16.6	94.6	2.4	13.5	1	7	6.1	2	37
	Champawat	77.6	59.2	87.2	6.3	16.5	82.3	92.1	91.2	17.4	36.1	25.8	64.9	24.9	92.9	5.4	17.2	13.1	13.9	9.2	7	40.3
	Nainital	88.8	80.5	96.2	6.1	31	85.6	94.5	90.9	37.3	75	46.8	79.6	52.8	96.8	10.1	31.1	32.3	31.9	18.2	15.6	53.2
	Udham Singh Nagar	82.5	65.1	96.2	5.8	62.3	81.1	90.1	82.7	42.4	88.6	45.6	73.6	39.5	93	7.1	24.4	25.7	24.4	10.5	23.5	51.8
	Hardwar	80	70.2	97.3	4.1	58.7	83.8	89.2	82	48.1	90.3	53.1	70.9	45.2	93	7.5	25.5	27.9	32	10.8	22.9	53.1
	Panchkula	95	90.7	96.7	7.5	42.4	98.6	94.6	95.9	76.2	99.1	76.2	93.9	88.5	98.3	39.4	46.9	78.8	69.4	30.7	38.3	71.2
	Ambala	97.3	88.5	99.9	6.2	56	98.8	91.6	96.5	80.3	99.7	73.1	95.1	84.5	98.3	23	43.9	79.3	66.6	23.2	35.8	70.1
	Yamunanagar	92.9	85.3	99	3.8	66.1	96.4	88.2	91.2	70.6	98.5	77.1	89.5	74.5	98.1	15.5	17.3	60.6	53.1	14.9	47.1	66
	Kurukshetra	93.3	85.4	99	6.8	60.5	98.1	88.8	87.7	64.4	98.7	73.6	91.4	75.7	97.6	16.9	23	68.1	51.9	15.7	48.4	66.4
	Kaithal	94.9	90.5	98.8	2.1	54.1	96.5	90.4	80.9	56.8	99.1	71.8	93.2	68.5	98.2	11.1	29.1	62.9	48.9	7.9	49.8	64.5
	Karnal	94.3	90	98.2	6.6	65.2	96.3	93.5	90.6	59.5	98.7	73.6	92.6	68.7	98.1	13.6	32	63.3	51.1	18.3	39.2	65.8
	Panipat	93	88.8	99	3.8	55.8	98.3	96.6	94.9	62.5	98.8	68.5	92.2	75.2	98.3	15.7	44.9	65.4	65.7	22.6	36.5	67.3
	Sonipat	93.5	87.1	98.6	5.3	49.3	98.3	92.3	93.4	55.5	99.1	71	91.3	69.2	98.6	13.8	41.4	61.9	59.6	19.2	31.2	64.8

	Jind	94.2	83.1	97.8	6.7	58.8	96.4	86.4	85.7	59.5	98.9	74.2	89.6	65.8	98.1	10.1	23.6	56	46.9	11.3	28.6	61.9
	Fatehabad	94.9	88	98.8	7.4	59.5	98.2	87.5	87.2	59.3	98.6	75.8	89.2	72.9	97.4	12.9	14.4	60.2	45.3	11	41.4	63.9
**Haryana**	Sirsa	93.5	90.1	99.1	4.9	50.5	94.5	88.9	73.8	54.2	98.5	71.2	87.7	65.9	98.2	13.6	24.8	68.7	44.3	10.1	40.5	62.5
	Hisar	90.4	82.2	98.8	8.8	59.2	96.5	82.6	77.9	53	97.9	69.2	86.6	61.9	96.8	13	19.3	59.6	43.1	15.3	43.9	61.9
	Bhiwani	94	86.8	99.7	9.4	36	95.3	89.4	81.2	50.8	99.1	70.2	87.7	62.6	98.2	7.8	24.8	65.1	52.2	9.5	34.6	61.4
	Rohtak	89.9	79.5	97.8	6.9	49.5	95.7	80.5	86	46.4	97.5	67	90.1	67.6	96.1	11.2	25.9	54.6	52.5	16.3	33.9	60.9
	Jhajjar	93	83.6	99.2	4.1	48.3	96.7	83.4	87.9	53.8	98.3	72	86.1	67.7	96.7	10.5	16.1	63.3	54.9	12.7	28	61.1
	Mahendragarh	91.5	84.3	95.8	9.3	60.6	97.5	89.2	87.1	57.6	97.7	69.1	88	63	97.2	7.5	15.5	55.6	36.9	11.9	21.7	59.9
	Rewari	85.6	76.7	97.7	9.4	36.4	94	73.8	80.7	50.2	97.2	66.9	81.5	60.2	95.6	11.6	22.4	52.2	39.7	15.9	30.6	57.6
	Gurgaon	74.8	67.8	86.3	4.4	30.2	91.5	80.1	88.8	47	94.9	48.7	84.6	51.5	97.7	22.8	32.1	55.2	43.1	25	17.4	55.3
	Mewat	66.9	54.8	98.1	4.2	27.7	76	57.8	34.7	46.9	83.1	56.9	29.3	37.7	94.3	7.4	15	29.1	25.4	7.7	13.9	41.9
	Faridabad	86.2	77.7	92.8	10.2	57.6	92.5	87.6	89	51.3	96.6	52.5	82.6	57.3	97.6	8.6	15.1	53	35.9	11.4	10.9	56.1
	Palwal	79.1	68.2	98.7	7.3	42.7	85.8	64.1	61.7	48.6	89.6	61.3	66.7	46.6	89.5	10.7	14.7	39.2	36	12.5	27.4	51.3
	North West	73.6	59.2	79	9.3	27.3	89.9	85	94	40	98.5	47.3	87.2	62.4	98.1	16.5	16	62.2	49	18.2	25.8	55.4
	North	79.5	70.2	71.2	7.7	21.2	85.7	88.6	93.7	44.9	97.6	53.2	87.8	74.3	97.4	11.6	14.9	67.9	55.9	21.9	21	56.5
	North East	80	68.6	71.3	10.3	32.1	92.8	89.3	97.5	48	98.4	62.5	89.1	68	99.2	16.5	24.8	60.8	58.2	25.5	22.6	58.9
	East	82.7	77.9	71.9	9.4	29.3	88.5	89.4	95	56.7	98	54.9	90	71.8	97.9	19.9	20	69.5	63.9	27.4	21.7	59.9
**Delhi**	New Delhi	82.6	76.1	84.1	6.3	31	87.9	92.6	96.3	46.4	97.6	43.2	89.9	73	97.2	25.3	31.3	83	59.4	36.2	10.2	60
	Central	75.2	72.3	76.9	6.6	20.9	90.4	90.2	95.4	50.6	98.3	48.4	87.8	79.5	97.7	20.2	25.1	80.1	64.5	29.5	20	59.5
	West	86.4	80	78.7	5.6	24.4	94.6	95.5	99.1	58.1	99.3	51.5	92.4	83.3	99	25.8	27.2	85.4	66.9	35.7	32.4	64.5

	South West	74.6	61.5	87.3	10	41.5	89.6	87.5	96.4	49.4	96.9	51.4	91.7	72.8	99.5	11.4	14.6	76.9	54.6	18.1	17.2	58.1
	South	77.9	67.7	76.4	7.6	29.9	87.7	90.3	96.8	44.8	97	42	86.2	68.5	98.4	15.1	20.3	72.9	50.7	24.5	17.4	56.6
	Ganganagar	81.9	68.3	99.3	6	41.2	88.4	69.9	60.2	48.9	93.6	64.4	77.7	52.6	96.8	11.1	13.3	58.3	27.6	10.2	31.7	54
	Hanumangarh	80.3	71.7	98.4	5.2	33.9	84.7	78.3	53.7	44.2	90.6	61.9	72.1	50.5	97.8	9.8	9.5	54.4	26.5	9.1	19.1	51
	Bikaner	79.9	56.2	98.4	3.3	23.6	86.7	80	52.7	44.6	85.7	55.4	67.6	40.6	97.1	9	8.4	45.6	20.6	9.6	17.3	47.6
	Churu	77.6	45.9	98.2	3.7	20.1	80.1	74.9	43	24.7	88.2	50	63.7	32.2	95.4	4.3	16	34.8	10	6.4	9	42.2
	Jhunjhunun	86	59.8	99.2	4.9	33.8	89.8	73.3	63.9	43.8	92.9	60.3	74.3	48.3	97.2	5.8	20.1	55.1	19.2	11.4	15.3	50.9
	Alwar	75.4	59	98.1	3.4	41.7	86.5	79.9	55.9	55.2	90.7	54.2	65	42.3	96	7.7	21.1	43.5	16.8	10.2	21.9	49.8
	Bharatpur	63.1	45.1	98.1	3.7	38.8	72.5	66.3	43.9	43	82.1	38.9	51.5	30.5	91.4	3.5	14.5	31.8	10.9	6.4	14.6	41.2
	Dhaulpur	48.8	28.5	98.4	2.5	41.8	63.6	64.1	29.2	35.8	79.8	31.6	49.5	26	91.6	2.4	10.9	36	7.5	5.7	17.2	37.5
	Karauli	50.9	24.9	97.7	6.4	26.6	63.7	67.8	24.8	30.6	74.6	21.4	38.2	17.5	90.6	2.9	10.5	28.5	4.2	2.9	15.4	34.1
	Sawai Madhopur	55.2	27.8	97.1	6.1	32	73.1	59.5	26.3	40.5	78.7	31	45	19.1	91.6	3.2	8.7	29.4	4.8	4	19.8	36.8
	Dausa	62.7	32.2	98.7	3.8	39.2	72.6	82	36.9	46.8	74.4	29.3	46.4	21.6	92.3	4.4	12.5	26.3	5.2	5.4	20.7	39.7
	Jaipur	83.6	60.4	95.8	9.2	29.8	86.8	83.9	66.1	65.2	92.8	53.4	75.6	51.9	96.6	17.6	37.6	54.7	24.6	22.5	24.9	55.1
	Sikar	88.3	62.1	98.4	4.7	24.2	90.7	91.2	69.8	49.4	92.2	59.3	74.7	41.7	97.3	9.1	29.8	47.7	15.2	8.5	26.9	52.8
	Nagaur	75.8	43.3	97.9	4.6	32	89.4	64.4	36.6	46.7	85.4	54	62.7	28.4	96.3	4.3	10.6	32.3	8.4	5.9	13.6	43.2
	Jodhpur	71.4	50.3	95.7	5.3	31.9	86.5	73.4	54.7	56.5	87.4	50.4	62.8	42.7	96.8	8.3	8.7	47.9	20	11.1	24	48.1
	Jaisalmer	48	28.6	97.5	3.4	17	69.1	59.9	27.5	30	63.9	38.3	39.1	22.9	96.1	6.6	8.1	25.8	8.5	4.9	8.7	33.9
**Rajasthan**	Barmer	35.2	25.4	95.8	3.8	22.1	53.6	41.9	17.2	22	49.5	21.5	28.1	13.5	91.9	2.9	2.3	15.1	5.3	3	8.7	27
	Jalor	51.2	28.7	98.3	2.1	21.3	67.9	39.6	28.1	31.2	72.1	26.7	44.2	17.3	94.1	5	1.1	18.5	3.5	2.6	26.1	33.6

	Sirohi	55.5	30.9	98.9	1.2	24.9	73.2	56	42.5	39.9	79.2	30.1	52.7	26.3	91.6	3.5	3	21.8	7.7	5.5	22	37.5
	Pali	72.4	39.5	98.4	5.3	43.7	79.3	57.9	46	52.6	89.2	39.4	64	28.5	95.3	4.2	4.8	29.4	8.7	7.6	22	43.3
	Ajmer	80.2	58.1	97.3	2.4	29.4	89.9	79.7	64.1	64.3	96.5	53.5	79.3	47.5	95.9	9.7	5.9	50.3	22.1	11.8	31.6	52.4
	Tonk	63.2	30.9	97.4	1.2	31.5	71.8	52.5	38.3	51.9	90.9	40.7	59.7	28.4	93.4	2.5	1.5	43.4	4.4	3.7	22.7	40.6
	Bundi	55.5	31.9	95.8	1.4	39.6	73.4	49.7	32.3	50.6	83.6	38.7	53.4	24.6	91.2	5.8	3	44.7	6	4.4	21.4	39.4
	Bhilwara	58.7	33.9	96.2	1.4	30	75	63.2	43.9	63.1	92.1	45.2	59.3	28.2	95.1	5.1	4.6	40	5.9	6.6	32.7	43.5
	Rajsamand	59.2	35.7	90.7	1.5	23.4	79.8	68.2	46.2	46.9	87.9	39.1	59.1	19	91.6	3.5	1.6	19.5	9.1	4.4	13.2	38.7
	Dungarpur	55	27.5	97.2	1.3	14.8	76.2	63.3	29.6	31.8	69.7	17.7	42.5	15.5	89.8	1.7	1.1	14	4.5	2.5	17.1	32.9
	Banswara	35.2	19.9	98.6	1.5	22.1	64.4	42.2	20.7	29.3	47.7	13.6	27.1	11.8	81.1	2.7	2.3	14	5.7	3.6	11.3	27
	Chittaurgarh	46.7	31.9	93.3	1.8	45.4	69.4	49.7	41.8	59.2	90.6	37.8	55.1	24.3	93.2	7.4	5.7	34.6	10.3	7.6	24.1	40.7
	Kota	72.9	53.2	94.6	3.4	34.7	87.4	65.9	63.8	60.6	94.9	52	78.1	48.7	95	13.9	9.5	72.9	22.3	14.6	31.1	52.4
	Baran	56.7	27.1	96.5	2.8	27.1	75.2	49.2	33.5	43.4	83.4	35.2	59.3	17.6	91.5	2.7	2.2	39.2	4.4	3.2	17	37.3
	Jhalawar	43.4	28.2	97.3	1.4	32.4	66.6	40.6	32.8	41.4	86.7	31.9	47.4	14.7	91	2.4	2.9	29.6	6	3.7	18.1	35.1
	Udaipur	47.8	25.6	94.3	4.8	26.3	71.6	40.9	35.1	45.6	67.2	25.3	41	22.3	87.1	5.7	4.1	20	9.1	6.5	23.6	34.6
	Pratapgarh	34.7	16.4	96.8	1.5	30	61.7	27.9	25.8	35.2	58.9	16	34.6	11.1	87.2	1.2	2.4	12.6	4	1.9	7.4	27.4
	Saharanpur	64.4	58.7	98.6	4	62.8	78.2	72.5	74.5	37.9	87	56.2	54.9	33.7	93.6	4.1	2.1	29.4	28.2	4.6	23.2	47.2
	Muzaffarnagar	68.4	61.5	99.2	6	56	81.6	81	77.1	34.8	87.8	56	52.2	40.7	94.8	3.5	1.6	33	28.6	4.6	35.3	49.5
	Bijnor	65.7	59.9	98.6	3.4	62.5	74.7	60.8	86.1	39.6	71.7	54.1	45.1	27.4	93.9	3.1	2	22.9	19.2	3.8	15.5	44.1
	Moradabad	63.1	54.2	97.6	2.7	53.9	71.6	69	82	34.2	72.4	46.6	45.3	29.8	91.9	2.9	1.8	23.8	18.9	4.2	24.9	43.6
	Rampur	70.8	51.2	99.6	6.8	65.5	62.1	63.4	75.2	37.7	72.2	43.6	43.5	22.8	91	3	2.5	19.6	14.1	4	34.5	43.7

Jyotiba Phule Nagar	59.3	56.4	98.7	4.4	53.5	63.1	63.5	79.8	38.4	69	51	41.5	25.9	91.1	3.3	1.2	21.8	17	3	24.7	42.4
Meerut	78	70.2	97.9	3.5	55.1	87.4	82.4	87.5	47.2	93.5	60.7	72.6	60.3	96.2	8.2	8	50.8	43	14	42.4	57.2
Baghpat	76.2	67.3	99.4	9.7	57.4	80.9	81.3	77.3	42.6	86.4	60.3	60.1	48.6	94.9	7.7	3.3	39.2	41.5	8.3	43.6	53.8
Ghaziabad	74.4	68.6	92.1	3	46.7	85.1	84.1	89.4	42.3	93.8	54.4	74.5	54.8	96.7	14.4	10.8	49.3	38	18.6	31.3	54.9
Gautam Buddha Nagar	69.1	66.6	90.7	3.5	39.4	77.8	83.3	85.6	45.9	96	44.3	77.2	49.5	97.6	17	11.6	48.2	34.8	17.9	40	54.1
Bulandshahr	66	58.9	98.1	4	57.3	74	62	65.2	41.9	80.6	52	60.4	35.4	95.5	4.7	2	31.5	24.8	3.8	23.5	46
Aligarh	63.9	54.3	98.9	2.3	62.8	78.3	69.6	59.6	41.6	79.3	43.5	58.7	34.9	94.7	4.3	3.3	32.8	17.7	7	36.3	46.7
Mahamaya Nagar	61.1	51.9	98.8	4.8	67.1	74.4	69.1	51.2	40.2	79.1	44.1	61.3	31.4	93.3	3	1.8	27.7	13.4	5	35.2	45.2
Mathura	65.6	50.9	92.8	2.3	55.2	73.9	67.6	54.5	45.8	88.6	47.7	67.3	41.2	94.1	6	3.2	30.6	18.6	9.1	21.7	45.6
Agra	68.7	55.9	96.4	1.9	57.2	83.4	74.8	65.1	45.2	89.4	44.9	72.1	46.7	94.4	7.9	5.9	42	21.6	11.3	31.1	49.9
Firozabad	56.9	42.6	96.9	2.7	65.9	69	62.4	47.5	32	72.7	35.1	50.3	24.1	91.5	2.2	2.1	25.3	10.3	5.5	21.4	39.9
Mainpuri	50.4	38.7	98.7	5.2	76.1	59.5	66.5	40	30.8	65.6	32.6	41.3	16.1	91.8	3.9	1.9	17	6.3	4.5	19.8	37.4
Budaun	44.5	32.4	98.4	4.6	58.9	55.6	54.9	43.9	25.6	43.8	24.4	26.4	13.2	88.7	2.3	1.9	12.6	7.1	2.9	27.8	33.2
Bareilly	65.8	52.5	98.4	4.5	64.9	74	63.5	71.1	36.6	64.3	35.2	41.9	28.2	92.9	3.5	3.1	25.3	16.9	6	28.9	43.2
Pilibhit	59.8	39.3	98.2	3.2	69.6	64.1	60.2	50.4	26.8	48.4	26.5	28.6	13.1	91.9	3.4	1.5	13.4	7.6	3.2	21.8	35.8
Shahjahanpur	48.5	34.1	98.3	2.9	71.4	56.9	66.5	51.9	26.8	45.7	21.1	28.1	13.9	89.3	3.3	2	13.1	8.7	4.1	26.2	35.2
Kheri	40.5	22.9	98.6	6.4	74.5	43.3	47.4	33	21.6	26.5	11.1	18.8	6.5	80.5	1.3	5	5.1	2.2	2.5	17.1	27.7
Sitapur	37.5	26.2	98.3	9.4	80.1	44.8	45.2	36.8	23	23.7	13	13.7	7.5	78.9	2.3	4.2	6.2	3.7	3.1	27.2	29.1
Hardoi	46.8	31.9	98.7	6.8	80.5	64.9	66.3	44.4	24.3	34.5	17.5	20.9	7.5	89	2.9	9.4	6.5	4.2	4.4	19.5	33.3
Unnao	49.1	33.2	97.4	9.2	77.8	60.8	44.3	49.3	31.5	38.1	23	28.3	11.4	88.7	2.1	4	9.4	4.5	2.9	19.9	33.6

	Lucknow	80	67.7	92.3	5.7	71.6	81.3	87.6	86.3	61.3	90.2	41.8	76.7	57.7	96.6	11.5	22.6	57	32.5	19.6	19.6	56.2
	Rae Bareli	47.9	27.8	98.9	9	81.8	59.6	44.9	48.8	26.6	54.6	21.2	32.5	11.8	88.5	2.6	7.7	11.2	4.9	4.3	10.4	33.6
	Farrukhabad	52.7	38	97.8	8	72.6	60.5	66.6	45.2	27.6	50.8	29.1	32	14.2	92.2	2.6	1.9	10.6	6.9	3	28.4	36.6
	Kannauj	43	26.7	98	6.3	68	52.1	69.4	34.1	23.5	52.5	20.3	29.3	10.5	89.3	1.5	1.2	10.3	4.1	3.5	17.7	32.3
	Etawah	51.1	36.5	98	4.3	76.5	61.6	74.5	43.7	30.3	73.2	30.4	47.5	21.8	92.5	2.9	2.4	25.5	9.5	4.5	21.5	39.5
	Auraiya	49	33.7	98.7	5.5	82.3	63.9	71.8	41.7	25.8	59.7	28.1	36.1	16.4	92.8	3.1	7.7	16.3	8.4	4.1	11	36.5
	Kanpur Dehat	48.6	28	98	7.4	79.3	60.1	46.4	43.9	25	35.7	18.9	23.6	8.6	90	1.4	3.5	8.4	2.4	2.3	10.8	31.1
	Kanpur Nagar	79.1	65.5	93.3	6.2	75.2	86.1	90.2	81.4	50.8	82.6	41.8	71.8	51.2	95.7	7	22.4	50.8	24	14.8	20.9	53.9
	Jalaun	62.2	38.3	96.4	3.9	67	69.9	72.3	49	37.7	75.7	31	56	24.4	92.2	3.3	11	28.9	9.5	7.4	7.8	40.5
	Jhansi	64.8	48.1	96.1	2.9	60.9	74.3	58.1	59.1	45	79.6	30	64	32	91.5	5.7	8	44.5	12.9	8.1	16.5	43.7
**Uttar Pradesh**	Lalitpur	45	25.4	96.8	1.3	61.3	60	39.9	33.4	33	59.5	15	35.2	8.2	87.4	1	0.9	16.9	1.8	1.8	18.9	31.5
	Hamirpur	54.9	33.1	98.3	7	61.9	64.7	74.7	38.6	27.4	63.1	25.1	42.5	15.5	90.4	2.8	10.4	19.2	7.5	5.4	12.3	36.5
	Mahoba	51.9	34.5	97.6	6.4	60.7	63.3	55.9	42.8	28	67.7	21.8	45.4	14.8	87.8	3.5	3.5	23.6	3	3.5	14.3	35.4
	Banda	47.2	30.4	96.8	6	72.5	68.8	70.6	36	21.5	51.4	19	29.7	10.6	89.9	1.6	3.2	12.2	4.5	2.5	8.4	32.9
	Chitrakoot	36.4	15.3	97.5	3.8	63.2	49.2	48.5	28.5	17.4	49.8	13.3	25.4	7.1	84.1	1.2	4.2	12	1.4	2.1	5.2	27.2
	Fatehpur	45.3	30.4	98.4	6.9	78.8	64.8	65.8	41.8	23.4	37	19.6	22.3	8.7	88	2.1	2.9	7.7	2.5	1.6	9.2	31.7
	Pratapgarh	61.8	44.1	98.7	14.3	85.5	75.4	51.9	53.5	36.8	62.8	32.5	33.2	11.4	94.9	2.7	12.2	11.2	3.6	5.4	20.3	39.6
	Kaushambi	40.7	27.5	98.1	8.1	80.2	62.3	44.3	34.4	22.3	39	19.5	21.2	8	86.2	1.9	5.3	8.8	3	2.1	8	29.9
	Allahabad	63.7	51.9	97.9	14.1	79.8	79.1	70.6	57	42.2	70.6	34.5	50.5	28.1	94	8	11	31.8	13.1	11	23.3	45.5
	Bara Banki	45.8	33	97.5	9.6	74.5	57.8	62	47.2	27.6	38.1	21.4	24.2	11.1	84.8	2.8	5	9.4	4.3	3.3	22.8	33.6

Faizabad	51.2	44.1	96.3	12.7	82.5	73.1	84.1	56.8	30.4	57.2	26.9	33.9	10.6	89.6	1.5	11	6.6	4.4	5	19.9	38.9
Ambedkar Nagar	47.2	35.9	98.8	10	90.1	73.2	62.8	44.5	28.6	59.7	35.4	27.8	7.9	93.9	2.2	8.7	6.1	3.3	6.6	27.8	38
Sultanpur	54.1	40.4	97.8	9.9	87.4	74.7	58	50.2	31.5	63.1	30.4	36.9	10.5	92.5	2.2	9.3	10.4	3.6	5.4	19.9	38.5
Bahraich	35.9	25.8	96.8	12.1	75.7	46	48	27.8	19.1	22	11	13.5	4.6	78.6	1.4	3.7	3.6	2.1	1.6	6.3	25.8
Shrawasti	31.5	19.4	98.7	6.2	76.9	44.3	56.3	21.4	16.6	19.8	9.1	7.7	2.2	81.6	1.1	2.1	1.4	0.5	1.4	7.3	24.4
Balrampur	44.1	32.8	97.1	10.5	80.5	58.4	61.7	33.1	25.3	29.3	18.9	14.3	6.1	88.6	1.7	5.9	4.8	3.7	2.3	12.3	30.6
Gonda	46.9	32.3	98.2	15.2	84.6	58	53.1	44.3	25.2	33.6	19.1	18.1	6.5	89.9	2.4	9	5.3	3.1	3.9	26.6	33.4
Siddharth Nagar	56.7	43.5	98.5	9.8	79.1	64.9	78.1	46.3	25.5	49.3	18.7	23	5.6	90.1	2	6.7	4.1	3.1	1.7	13.1	34.9
Basti	59.5	41.5	98.5	11.1	86.1	72.2	63.3	49.8	29.2	53.1	23.1	27.6	8.1	92.4	2.6	9.2	7	4.4	4.3	30.2	38.3
Sant Kabir Nagar	56.8	43.2	98	8	80.9	68.3	68.2	47.2	26.4	50.7	27.2	26.2	5.9	92.7	1.6	6.3	4.6	3.1	4.2	22.8	36.4
Mahrajganj	64.9	50.1	97.3	9	87.3	64	75.4	45	26	38.9	20	25.7	6.8	93.4	1.8	6.8	5.1	4	2.4	21.8	36.5
Gorakhpur	67.5	56.2	87.9	9.5	77.7	71.2	77.6	66.9	34.9	63.9	33.4	46.1	18.8	94.7	5.7	18.8	13.2	11.6	8.3	20.4	43.1
Kushinagar	66.5	52.6	95.4	5.8	85.4	59.5	69	48.5	26	41.7	24.6	26.7	5.5	93.9	1.6	8.6	3.7	2.6	3.3	18.3	36.1
Deoria	68.9	53.9	96.1	10.1	83.9	73	68.4	58	29.5	57.8	31.1	38.4	10.1	95	1.8	12.9	8.9	5.7	6.2	20.6	40.5
Azamgarh	55.5	45.8	93.7	9.1	81.8	74.1	85.6	47.7	31.8	68	40.8	40.4	13.5	93.4	2.2	13.9	11.5	9.6	6.4	17	40.9
Mau	57.1	41.8	96.2	6.5	80.8	73.4	64	52.3	23	70.9	36.9	39.2	8.9	95.3	2.5	9.5	8.8	3.2	4.8	18.6	38.7
Ballia	60.6	46.4	96.7	9.8	79.3	73.2	62.3	51.7	21.6	56	31	35.6	8.4	93.5	1.3	8.4	6.9	3.7	5.2	14.7	37.2
Jaunpur	55.6	39.8	99.8	15.2	87	75.5	64.8	46.3	34.6	67.9	30.6	42.9	11.4	94.5	3.6	15.3	10.8	3.6	5.3	23.6	40.6
Ghazipur	51.8	38.7	98.4	10.2	85.3	71.9	60.2	41.4	27.1	54.9	31.2	35.8	10.3	94	2.9	10.9	10.1	4.8	4.5	15.6	36.9
Chandauli	60.2	44.4	97.9	7.7	81.8	69.4	66.3	44.3	28.6	56.9	31.3	44.2	14.5	92.3	3	12.1	14.4	5	6.4	18.5	38.9

	Varanasi	58.4	53.8	85.6	8.8	79.9	83.7	86.2	68.2	38.9	81.4	38	63	29.7	95.4	4.8	22.6	29.2	13.4	11.4	26.4	47.9
	Sant Ravidas Nagar (B	50.6	37.8	97.5	8.3	80.2	75.3	63.8	44.9	26.3	70.5	25.6	43.9	11.1	92.2	2.1	13.5	13.8	2.7	5.7	16.6	38
	Mirzapur	48.5	36.2	96.3	9.1	85.2	68.9	63.9	37.5	25.7	53.3	22.4	41.2	10.8	90.5	2.2	3.7	13.6	2.9	3.5	17.1	35.7
	Sonbhadra	57.5	40.4	96.3	11.9	72.7	61.4	64.4	37.4	29.9	42	23.6	34.7	18.4	85.4	7.7	10.4	20.1	12.3	10.3	17.6	36.8
	Etah	54.6	40.8	98.7	6	66	62.3	68.8	42.3	31.5	54.9	32.2	35.2	16.2	92.8	3.6	1.7	15.7	8.9	4.5	28.1	37.8
	Kanshiram Nagar	49.7	35.6	98.7	3.7	68.5	58.4	49.6	35.3	28.1	43.4	29	25.7	11.8	91.2	2.1	1.5	12.4	6.4	3.9	40.5	35
	Pashchim Champaran	65.8	34.5	69.6	9.6	78.2	38.7	30.3	22.4	15.7	28.4	14.8	16.9	3.3	86.8	1.1	7.8	1.2	1.9	3.3	5.5	25.8
	Purba Champaran	76	39.1	80.9	10.3	71	38.1	28.2	22.3	13.6	24.3	12.8	11.3	1.6	87.2	1.1	3.1	0	0.6	1.1	5.6	25.4
	Sheohar	71.1	29.2	70.7	14.7	61.1	35.2	23.7	16.8	10.6	29.1	10.2	11.9	1.1	88.1	0.9	2.3	0.8	0.5	0.8	6.2	23.4
	Sitamarhi	66.1	28.2	66.6	15.2	63	37.5	25.2	17	9.1	26	9.9	12.6	1.3	91.3	0.9	1.4	0.4	0.2	0.8	2.5	22.8
	Madhubani	70.9	31.3	78.6	10.9	63.7	48.3	26.8	21	13.5	37.6	12.7	14.1	1.2	90.5	0.6	3.5	0.3	0.3	1.8	10.5	26.1
	Supaul	67.6	30.5	84.3	9	68	36.2	14	11.4	12.4	33.5	7.6	11	1.3	89.1	1.1	3.5	0	0.1	0.8	4	23.3
	Araria	72.8	37.7	66.8	12.4	69.1	46.2	23.3	14.1	14.1	25.5	6.3	9.7	1.4	88.9	0.5	2	0	0.7	0.7	4.7	23.9
	Kishanganj	86	42.7	87.2	6.8	70.3	59.5	26	15.8	14.8	36	3.3	13.7	2.6	86.1	0.5	2.6	0.4	0.8	0.9	5	26.9
	Purnia	76	42.2	88.2	14.4	66.2	52.1	32.3	18.5	14.4	35	9.1	17.4	4.1	86.2	1	5.6	0.5	1.7	2.3	6.8	27.7
	Katihar	80.7	43.5	90.5	9.1	73.9	52	25.7	19.8	11.1	25	9.3	13.8	2.6	87.7	0.4	3.3	0.6	1.1	0.9	6.4	26.8
	Madhepura	73.3	37	86.5	8	66.2	35.7	22.2	14.8	11.1	30.8	7.8	11.9	1.2	88.7	1	3.2	0.7	0.9	1.4	4.9	24.4
	Saharsa	62	34.6	86.2	8.3	65.5	40.5	38.6	20.3	11.7	37.5	13.2	14.8	2.5	85.4	0.7	4.5	0.9	1	2	3.4	25.6
	Darbhanga	69.9	32.4	85.7	10	62.7	49.4	32.7	25.8	12.3	40.1	12.1	16.3	2.5	90.2	0.8	7.2	0.7	1.5	1.8	2.7	26.6
	Muzaffarpur	79.6	47.9	94.3	11.5	72.6	52.2	46.7	29.6	19.8	49.4	14.1	25.3	4.8	90.8	3	7.8	2.6	3.2	3.7	6.9	32

**Bihar**	Gopalganj	69	42.5	93.4	15.8	80.7	55.2	48.9	30.1	22	35.7	21	20.9	3.2	93.8	1.4	8.8	1	2	3.4	4.3	31.3
	Siwan	77.7	49.3	90.9	25	79.9	70.4	40.4	43.6	23.6	34.3	26.2	20.3	2.1	92.9	1	8	0.3	1.6	2.7	5.2	33.4
	Saran	73	39.5	83.6	14.3	75.9	59.8	32.6	32	18.4	38.2	19.9	24.7	3.6	90.7	1.4	5.1	0.8	1	2.5	12.8	30.6
	Vaishali	77	45.1	94.2	17.2	76.6	60.3	43	34.8	19.1	41.9	16.4	22.7	4.5	91.7	2	7.8	1.1	2.1	2.9	6.9	32.1
	Samastipur	61.7	34.2	86.9	8.7	65.9	43	36.9	21.6	12.4	26.4	11.4	13.5	2.2	85.4	1.4	5.7	1.1	1.4	2.4	5.1	25.4
	Begusarai	62.7	32.4	97	7.2	64.8	50.2	23.9	23.6	13.4	48.7	12.7	18.5	4.1	91	1.8	8	1.7	1.7	2.6	4	27.3
	Khagaria	64.1	33.2	93.5	8.7	52.2	48.1	26.1	22.9	13.6	41.6	11.6	16.8	3.6	91	1.2	6.5	0.5	1.4	2.6	4.3	26.1
	Bhagalpur	75.8	44.8	94.9	9.8	59	67.6	34.7	32.7	15.3	59.4	13.5	28.5	7.7	90.9	1.2	5.6	1.8	2.3	3.6	6	31.5
	Banka	68.7	30.7	97.2	8.7	66.8	54.3	19.5	18.6	13.4	34	9.4	13.7	2	89.9	0.7	2.8	0.9	0.9	1.8	4	25.8
	Munger	75.6	38.3	94.4	12.3	65.8	71.3	34.9	39.5	18.5	61	16.5	34.2	7.4	89.3	0.9	9.4	1.9	1.7	3.1	6.3	32.8
	Lakhisarai	65.2	32.6	93.9	6.2	53.1	60.6	29.5	29.9	12.1	66.2	16.8	30.9	5	88.5	1.1	6.1	1.2	1	2.2	6.1	29.3
	Sheikhpura	71.3	35.3	86	8.3	56.7	65.3	34.6	29.1	13.2	63.4	17	26	2.9	93.1	1.3	3.6	0.9	0.4	2.2	6.7	29.7
	Nalanda	73.9	47	89.6	7.5	54.5	65	39.2	32.9	13.3	62	14.8	28.1	5.6	92.2	1.4	4.3	1.5	1.9	2.8	10.1	31.3
	Patna	77.7	57.8	92.1	7.2	59.3	74.4	55.4	59.3	29.6	80.9	25.1	58.8	22.7	93.6	5.3	15.8	13	12.1	11.7	10	41.5
	Bhojpur	76	48.1	98.5	7.5	70.7	67.2	31	45.2	19.9	52.7	23.4	26.9	6.2	95.9	0.9	5.2	3.6	2.2	3.1	4.6	33
	Buxar	68.1	48.5	97.2	7.9	70	65.2	36.7	54.6	20.2	58.2	27.5	33.1	8	92.3	1.4	10.4	5.3	3.1	5.8	8.2	34.7
	Kaimur (Bhabua)	62.6	47.7	96.7	8.9	60.8	59.7	35.4	36.8	18.5	60.4	23.3	28.3	6.2	90.6	0.9	5.7	6.7	2.7	3.2	10.8	32.2
	Rohtas	70.2	46.3	98.9	9.5	65.6	67	30.7	52.2	16.6	62.5	26.4	28.1	4.8	92.5	1.4	11.1	5	1.2	3	11.6	34.1
	Aurangabad	74.1	52.1	93.4	7.9	67.4	63.8	41.7	35	16.2	48.7	22.1	27	5.2	92.9	1.7	4.5	3.6	1.4	1.3	8.5	32.2
	Gaya	57.8	32.2	97.6	7	59	57.5	22.2	25.8	16.5	48.2	16.6	21.7	4.7	90.1	1.5	10	3.1	1.5	3.1	8.6	28.2

	Nawada	64.9	33.6	96.2	9.4	39.3	59.1	33	28.9	11.2	43.5	16.5	20	3.7	93.1	0.7	2.8	2	2	1.4	7.4	27.4
	Jamui	62.9	25.8	97.1	8.2	61.8	56.1	26.4	15.3	15.2	36.1	12.3	19.1	2.9	91.1	0.7	2.4	0.5	0.5	0.9	3.6	25.8
	Jehanabad	65.7	40.1	92.9	6.9	50.5	57.4	36	33	13.9	54.4	18.9	25.6	6	88.5	1.3	3.4	2.9	2.2	2.7	13.3	29.9
	Arwal	63.9	35	96.9	5.2	61.1	53.6	20.8	28.7	11.2	36.2	15	12.3	1.3	89.4	0.7	2.6	0.8	0.9	1.1	5.7	26.1
**Sikkim**	North District	97.1	96.6	98.8	5.6	1.1	88.3	98.4	96.6	1.6	14.8	1.8	85.3	19.5	95.7	13.9	3.7	0.2	5.6	9.1	0.2	39.7
	West District	98.2	96.9	99.1	4	1.3	84.3	99.6	96.7	1.2	16.1	2	84.9	10.2	95.7	9.4	2	0.3	2.9	6.1	0	38.6
	South District	99.2	97.9	99.4	7.4	1.7	87.7	99	98.2	3.4	34.4	2.8	93.3	16.8	96	12.6	4.8	0.4	8.9	10.3	0.2	41.6
	East District	97.5	98.1	98.3	7.9	1.1	80.4	96.8	96.7	3.2	29.2	4.3	88.7	30.1	95.4	14.8	11.3	0.8	12.9	20	0.2	42.3
**Arunachal Pradesh**	Tawang	63.8	68.1	79.9	20.2	1	55.2	68.6	75.1	6.5	9.1	2.5	59.5	5.8	75.9	10.2	4.6	0.5	4.9	2.9	0.5	29.3
	West Kameng	78.2	81.4	79.5	6	8.9	74.6	79.1	83.2	13.5	29.5	4.6	75.7	19	83.3	15.1	9.6	0.4	7.4	7.2	1.1	36.1
	East Kameng	53	53.6	47.4	4.2	5.9	35.3	23.6	49.9	18.9	29.8	4.5	37.4	12.8	57.7	5.7	7.5	0.3	2.9	4.1	0.3	21.7
	Papumpare	89.6	89.3	90.3	5.4	13	71.5	84.6	90.9	38.5	85	7.9	78.6	47.1	95	28	30.6	4.4	11.7	25.4	2	47.2
	Upper Subansiri	58	59.4	58.2	3.2	2.8	56.6	64.1	56.7	24.1	41.5	1.9	63.5	17.6	68.2	8.3	8.5	0.7	3.4	6.4	0.2	28.7
	West Siang	88.9	89.7	86.7	9.5	13.8	74.5	78.6	83.8	36.4	73.6	4.7	71.9	32.7	85.5	17.5	8.2	2.1	6.8	9	1.8	41.8
	East Siang	87.9	88	85.4	7.1	18.2	71.9	85	77.1	48.4	67.6	2.3	80.3	23	94	16	13.9	0.7	3.7	8.5	2.7	42.1
	Upper Siang	69	73.2	78.8	8	7.5	70.9	75.1	53.6	38	41.5	1.5	57.5	13.2	76	8.9	2.8	0.3	2.5	3.6	0.2	32.5
	Changlang	81.1	79.4	82.9	2.7	52	80.1	80.8	47.4	28.1	61.2	6	58.7	14.8	90.3	8.5	18	0	2.7	4.3	2.4	38.3
	Tirap	88.2	83.9	91.9	1.9	3.6	82.1	74.1	43.1	26.4	51.1	3	61.1	8.4	88.8	5.2	18.2	0.3	1	2.7	1.2	35.1
	Lower Subansiri	76	79.2	78.8	7	8.3	60.6	80.4	81.7	28.6	26.5	5	79.9	19.5	88.7	24.3	24.2	0.4	14.6	13.9	0.8	38
	Kurung Kumey	55.5	55.2	59.3	4	2	39.8	61	61.1	22.3	25.1	1.2	55.7	9	65.6	10.1	2.9	1.1	1.6	1.8	0.4	25.5

	Dibang Valley	75	76.9	85.5	0.9	4.9	78.5	75.8	68.3	41.7	18.3	1.2	62.5	8.2	70.8	10.8	1.7	0	4.9	4	0.4	32.9
	Lower Dibang Valley	76.6	79.5	83.8	10.6	39.9	68.7	72.9	70	33.8	50	3.7	63.3	14.1	93.7	11.6	22.3	0.2	4	7.9	6	39
	Lohit	68.1	67.7	83.5	2	64.8	66.1	79.9	45.7	24.9	59.2	6.8	56.4	13.4	83.3	7.1	22.3	0.8	1.1	5	4.8	36.5
	Anjaw	57.3	56.7	59	5	3.4	42.3	72.9	57.4	29.3	16.4	0.2	61.7	2.5	73.8	4.6	2.1	0.1	0.2	0.7	0.1	26
	Mon	68.4	77.6	93.8	8	4.5	58.1	70.5	38.3	9.5	21.4	8.7	25.6	8.2	87.2	6	11.8	0.2	0.9	3.7	0.6	28.7
	Mokokchung	97.3	95.2	99.3	31.2	6.1	84	92	87.5	22.2	49.4	49.3	73.6	43.6	94.3	22.3	23.7	1.4	18.1	16.5	5.2	48.4
	Zunheboto	79.8	88.3	96.5	12.6	1.9	54.5	85.6	86.2	6.6	8.5	16.5	47.4	3.4	89.8	13.3	19.8	0.4	3.3	4.6	0.7	34.3
	Wokha	86.4	90.9	98.9	20.6	8.1	78.4	71.5	73.1	10	24.8	26	64.5	20.4	91.3	15.5	26.1	0.2	5.6	13	3.1	39.6
	Dimapur	87.2	88.6	96.8	6.4	24.5	80.2	89.2	80.4	17.4	92.4	26.1	80	55	95.8	21.2	40.3	4.7	13.5	22.6	16.9	50.3
**Nagaland**	Phek	84.9	84.2	97.8	9.1	2.7	66.7	86.8	64.1	3.3	4.7	13.5	38.2	3.5	88.3	9.5	21.3	0.1	4.7	8.4	0.1	33
	Tuensang	81.3	76.9	98.2	11.2	1.8	75.8	76.1	61.9	9.5	7.8	22.5	43.8	7	88	9	13.6	0.3	2.6	5.6	0.5	33
	Longleng	75.8	74.3	96.6	12.8	2.5	66.2	73.7	54.5	11.8	11.2	14.3	36.2	5.7	89.8	4.8	11.5	0	0.6	4.6	0.6	30.9
	Kiphire	81.9	85.1	98.5	11.2	2.1	59.3	82.1	68.1	5.7	16.6	15.8	36.7	6.2	90.6	9.3	19.8	0.2	2.7	6.8	0.5	33.3
	Kohima	89.5	92.4	98.4	18.9	4	78.8	88	82.2	8.3	11.1	31.7	72.7	26.4	96.1	29	41.4	0.8	21.7	28.2	2.6	44
	Peren	72.4	84.6	96.6	5.8	12	69.7	77.1	56.8	11.9	44.5	14.9	55.1	15.3	89.8	14.8	23.9	0.6	1.9	8.8	3.3	36.3
	Senapati (Excluding 3	75.9	80.8	97.2	26.6	21.1	72.5	91.6	69.9	13.4	17.5	6.9	60.3	10.3	94.2	5.6	6.1	0.1	9.7	6.7	5	37
	Tamenglong	69.2	78.5	96.3	28.2	6.5	66.5	78.8	65.4	9	18.8	6.6	45.3	7.4	85	5.4	2.4	0.5	3.1	6.8	3.6	32.7
	Churachandpur	89	90	98.9	19.2	27.8	81.6	93.5	78.6	29	33.1	12.5	64.2	19.9	88.8	8.3	7	0	19.1	11.4	8.7	42.3
	Bishnupur	80.4	86.2	99.4	56.6	67.3	84.7	99	87	29.9	57.9	13.5	75.5	21.9	96.3	11.5	7.6	0.2	11.8	8.5	15.9	48.9
**Manipur**	Thoubal	80.1	86	98.7	53.5	73.6	85.3	98.4	87	34.7	51.7	11.4	62.2	18.3	95.8	10.6	5.5	0.5	10.8	7.5	20.8	48.2

	Imphal West	87.6	90	99	57.9	63.5	87.7	97.6	94.1	53.1	65.8	23	82.3	35.3	97.1	19	11.5	1.8	28.6	18.9	26.3	55.6
	Imphal East	81.4	84.7	98.6	43.7	51.4	83.6	97.3	87.6	44.9	64.9	15.9	72.7	29	95.7	15.3	12.7	0.4	20	14.5	21.7	50.4
	Ukhrul	62.6	79.8	94.2	26.9	5.1	67.3	91.6	65.1	9.1	4.7	5.2	40.4	2.7	88.7	6.9	3.1	0.3	3.2	5.8	2.3	31.8
	Chandel	75	80.3	94.1	29.7	21.9	73.4	86	68.8	32.1	32.3	5.9	59.2	18.4	92.2	10.2	7.8	0.5	10.3	8.8	10.6	39.4
	Mamit	66.3	73.9	84.9	19	5.1	76.5	73.5	78.6	20.7	47	26.2	60.8	44.1	84.7	6.2	24.4	0.7	22.7	7.7	5	39.7
	Kolasib	82.8	88.8	95.9	14.4	7.9	88.9	86.9	96.8	26.9	75.9	40.4	80.1	66.2	93.6	9.9	44.8	1	43	18.7	8.5	51.4
	Aizawl	90.8	96.4	99.5	27.9	16.3	93.5	94.7	98.4	45.1	49	54.5	90.9	77.3	99.1	18.3	49.3	3.8	61.5	43	13.9	58.9
**Mizoram**	Champhai	79.4	88.4	98.7	8.8	7	81.4	87.1	99	27	17.4	38.1	83.3	53.9	93.5	9	24.9	0.9	49.5	16	4.4	46.3
	Serchhip	85.4	94.5	99.5	8.8	8	89.9	92.8	99.5	33.3	33.3	41.8	85.6	63.7	95	9.2	51.2	0.3	51	15.3	6.4	51
	Lunglei	79.9	86.2	95.9	14.2	6.1	85.1	81.8	95.5	23.1	46.9	38.3	79	58.5	92.8	10.1	34.8	0.9	38.7	20.2	5.8	47.6
	Lawngtlai	66.5	70.9	75.2	9.1	5.6	74.8	58.2	72.1	14.2	42.5	15.6	54.6	33	74.6	5.2	10.1	0.3	17.5	10.3	2.5	34.1
	Saiha	88.1	90.8	98.5	5.9	1.9	87.8	81.5	99.5	11.9	30.7	31.1	75.3	48.3	90.2	8.8	27	0.3	38.2	15.1	1.7	44.5
	West Tripura	93.1	87	92.8	4	53.5	89.3	85.8	41.2	23	92.3	8.3	76	27.1	92.4	4.2	8.9	1.9	2.8	9	14	43.8
**Tripura**	South Tripura	93	79.5	90.3	2.9	58.8	82.4	72.4	23	13	85.1	4.4	58.4	14.9	85.3	2.6	3.6	0.5	1	2.6	7.5	37.6
	Dhalai	85.8	71.3	84.4	0.5	48.5	78.2	58.5	17.6	8.7	79.6	4.3	54.4	10.5	82.9	1.5	3	0.4	0.2	1.5	6.9	33.6
	North Tripura	89	81.7	89.7	3.1	43.4	74.5	67.9	27	15.3	78	6.8	51.7	15.7	85.9	5.1	2.6	0.4	0.4	2.7	6	35.8
	West Garo Hills	96.7	93.6	92.9	13.9	47.9	84.2	84.3	55.1	26.2	67.2	8.5	60	10	88.4	11	7.3	0.5	2.3	8.6	7.1	41.6
	East Garo Hills	96	95	75.4	11.6	52	80.6	69.2	52.1	22.3	53.8	7.4	54.5	5.7	77.8	5.8	3.1	1	3.1	4.6	5.5	37.2
	South Garo Hills	99.8	99.7	77.8	13.1	20.8	96.9	78.6	76	55.4	87.4	11	88.8	24.3	97.5	21	3.1	0.6	3.2	8.1	7.6	46.6
**Meghalay**	West Khasi Hills	82.5	88.3	93.9	10.2	8.3	72.9	79.1	27.9	5	13	7.4	52.2	2.2	90.5	6.8	0.9	0	0.6	1.4	0.7	30.7

	Ribhoi	80.3	81.3	87.8	6.4	8.6	61.6	76.2	33.3	6.4	24.9	8.9	47.1	6.5	86.4	6.6	3.2	0.3	1.4	3	1.6	30.1
	East Khasi Hills	84.2	86.5	95.9	10.3	5.4	75.2	89.4	68.3	9.9	11.1	16.7	68.1	18.4	92.8	16.3	15.3	0.6	7.6	17.3	3.6	37.9
	Jaintia Hills	75.9	84.7	96.6	3.9	1.7	77.4	85.8	43.3	1.5	11.3	7.3	50.1	11.8	90.6	14	3.3	0	1.6	5.1	4.2	32.1
	Kokrajhar	92.2	88.3	92	2.5	79.2	59.3	76.4	31.4	15.6	53.2	3.3	31.5	3.3	88.7	2.9	6.7	0	1	3.1	8.8	35.6
	Dhubri	93.9	85.8	86.5	3	59	48.7	70.2	21.4	13.5	52.6	4.2	24.2	6	84.1	2.6	4	0.3	1.4	3.8	4.4	32.1
	Goalpara	96.4	92.1	91.7	4.3	65.3	61.2	85.5	36.6	18.4	57.5	9.3	39.6	8.5	83.7	5	7.6	0.4	1.4	3.8	12.8	37.8
	Barpeta	95.9	92.4	93.1	3.1	74.2	62	75.6	39.2	19.8	58.6	6	32.3	6.4	91.6	2.4	9.3	0.1	1	4.5	3.7	36.9
	Morigaon	93.2	88.7	96	4.5	74.8	62.3	69.3	32.6	16.4	60.4	4.8	35.9	6.6	84.8	4.2	9	0.3	1.1	3.5	9.5	36.5
	Nagaon	94.7	91.7	96.6	2.7	75.1	71.5	85.1	37.6	15.5	68.1	9.3	41.3	12.3	87.4	4.6	8.2	0.4	1.9	4.3	7	39.2
	Sonitpur	96.7	92.4	96.6	2.5	84.8	76.3	86.2	41.2	20.3	63.2	8	50.7	11.3	89.2	4.1	8.4	0.9	3.2	5.1	8.2	40.8
	Lakhimpur	89.9	86.5	90.9	5.9	75.5	73.2	83.3	46.6	25.9	58.7	9.2	42.9	9.1	86.7	7.1	10.5	0.5	2.3	6.2	5	39.1
	Dhemaji	86.4	82.8	89.5	7.9	78.8	65.6	71.2	31.7	17.2	43.8	7	32.6	4.5	85.9	4.5	10	0.1	0.9	3.9	5.2	35
	Tinsukia	86.7	78.9	90.4	3.4	71.4	79.4	71.3	43.2	19.6	57.4	15.2	54.6	14.2	73.8	7.7	18.3	0.8	6.2	7.8	14.3	39.5
	Dibrugarh	93.8	89.3	92.7	6.5	74	75.9	88.8	49.7	22.3	63.4	14	52	11.8	82	7.7	11	1.5	5.5	7.6	4.8	40.9
	Sivasagar	94.3	90.4	94.5	5.2	76.8	83.3	77	53	26.5	64.4	13.6	55.6	15.9	82	9.5	16.2	0.8	4.7	8	9.1	42.4
	Jorhat	94.3	93.3	94.4	8.1	77.2	81	89.9	59.5	35.9	76.3	15	63.4	17.5	89.6	13.4	21.7	3.3	8.1	12.8	5.4	46
**Assam**	Golaghat	96.4	94.4	96.7	4.5	81.4	81.5	93.6	48.3	24.6	74.3	11.4	57.2	10	87	7.6	11.1	0.3	2.3	4.4	5.8	42.8
	Karbi Anglong	89.9	83.9	94.8	1.6	73.5	70.4	78.3	32.3	15.4	57.8	5.6	48.3	7.5	85.9	3.7	5.7	0.3	1.2	3.4	2.5	36.4
	Dima Hasao	81.9	83.5	91.8	2.8	12.8	68.1	75.7	43.8	11	50.2	12.2	46.1	10.7	82.9	4.9	15.2	0	2.4	5.7	1	33.5
	Cachar	95	90.6	95.1	6.8	44	85.1	79	49.6	15.4	61.7	6.6	42.8	14.8	86.4	3.2	12.4	0.2	2.4	7.4	3.5	38.3

	Karimganj	94	85.4	94	3.7	40.7	77.4	83.7	48.5	10.3	63.1	3.4	31.5	7.8	92.2	2.3	6.1	0.5	2.3	4.1	4	36.1
	Hailakandi	90.4	79.3	89	5.2	43.6	73.1	67.8	41.3	7.2	47.9	1.9	22	7.2	89.6	1.6	5	0.1	1	3.9	1.9	32.4
	Bongaigaon	96.4	94.3	91.9	4.1	80	66.2	83.4	40.6	21.5	70.1	8.4	42.7	7.3	90.9	3.7	9.8	0.2	2.7	5.6	8.8	39.9
	Chirang	93.7	92.6	95.3	2.3	85.6	69.4	76.4	31.8	17.7	48.7	5.5	31.2	3.5	89.6	3	6.7	0	0.4	2.8	6.9	36.7
	Kamrup	96.8	94.3	93.6	8.2	77.4	64.1	88.5	49.3	20.9	79.9	11.7	48.2	8.1	89.3	4.6	10.9	0.1	1.1	5.1	8.1	41.3
	Kamrup Metropolitan	93.9	93.4	98.2	11.6	36.7	82.7	95.3	84.9	39.1	92	21.6	80.4	39.6	95.5	21.4	39.9	8.6	17.3	26.9	14.1	52.7
	Nalbari	97.4	94.9	94	5.7	84.3	65.3	85.1	55.2	21.5	72.2	8.9	48.9	6.3	94.7	4.3	9.1	0	0.9	3.9	8.1	41.4
	Baksa	97.7	94.5	96.1	3.6	87	70.1	90.8	43.5	19.6	61.6	6.6	44.1	4.4	91.2	2.9	4	0.2	0.8	3.7	7.5	39.9
	Darrang	94.9	90.7	93.6	2.2	81.2	64.2	74.7	33.7	14.7	55.7	5.4	31.2	4.8	90	2.4	6.4	0	0.3	3.6	6.7	36.3
	Udalguri	96.8	94.2	96.4	3.4	90.8	63.1	85.2	32.1	17.9	58.2	5.7	42.6	2.6	90	2.3	4.1	0.1	0.9	2	6.5	38.2
	Darjiling	90.7	92.3	93.4	8.3	42.3	80.7	86.8	71.3	18.1	54.8	6.6	79.7	29	89.5	4.6	11.1	2.4	7.9	12.1	8.8	42.8
	Jalpaiguri	88.2	72.2	81.9	4.1	77.3	73.2	68.8	32.3	18.4	66.8	5.7	49.9	11.4	77	2.3	4.9	0.4	0.6	4.2	4	35.6
	Koch Bihar	89.1	67.1	59.3	2.8	81.3	67.9	69	25.1	12.4	74.5	2.3	42.1	5.4	86.3	0.8	5.4	0.7	0.9	2.2	12.8	34.3
	Uttar Dinajpur	83.6	55.5	69.7	3.3	78.4	64.8	49.3	18.6	17.9	73	3.7	34.7	8	83.3	1.8	6.5	1.2	2	3.7	10.8	32.4
	Dakshin Dinajpur	81.2	67.7	75.9	1.3	76.5	74.4	59.3	22.8	14.6	79.9	3.1	55	5.7	84.6	1	2.1	0.1	0.9	2.2	6.9	34.4
	Maldah	86.4	57.2	81.3	2.3	79.1	75.3	50.2	26.3	11.6	81.6	4.7	44.7	5	86.1	0.1	3	0.7	0.6	1.5	7.6	33.9
	Murshidabad	68.5	44.3	81.5	2.4	79.9	76.1	51.9	32.3	12.4	89.1	3.8	48.2	8.5	84.4	1.2	2.7	0.8	0.6	2.2	7.2	33.6
	Birbhum	65.7	45.5	65.5	4.3	77.1	81.2	50.5	26.3	15.1	88.4	3	50.3	9.4	75.9	0.9	2.5	0.5	0.3	2.4	9.6	32.6
	Barddhaman	67.5	51.7	74.5	4.5	79.8	79	56.9	39.5	24	91.6	9	63.3	20.6	85	3	10.4	3	4.8	6.4	12.2	38
**West Bengal**	Nadia	83.3	65	73.5	5.8	85.7	81.6	65.4	41.5	18.7	92.5	10.4	63.6	14.3	91.4	1.5	5.6	1.1	2	4.6	16.4	40

	North Twenty Four Par	84	67.1	91.2	9.8	80.6	85	73.2	64.5	24.4	94.8	16.8	72.5	33.7	91.5	2.8	12.3	6.8	6.8	11	16.1	45.7
	Hugli	68.9	56.9	83.5	8.8	81.9	83.5	67	55.6	23.2	93.8	8.2	69.4	21.1	88.8	1.5	9.2	1.5	3.4	7.6	15.3	41.2
	Bankura	69.7	45.9	56.5	3.8	82.9	75	49.4	30.1	18.8	79.1	2.9	45.9	9.9	84	1.2	4.4	0.5	0.5	3.7	16.8	33.2
	Puruliya	48.4	27.7	58.9	2.5	76.8	62.1	26.7	15.5	15.2	56.2	2.9	29.4	5.7	76.3	0.6	3	0.3	0.4	1.5	6	24.9
	Haora	85.6	73	77.3	11.4	74.6	93.4	78.9	65.1	25.8	96.7	9.2	75.6	28.4	95.9	3.2	12.4	5.6	7.2	11.9	16.1	45.9
	Kolkata	82.6	73	92.8	5.5	34	90.6	87.5	89.7	21.1	99.4	17	90.2	58.8	97	6.3	15.6	19.5	22.7	19.3	15.2	50.1
	South Twenty Four Par	77.4	59.4	84.2	8.3	66.4	84.1	57.4	29.3	8.8	80.2	7.1	46.8	14.6	87.1	1.2	5.8	1.4	1.4	5.5	7.6	35.3
	Paschim Medinipur	77.8	52.4	75.8	3.7	87.1	78.4	56.3	27.5	22.5	87.4	2.9	53.4	11.6	83.4	1.2	4.8	1.7	0.9	2.7	12.1	36
	Purba Medinipur	86.3	58.3	90	8.5	87.3	81.9	55.7	30.2	19.6	94.6	5	61.7	9.1	91	0.8	3.5	1	0.6	3.4	7.8	38.3
	Garhwa	73.1	19.8	94.9	2.9	62.8	48.4	30.5	14.8	15.7	24.6	13.5	11.5	1.7	87.3	1.1	0.9	1.7	0.5	1.2	9.6	25
	Chatra	63.5	22.7	97.5	4	63.9	52.8	21.3	17.1	20.1	24.5	14.7	13.9	3.4	87.4	1.3	0.8	1.4	2.3	2	16.4	26.1
	Kodarma	80.9	38.2	96.5	2.2	70.1	73.6	60.6	49.7	41.6	76.8	18.9	43.9	16	93.4	3.4	14.1	17.7	7.2	5.4	12.3	39.8
	Giridih	77	27.9	95.1	1.7	61.9	73.2	39	28.2	31.4	61.6	12.6	34.3	6.8	86.9	1.1	6.3	5	2.3	2.1	6.6	31.8
	Deoghar	69.7	40.5	98.1	2.2	73.8	64.9	34.8	28.1	28	59	12.9	36.7	10.6	86.2	3	14.5	5.7	4.8	5.7	13.7	33.6
	Godda	62.6	19.5	96.6	1.6	71.1	49.6	30.8	15.1	17.3	45.5	7.7	17.2	3.3	82.1	0.6	2	2	0.4	0.8	3.9	25.4
	Sahibganj	70.9	35.1	95.3	0.6	61.2	61.7	23.5	18	9.9	55.2	8.4	23.1	5.8	76.7	0.4	4.7	0.9	1.9	2.3	2.8	26.7
	Pakur	69.4	18.8	94.7	1.2	71.7	62.1	22.1	15	15.4	46.6	5.9	22.3	2.4	74.3	1	2.1	1.7	0.8	0.9	3.6	25.5
	Dhanbad	78.8	52.1	95.3	1.1	62.9	83.5	45	37.7	36.3	87.9	18.1	64.6	20.7	88.9	3.6	12	10.5	10.2	7.4	8.2	39.7
	Bokaro	80.2	45.7	97.1	1.4	70.8	80	48.8	43.8	35.6	74	19.8	52.7	21.1	90	4.2	7.9	16.6	10.4	8	4.5	38.9
	Lohardaga	81	33.1	72.1	2.8	78	73.6	21.1	27.8	24.7	37	11.7	26.9	6.5	83	2.1	2.5	3.7	3.7	3	13.1	29.5

**Jharkhan**	Purbi Singhbhum	70.8	51	96	4.6	66.1	77.5	50.2	50	40.5	76.7	19.7	57.9	27.1	85.9	9.9	9.6	24.5	17	12.3	4.4	40.7
	Palamu	61.9	30.8	93.5	4	53.3	53.5	18.9	24.8	16.7	38.1	16.3	20.1	6	82.7	0.8	1.8	4.2	2.1	1.9	10.8	26.3
	Latehar	63.3	18.5	83.8	2.1	65.1	59.8	16.9	15.8	15.4	24.5	8.1	15.3	3.2	77.9	2	1	2.2	1.8	1.2	4.6	23.2
	Hazaribagh	83.3	42.6	97.6	2.6	62	78.1	51.5	42.2	37.6	75.6	15.3	49.5	10.6	90.7	1.4	11.1	9.8	4.1	3.4	11.1	37.7
	Ramgarh	81	53.7	94.3	2.4	53.5	81.3	41.6	41.3	37	81.4	19.8	59.2	18.7	90.4	4.2	5.1	9.9	6.6	6.1	13.1	38.7
	Dumka	64	21.9	96.7	1.4	78.4	55.8	20.3	16.9	15.1	33.2	4.6	17.5	3.3	74.2	0.7	1.3	1.8	1.8	1.8	3.2	24.6
	Jamtara	69.1	21.2	97.5	0.9	81.2	67.7	24.8	17	20.2	53.6	5	27.2	3.3	81	0.2	1.6	2.9	0.7	0.7	4	27.8
	Ranchi	81.1	51.3	89	4.1	61.6	81.6	44.9	50.9	39.8	67.5	17.9	55.8	21.7	87.9	7.6	13.9	11.7	12.9	11.4	16.6	40.3
	Khunti	67.1	24.5	72.9	1.6	74.8	73.1	16.5	22.6	19.5	30	8.2	20.1	3.8	75.5	1.1	1.1	1.7	1.2	1.4	3.2	24.9
	Gumla	71	29	64.5	3.8	76.1	75.7	12.6	27.6	21.3	34.1	9	22.3	4.4	80.4	1.3	1.2	2.8	1.4	2.3	10.7	26.8
	Simdega	79.2	28.1	86.4	2.8	76.6	73.5	17.4	21	18.4	25.7	9.1	12.8	1.8	79.1	0.7	0.3	1.9	0.8	0.6	4.9	26
	Pashchimi Singhbhum	47.2	25.7	91.2	3.4	73.7	50.4	17	17.4	16.3	32	7.8	20	5.5	65	1.1	0.6	4.3	2.7	2.1	1.9	23.2
	Saraikela Kharsawan	61.6	36.2	91.4	5.3	70.3	69.7	35.1	34.9	33.9	63.2	12.8	42.3	16.7	83.3	3.7	5.7	15.6	7.6	7.1	7	33.8
	Bargarh	64.4	48.7	90.5	1.8	84.9	74	40.5	29.9	27	69.1	5.6	50.9	10.8	80.9	0.7	3.7	15.3	2.5	2.1	3.2	33.8
	Jharsuguda	77.9	61.4	93	2.3	83.3	78.9	49.4	43.8	41.1	77.9	13.8	65.3	25.1	88.2	3.7	10.1	44.5	9.9	8	8.9	42.6
	Sambalpur	71.7	44.5	87.7	2.3	78.7	75.6	32.3	32	27.2	72.2	8.9	56.5	17.1	84.6	2.8	7.1	26	6.4	5.3	9.5	36.1
	Debagarh	71.6	43.3	86.2	3	73.1	67.8	37	24.8	17.5	49.9	3.2	35.2	8	69.9	1.3	3.8	8.6	2.4	2.2	9.9	29.9
	Sundargarh	81.2	55.5	92.1	2.9	80.9	78.8	43.4	39.7	32.9	72.9	12.9	55.7	21.5	86.2	3.5	11.3	32.8	10.2	7.5	7.7	39.9
	Kendujhar	63.6	37	73.8	8.6	67.3	64	40.7	31.3	24.9	51.3	5.3	38.5	14.7	72.3	3.2	5.5	7.2	5	4.6	8.4	30.3
	Mayurbhanj	52.1	28.5	78	5.2	79.5	57.8	26.7	21.5	19.4	46.1	4.6	30.8	8.2	70	1.4	3.3	2.8	1.7	2	6	26.3

**Odisha**	Baleshwar	75.8	40.9	76.9	5	82.5	69.8	41.6	29	27.8	80.1	7.8	54.4	15.3	86.6	2.3	7.1	3.3	3.8	5	7.5	34.8
	Bhadrak	69.8	38.9	70.2	8.4	78.1	65.3	34.6	23	18.7	75.6	7.1	51.2	10.4	85.9	1	3.4	2.6	2.5	2.3	6.4	31.5
	Kendrapara	75	43.6	75.8	5.1	73.6	71.3	33.5	29.7	24.9	87.1	8.1	58.1	13.6	88.2	1.2	5.8	2.3	1.7	2.8	4.9	33.9
	Jagatsinghapur	80.8	56.8	86	6.1	80.9	74.8	41.7	30.6	25.9	89.4	8.6	60.3	12.8	87.7	0.9	3.6	1.3	1.8	1.6	6.4	36.4
	Cuttack	81	56.7	77.3	6.5	80.9	78.7	55.8	50.9	35.4	87.6	10.7	67.8	22.8	87.7	3.7	7.2	7.2	8.1	6.6	9.2	40.5
	Jajapur	81.2	51.9	81.7	6.7	81.8	79.3	47.9	35.3	29.3	84.6	8.5	58.5	15.7	85.7	1.5	10	5.2	4.2	4.4	9.2	37.7
	Dhenkanal	82.5	51.8	85.9	1.9	77.7	73.2	53.4	36.7	30	73.4	3.2	56.2	13.8	79.2	1.6	5.4	7.8	5.9	3	11.4	36.4
	Anugul	85.3	59	88.9	1.4	82.2	79.4	51.2	40.9	33.2	72.7	6.1	56	18	81.8	2.9	6.2	12.5	7.4	3.9	14.6	39
	Nayagarh	78.1	46.4	83.8	3.3	79.2	71	45.6	37	26.4	86.1	8.7	58.5	13.7	83.7	1	5.2	3.1	2.3	1.9	9.4	35.9
	Khordha	84.2	65.4	84.3	4.3	73.5	84.1	63.4	60.6	46.4	93.6	15	74.1	31	91.3	6.1	15.6	13.7	17	14	7.2	45.3
	Puri	80.9	60.5	83.6	3.8	82.6	80.1	49.7	41.7	31.3	91.8	9.7	69.6	16.7	91.9	1.6	7.2	3.2	6.4	3.3	12.2	40
	Ganjam	78.5	48.6	66.3	2.8	66.7	79	33.7	40.9	25.8	84.2	11.3	60.3	16.2	88.3	1.1	6.6	5.2	4.6	4.8	10.1	35.5
	Gajapati	67.4	31.3	55.4	1.4	44.4	61.8	20.4	21.1	13.9	68.6	4.2	41.3	6.2	73.1	0.4	2.5	1.5	1.1	1.9	2.2	24.9
	Kandhamal	61.5	34.4	67.3	3.5	56.3	69.6	20.2	15.5	10.5	48.5	4.1	28.9	4.5	60.7	0.4	2.9	1.6	0.9	1.6	2.6	23.7
	Baudh	67.4	38.6	89.3	0.9	85.9	66.2	34	22.5	19	62.2	3.2	37.6	7.9	76.1	0.8	2.8	9.2	1.8	1.4	6.2	30.4
	Subarnapur	58.5	37.5	89	1.4	83.5	64.8	35.2	25.4	24	68.5	6.5	42.3	8	79.4	0.2	2.7	22.9	1.5	1.8	8.5	31.9
	Balangir	67.4	43.1	92.1	0.4	84.8	68	38.9	31.6	19.8	62.1	3	46.3	8.6	79.7	0.9	3.6	17.3	3	1.1	6.3	32.6
	Nuapada	65	37.5	92.9	0.6	88.8	67.2	31.4	21.3	16.8	59.2	2.4	36.7	6	79.3	1.2	3.9	10.7	2.2	1.8	3.8	30.1
	Kalahandi	58.3	31.5	84.3	1.8	76	55.2	29.2	17.4	16	46.5	4	31.4	6.2	70	0.7	2.5	6.6	2.1	2.4	1.3	25.9
	Rayagada	59	33.5	81.6	3.5	58.5	58.8	33.9	24.3	18	59.7	7	35.6	10.2	64.2	0.9	6.3	6	3.6	3.5	3.3	27.4

	Nabarangapur	45.8	22.9	52.9	3	75.1	53.7	18.7	13.3	18.8	34.6	4.8	25.1	5.8	64.5	0.5	2.9	2.6	0.8	1.5	1.9	21.5
	Koraput	45.5	26.5	52.8	1.6	53.2	52	21.4	19.4	22.1	49.1	5.6	35.1	10.4	63.2	1.7	4.9	3.8	4.4	3.3	1.3	22.8
	Malkangiri	59.2	32.5	65.2	3.2	64.8	52.4	33	15.9	19	60.2	5.9	31.8	6.7	64.5	0.4	3.8	6.1	1.7	1.1	2.5	25.3
**Chhattisgarh**	Korea (Koriya)	63	31.3	92.5	7.4	60.8	78	38.2	36.2	30.7	48.1	18.3	41.9	15.6	74.4	3.1	13.7	22.3	7.1	5.4	8	33.5
	Surguja	61.1	23.3	89.2	7	66.5	66.9	31.5	26.8	25.2	34.1	11.1	29	8	65.2	2.5	6.4	10.7	3.9	3.4	12.4	28.4
	Jashpur	63.1	23	73.8	3	65.4	70.9	23.3	30	24.5	31.5	10.9	30.7	6.4	64.2	1.9	5.4	9.3	1.3	2.4	9.4	26.7
	Raigarh	63.3	32.9	93.9	6.4	70.9	74	47.7	35.3	37.8	71.4	18.5	58.5	18.6	71.8	4.3	16.4	29.5	5	6.8	10.7	37.3
	Korba	69	34.9	96.6	3.8	67.4	82.9	53.6	46.2	40.2	67.1	26.2	63.8	25.6	79.9	4.8	16.7	41.9	10.6	8.5	11.1	41
	Janjgir - Champa	76.9	34.4	97.6	2.9	70.6	83.6	44.5	42	38.3	85.1	22.9	71.6	19.5	83.6	2.5	14.5	33.1	4.4	6.1	17.4	41.4
	Bilaspur	80.5	43.7	97.3	7.8	74.6	91.3	56.9	52.8	41.8	86.5	23.4	74.3	20.6	88.7	4.1	15.4	37.1	7.6	5.3	13	44.6
	Kabirdham	64.7	23.8	97.9	3.8	67.9	74	38.7	24.8	32.4	77.7	11.8	51.8	7.8	74.2	1.9	10.7	25.5	1.9	3	15	34.5
	Rajnandgaon	78.6	50.7	98	7.7	82.4	91.2	58.2	46.3	46.5	87	31.5	78.5	15.6	91.1	3.2	15	38.5	6.5	5.4	11.7	45.5
	Durg	80	48.7	97.2	4.9	73.5	88.4	66.3	59.2	48.5	90.1	29.3	77.6	27.2	87.9	5	20.8	54.2	12.4	9	16.7	48.3
	Raipur	71.2	39.3	95.3	3.8	67.7	83.1	57.4	51.7	42.5	84.2	23.7	72.8	23.9	84.3	4.4	19	46.6	9	7.6	16.1	43.8
	Mahasamund	66.4	30.3	97.8	2.2	81.1	79.4	36.7	28	36.9	71.2	14.3	62.2	11.6	78.8	1.9	6.9	27.5	2	2.8	14.5	36.5
	Dhamtari	77.9	40.7	95.7	4.6	82.1	86.3	46	36.3	38.7	82.5	19.7	66.4	15.3	82.4	2.7	9.7	37	4.4	4.7	14.4	41
	Uttar Bastar Kanker	78.1	40.8	94.4	4.3	74.9	75.2	48.3	37.4	33.8	68.6	17.2	54.4	15.3	74.3	3	10.6	32.4	4.4	5.6	14.3	38.2
	Bastar	56.4	24.5	70.7	2.9	74.5	62.8	21.6	21.3	26.2	49.6	8.1	35.7	8.7	66.3	2	6.1	12.4	3	3	6.6	27.1
	Narayanpur	60.8	29	84.7	8.2	78.2	80.6	27.7	21.1	24	47.2	8.7	35	8.2	64.7	0.8	3.7	13.9	1.5	1.5	2	28.7
	Dakshin Bastar	46	20.9	83.2	3.6	54.8	50.8	25.1	24.1	22.3	42.9	7.4	30.9	11.5	57.2	1.6	7.5	15.9	2.6	3.4	2.5	24.6

Bijapur	71.1	34.4	94.9	4.2	81.2	84.2	27.1	22.4	29.5	66.4	9.6	45.3	5.3	70.1	0.2	2.5	11.8	2.2	1	2	31.8
Sheopur	39.7	16.4	96.9	20.5	31.7	63.9	66.3	23.9	36.1	67.7	29.4	43.2	11.7	82.9	1.7	7.7	26.7	1.5	2.2	22.8	34.1
Morena	58.7	35.3	97.1	25.8	59.5	83.4	78.4	55.6	39.3	83.5	34.8	59.7	25.9	91	2.6	15.5	48.1	5.1	5	24.1	45.4
Bhind	58.4	30.2	98.9	29.7	60.1	77.6	81.9	44.4	41.2	78.9	28.3	55	20	92.6	2.6	13.4	39.8	4.3	3.7	17.9	42.7
Gwalior	69.7	41.8	93.8	24.1	47.3	85.1	83.4	72.3	49.6	87.8	41	73.7	45.6	91.5	5.3	28.5	69.2	15.4	11.1	32.9	52.5
Datia	56.8	32.2	97.6	30.3	47.2	79.6	67	42.4	38.9	79.4	28.1	56	21.6	88.3	1.8	15.8	38.4	5.1	3.7	23.2	41.7
Shivpuri	48.2	22.5	95	32.1	39.5	60.6	60.4	33.5	37.6	64.2	24.4	40.4	15.1	83.1	2.3	10.7	27.9	4.4	3.1	35.1	36.9
Tikamgarh	42.7	23	94.1	6	57.6	63.8	52.6	28.7	23.6	62.8	16.5	33.9	8.3	83.8	2	3.8	14.6	1.8	2.8	23	31.8
Chhatarpur	46.1	20.7	94.5	7.4	60.6	65.4	60	31.1	24	59.2	19	32.4	8.4	84.1	1.3	2.8	17.1	1.8	2.8	21.8	32.5
Panna	47	22.7	92.1	9.3	64.8	68.5	66.7	31.9	20.2	54.4	13.4	34.4	6.9	77.7	1.7	2.1	14.5	3.3	1.8	8.5	31
Sagar	49.5	28.9	81.1	11	57.7	69.1	68.6	38	26.4	56	20.4	45.1	8.7	81.5	1.8	3.9	17.3	4.4	3.4	13.7	33.3
Damoh	45.1	24.5	83.7	8.8	53.9	70.3	59	33.4	23.3	57.1	14.9	41.7	7.5	75.9	2.4	4.6	14	3.7	3.3	8.8	30.7
Satna	63.1	31.6	93.4	8.5	63.9	71.9	77.3	50.4	27.5	67.6	23.4	42.1	13.4	81.2	3.9	5.8	23.5	7.7	6.6	16.9	37.9
Rewa	59	26	95	8.5	70.1	64.2	67.8	42	23.9	61.6	17	32.8	9.1	81.4	2.1	4.3	18.9	2.6	2.7	15.6	34.3
Umaria	50.3	22.3	95.1	8.3	67.8	67.5	60.9	29.8	20	42.8	18.5	29	9.5	75.9	0.6	2.6	15.8	2.7	2.2	11.5	30.7
Neemuch	56.6	36.3	88.5	9.1	55.1	83.3	78.4	59.9	49.8	88.9	30	66.5	14.5	88.6	3.3	15.2	17.1	5.3	5.3	12.3	41.7
Mandsaur	47.5	28	85.8	8.2	57.3	72.1	77.9	48.6	43.9	85	28.4	62.5	14.2	91	3.7	15.8	14.4	5.9	4.6	23.9	40.1
Ratlam	49	28.8	80.9	7.3	42.1	71.3	67.5	51.5	40.6	70.9	27.7	54.9	18.9	82.5	2.9	12.8	17.8	11	5.7	12.9	36.7
Ujjain	64.3	39.3	91	7.8	52.6	85.3	78	62.9	52.7	87.9	31.7	71.1	23.8	92.3	5.9	14.1	27.2	13.8	9.4	15.9	44.9
Shajapur	51.7	24.9	90	17.7	58.9	84.2	79.2	58.5	46.2	90.9	25.9	62.4	14	91	1.5	12	23.5	5.7	2.9	18.6	41.8

**Madhya Pradesh**	Dewas	61.6	30.3	94.1	28.8	51	89.1	78.2	70.3	52.7	88	32.6	70.2	21.7	90.2	4.6	21.5	28.1	9.5	5.1	31.1	47.1
	Dhar	56.7	29.3	95.3	6.4	37	79.9	65.4	57.6	48.4	77.8	23.2	57.7	14.5	87.4	4.1	20.1	17.6	5.5	5	24.2	39.9
	Indore	79.4	58.1	89.3	47	57	92.1	90.2	91.6	63.6	96.4	43.4	87.4	46.8	96	10.5	35	52.1	28.6	19.9	18.3	58.1
	Khargone (West Nimar)	58.5	30.7	96.5	6.4	39.1	83.4	75	67.9	46.3	82.6	25.1	63.2	19.3	88.2	3.9	16.2	26.5	7.9	6.5	27	42.7
	Barwani	44.7	22.3	96.6	6.3	34.4	65.5	66	39	34.8	58.5	15	41.4	10.6	79	2.8	10.4	13.6	3.7	4.2	34.7	34.2
	Rajgarh	40.1	21.3	95.8	39.4	33.6	69.7	76.2	36.2	38	82.7	21.6	46.2	9.4	88.3	2.7	13.6	18.2	2.5	4.3	47.2	39.7
	Vidisha	47.7	22.3	80	9.6	41.2	73.4	61	34.4	29.1	59.6	22.8	48.6	8.4	78.1	1.6	5.1	18.1	2.5	2.6	8.4	31.6
	Bhopal	80.8	53.5	88.2	32.8	46.1	92.7	87.6	87.4	64.4	94.4	39.2	83.1	44.6	96.2	11	36.8	65.7	29.6	18.4	24.6	57.2
	Sehore	64.9	38.2	94.7	35.9	49.3	88	81.8	65.2	54.4	89.9	31	70.1	19	91.9	4.5	20.5	33.3	7.2	6.2	38	48.7
	Raisen	62.6	34.7	94.4	15.2	40.2	81	85.4	64.1	40.4	77.2	25.8	62.4	17.9	87.6	4.5	19	34.2	4.4	6.1	21.8	42.9
	Betul	60.4	41	88.5	7.8	40.9	75.8	61.9	47.4	38.2	55.6	23.9	50.4	21.5	79	3.9	5.7	30.8	9.4	4.6	20.3	37.5
	Harda	61.3	30.1	96.5	12.9	38	80.5	76.3	63.4	47.9	80.3	27.2	61.3	18.2	87.8	5.3	17.9	33.3	9.2	6.4	30.4	43.5
	Hoshangabad	68	40.6	95.5	21.5	48.6	82.8	82.7	71.4	46.2	80.6	32	64.1	22.1	89.4	4.7	23.3	44.2	8.4	7.2	26.8	47
	Katni	53.8	26	89.3	6.4	63.7	70.3	63.7	34.9	23.9	50.5	20.5	37.2	12.8	74.6	2.4	4.3	20.4	6.9	4.8	11.4	32.8
	Jabalpur	69.1	46.2	78.1	10.2	54.9	79.8	65.9	65.6	40.6	80.9	29.1	67.4	29.6	85	7.1	16.4	52.3	14.5	11.5	15.2	44.5
	Narsimhapur	58.2	29.1	87.6	7	49.7	69	66.4	43.9	33.3	58.8	16.9	47.1	12.5	75.4	5.6	6.2	24.4	5.2	6.1	16.7	35
	Dindori	33.5	8.6	78.9	11.4	33.6	53	27.8	15.2	8.6	22.1	6.9	18	2.5	63.9	1.1	2	6.3	1.2	1.6	4.1	19.3
	Mandla	50.2	19.1	79.3	8.9	42.7	58.2	25.9	28.3	17.4	38.6	11.1	33.3	8.7	68.7	1.8	4.2	17.5	2.5	3.4	7.3	25.4
	Chhindwara	66	34.8	89.8	6.6	45.2	72.2	64	43.6	34	54	21.4	52.7	15.8	81	2	4.9	27.8	4	3.7	16.6	36
	Seoni	59.5	28.8	88.5	4.8	57.7	62.5	41	31.3	23.6	47.4	14.5	38.8	9.8	74.5	1.7	4.4	21.6	3.8	4	10.2	30.4

Balaghat	71.7	39.5	96.2	8.5	73.7	70.6	55.6	30.4	27	68.6	15.2	46.7	11.1	76.3	1.9	4.8	20.8	3.3	3.6	12.4	35.7
Guna	45.9	27.2	94	16.2	42.6	76.7	73.9	48.7	43.1	77.1	27.2	58.4	12.8	88.9	3.5	14	28.8	4.5	3.9	30.8	40.4
Ashoknagar	54.6	27.5	93.1	30.8	44.6	83.5	66.6	47.3	38.5	79.5	22.1	58.8	13.5	86.3	3.1	13.7	24.5	3.4	3.4	26.2	40.3
Shahdol	50.6	25.2	93	12.5	69.2	64.9	52	27.4	19.6	41	15	29.9	6.8	67.3	2.5	3.2	15.3	3	2.5	9.9	29.5
Anuppur	58	29.7	89.4	8.6	62.9	72.5	61.7	35	30.1	46.9	20.7	40.4	13	75.8	3.6	4.3	21.9	6	4.7	6.3	33.2
Sidhi	59.3	30.1	94.3	11	60.8	63.6	54.3	29.5	16.3	42.9	14.1	26.7	4.6	75.1	2.2	2.5	12.8	1.5	2.5	10.6	29.8
Singrauli	56.5	29.4	96.1	5.8	62	71.8	60.6	32.3	25.4	39.9	15.6	29.4	12.1	75.2	3	6.4	18.4	7.3	5.9	10.4	32.1
Jhabua	32	11.6	94.5	5.3	30.4	45.2	41.6	20.4	27.5	39.3	7.8	21.4	6.6	80.1	1.6	5.6	5.4	1.8	2.6	13.1	24.1
Alirajpur	48.5	14.7	97.4	7.4	41.8	69.9	53.4	25.9	30.4	43.3	8.1	19.5	6.7	87.8	2.2	6.2	5.9	2.9	2.3	28.2	30
Khandwa (East Nimar)	51.9	30	96.3	9.3	40.4	77.4	77.8	71.5	43	74.6	23.6	57.7	15.9	84.8	2.8	14.3	24.6	7.1	4.4	24	40.7
Burhanpur	55	31.7	94	3.1	34.1	75.8	76.1	71.1	31.7	76.9	21.9	62.5	21.9	81.5	2.2	14	24.7	4.9	5.4	23.2	39.8
Kachchh	78.5	42.3	94.1	4.9	28.8	75.1	77.9	63.3	44.1	92.6	19.7	72.8	38.4	90.1	8.1	3.6	6.7	6.6	6.5	10.6	41.7
Banaskantha	56	28.5	97	3.7	21.8	67.9	86	47.5	32.9	78.4	13.4	49.7	25	91.9	5.4	1.1	3.7	2.5	3	10.8	35.1
Patan	65.4	36.7	98.9	1.4	27.4	83.4	68	60.6	32.3	91.8	14.7	63.5	28.6	92.2	7	0.9	4.6	3.5	3	6.3	37.9
Mahesana	77.4	46.6	99.1	4.4	32.9	84.3	92.9	78.4	44.7	95.2	15.6	80.4	53.4	93.7	8.8	2.8	12.5	5	8.9	11.1	45.7
Sabarkantha	70.9	37.3	99	1.7	23.7	73.3	86.4	56.9	45.5	88	8.5	56.8	34.3	89.1	7.9	1.5	8	4.2	6	13.6	39.3
Gandhinagar	73.1	52.9	98.6	2.6	34.4	83	80.4	70.7	46	91.6	10.3	76	50.7	91.4	11.3	4	13.7	5.3	9.2	13	44.3
Ahmadabad	85.9	75.4	97.9	6	30.9	93.1	90.3	92.4	68.8	98.4	23.5	86.4	69.3	95.9	15	10.2	31.3	19.3	21.9	15.7	54.4
Surendranagar	74.7	37.5	99.3	1	27.4	90.9	82.7	70.5	56.6	96.4	12.1	74.7	38.6	92.7	8.5	1.2	7.9	6.4	6	12.6	43.3
Rajkot	88.3	50.6	98.5	3.7	35.8	90.4	96.9	90.4	71.6	97.8	20.5	85	62.6	97	9.4	3.2	10.4	15.5	10.7	22.1	51.5

**Gujarat**	Jamnagar	85.6	52.4	97.2	4.2	30.5	86.5	87.6	83.4	58.4	96.7	20.2	81.5	49.7	94.3	7.2	3.6	8.5	8.8	7.3	14.5	47.3
	Porbandar	94.7	71.8	98.5	3.9	39.2	92.9	93.2	89.7	60.1	97.1	11.2	84.5	43.4	92.5	5	2.2	6	8.6	4.7	12.6	48.8
	Junagadh	91.7	47.5	99.3	0.6	37.5	94.4	84.1	83.9	58.3	99.6	11.7	83.2	41.3	96.5	7.4	4.1	5.5	6.1	6.5	16.4	47.2
	Amreli	93	74.3	98.4	1.8	40.2	95.2	92.1	91.7	66.8	98.4	14.7	83.3	50.4	94.3	7.8	1.5	5	7.6	3.7	10.3	49.6
	Bhavnagar	87.1	62.8	98.2	3.4	40.5	89.7	89.7	86.4	54	97.5	16.4	76.7	38.3	94.6	6.4	1	5.1	6.9	6.6	5.7	46.3
	Anand	81.5	46	98.1	3.2	45.2	86.9	73.1	75	43.3	93.3	12.6	72.9	38.2	91.2	6.3	3	8.7	6.9	9.6	6.5	43.2
	Kheda	70.4	35.7	97.3	4.3	37.2	73.9	67.3	59.2	35.7	90.4	10.9	62.4	26.6	84.3	5.3	2.5	4.1	3.5	6.6	6.5	37.6
	Panchmahal	76	41.9	98.9	7.1	32.4	66.5	62.6	53.7	40.7	77.5	11.5	45.7	23.5	85.4	5.8	3	5.7	5.9	5.3	10.1	36.6
	Dohad	69.5	28.4	97.6	3.5	26.3	61.9	59.2	31.9	38.6	63.4	9.6	31.9	14	85.6	4.3	1.4	3.9	5.3	3.3	5.4	31
	Vadodara	82.8	47.5	95	8.6	39.4	81.9	79.4	73.5	54.7	90.4	18.9	68.4	46.1	89.6	7	5.7	11.3	12.1	11.1	15.4	45.4
	Narmada	81.4	37.4	97.9	3.3	25.8	60.9	79.3	52.4	41.9	72.9	6.6	55	22.3	80.6	5.8	2	4.1	3.2	3.8	8	35.8
	Bharuch	84.2	57.5	96.8	3.6	27.7	85.3	83.5	78.9	42.6	92.9	15.8	73.6	48.7	89.4	6.4	4	8.1	8.1	8.5	8.6	44.4
	The Dangs	73.2	26.7	78.8	2.4	24.9	50	40.9	28.9	29.6	46.5	7.4	49.7	11.1	73.6	2.4	0.2	0.4	0.5	0.4	2.7	26.3
	Navsari	90.7	68.7	97.4	2.4	43.7	86.5	86.1	78.9	59.9	91	13.8	74.8	49.3	92.7	9.9	4.1	4.4	5.2	8.4	19.3	47.9
	Valsad	84.2	66.9	93.1	4.2	30.9	82.6	77.4	74.7	57.3	88.9	14.2	77.4	51.7	91.4	11.8	3	6.8	10.6	9.6	8	45.4
	Surat	86.2	70.3	90.8	4.9	27.9	88.4	84.2	88.5	61	97.5	26.9	80.7	58.5	96.4	11.4	5.5	11.1	15.6	11.9	8.3	49.2
	Tapi	83.2	48.7	96.8	5.6	32.5	71.3	72.9	61.1	50.5	81.3	10.5	61.1	28.3	82.8	4.5	1.4	3.5	1.8	3.1	9.1	39
**Daman and Diu**	Diu	96.4	80.1	99.3	3.3	26.3	95.1	96.2	91.2	51.7	99.4	14	88.9	73.5	93.2	4.2	3.1	9.1	11.7	9.2	10.3	50.8
	Daman	67.2	55.9	72.8	1.3	23.6	79.2	80.5	85.1	41.9	97	9.4	72.5	42.2	94.4	9.9	5.2	14	11.8	9.2	1.5	41.7
**Dadra and Nagar Haveli**	Dadra & Nagar Haveli	77.3	57.6	79.8	1.8	22.3	75.7	62.8	59.7	43.1	89.7	12.1	67.9	36	92.5	9.3	6.5	13.9	9.8	12.6	6.5	40.2

	Nandurbar	55.4	34.3	90.4	3.4	17.4	58.2	53.4	32	22	58.3	8.9	44.6	16.5	66.7	2.6	2.5	7.8	2.5	2.1	4.7	28
	Dhule	70.4	51.2	91	4.7	29.8	72.5	79.6	58.3	34.9	76.5	16.2	65	26.4	84.1	2.6	8.5	15.7	5.2	6	11.5	39.1
	Jalgaon	70.2	42.7	96.1	5.6	27.4	82	81.1	78.7	36.9	89	19.5	73	31.1	88.7	2.3	9.8	26.6	5.4	4.9	15.4	42.9
	Buldana	65	58.5	95.2	2.7	28.8	79.4	84.1	72.1	29.8	87.9	13.9	71	16.9	89.3	3.2	8.4	27	4.2	4.5	13.3	41.4
	Akola	68.6	55.3	89.3	7.7	34.7	85.4	84.5	68.9	35.6	88.5	18.9	70	26.5	88.8	2.6	12.8	48.8	8.8	6.5	13.8	44.3
	Washim	62.2	50.3	94.2	3.1	30.1	81.4	81.2	58.5	33.5	84.8	14.8	65.1	18.5	88.5	3.1	2.4	33.4	6.1	4.4	13.7	40.1
	Amravati	79.6	65.8	93.1	4	37.5	82	82.2	71.4	41	83.1	20.1	75.1	31.8	83.7	4.5	20.6	51.4	10.8	8.5	7.3	45.7
	Wardha	86.1	61.3	95.8	6	37.3	83.5	90.9	73	38.5	88.4	17.1	76.7	27.3	89.1	2.8	10.2	64	5.6	4.3	15.2	47
	Nagpur	88.2	68.4	92.1	13.5	48.4	89.8	89.9	84.8	53.8	93.7	29.7	83.2	47.9	92.7	7.8	25	70.6	17.3	14.9	14.5	54.3
	Bhandara	86.3	75	98.3	2.8	70.4	90.1	87.4	67.9	41	92.4	19.2	71.1	25.3	90.1	2.2	10	44.1	2.7	5.2	10.3	47.7
	Gondiya	84.5	63.8	98.4	3.4	74.5	84	79.7	59.4	32.3	87.5	13.2	60.5	13.3	85.2	2.2	9.9	29.3	2.9	2.8	6.9	42.9
	Gadchiroli	77.6	56.2	96.3	2.5	56.1	81.6	76.4	40.1	25.9	82.7	10	59.3	13.1	78.7	1.3	1.7	23.9	1.6	2.8	5.1	38
	Chandrapur	82.3	58.9	97.8	2.9	45.9	76.3	79	59.6	39.8	86.1	18.7	65.9	32	84.3	4.7	11.3	42.7	8	6.6	12.3	44.2
	Yavatmal	68.4	54.6	92.7	6.4	29.4	78.9	83.7	58	29.6	82.6	12.4	66.6	24	87.5	3.9	3	32.6	5.1	4.7	9.3	40.1
	Nanded	66	47.7	92.7	5.7	21.1	80.5	75.3	52.8	28.1	83.8	19.7	58.7	18.8	87.9	1.9	8.6	22.8	3.3	3.6	11.3	38.2
	Hingoli	57.4	45.1	94.4	4.9	23.7	77.3	72.5	60.8	31.6	82.5	11.1	56.7	17.5	85.7	2.4	9.5	21.1	3.5	4.3	15.4	37.7
	Parbhani	60.9	52.8	91.8	4.6	25.3	82.7	83.3	60.3	31.8	87.8	14.3	59.3	18.2	93.2	1.6	7.4	27.7	2.6	2.8	14	39.8
**Maharashtra**	Jalna	53.8	39.9	86.7	2.4	23.3	74	69	64.6	37.2	80.6	11.1	58.2	13.9	85.7	1.7	4.6	14.3	2.1	2.7	15.3	36
	Aurangabad	71.3	59.4	91.1	8.1	29	85.5	88.2	78.9	52.9	87.8	19.7	78	27.4	92.5	4.5	14.3	26.1	6.1	8	12	45.4
	Nashik	75.4	55.3	84	7.5	31.5	84.6	85.6	78.3	49.9	87.2	20.3	74.2	37.5	90.4	9	21.7	14.1	10.1	13.1	10.3	45.3

	Thane	80.6	72.3	79.8	8.2	21.8	91.3	81.9	78.8	36.4	93.9	21.3	83.9	57.2	95.3	8.3	23.6	18.1	29.8	20.1	2.6	48
	Mumbai Suburban	68	60.5	69.5	6.4	14.9	89.9	82.8	84.3	18.9	97.7	18.8	84.1	49.1	97.5	3.4	12.9	12.7	17.6	12.6	3.1	43.2
	Mumbai	68.5	62.4	65	8	13.4	91.3	78.9	84.2	29.5	98.4	17.5	84.7	63.9	97.3	4.8	23.2	24	34.1	23.9	1.1	46.4
	Raigarh	88.5	76	86.6	7.6	30.5	91.6	88.2	79.3	45.5	95.6	22.1	84.9	59.8	93.2	11.7	22	21.9	22.3	21.7	2	50.1
	Pune	75.7	65.3	81.4	14.2	28.1	90	84.2	85.7	60.1	92.3	20.3	85.6	57.1	96	13.6	35.8	23.7	18.1	24.1	5.9	50.6
	Ahmadnagar	80.9	68.9	89.1	14.5	45.3	80.8	85	73.7	53.6	82.6	25.1	67.7	24.9	85.6	5	15	20.9	4.7	6.7	17.9	46
	Bid	66.1	53.2	83	3.6	26.9	76.9	74.9	57.4	31.7	82.5	17.1	59.5	17.1	89.6	1.3	2.8	16.5	1.6	3.1	11.2	37.5
	Latur	66.8	41.7	87.5	2.4	31.5	71.6	64.7	55.2	30.9	75.8	17.9	58.3	15.3	91	0.9	7.2	15.6	1.6	2.6	11.8	36.3
	Osmanabad	62.2	42.1	78.1	5.2	38.8	73.2	64.6	61.3	34.1	73.6	15.7	60.1	16.5	87	1.5	7.3	11.2	0.4	2.3	18.5	36.8
	Solapur	73	52.8	81.1	6.1	42.3	85.7	76.8	62.5	48.9	83.8	17.9	71.1	27.2	92.4	4.7	15.9	13.3	4.7	5.7	11.1	42.3
	Satara	74.4	53	80.8	15.4	43.8	82.8	76.1	72.3	47.9	79.3	23.2	74.1	29.8	89.5	6.9	15.1	8.3	7.6	8.1	15.1	43.7
	Ratnagiri	87.7	68.4	72.1	6.8	23.2	90.1	67.3	53.1	26.2	90.3	13.8	62.2	29	87.8	6.5	7.1	9.8	10.3	6.8	9.2	39.8
	Sindhudurg	95.2	82.2	80.7	6.8	28.1	91	80.2	60.5	33.8	90.4	20.9	69.3	27.2	87.2	5.5	5.3	6.4	9.4	6.6	11.7	43.3
	Kolhapur	87	63.7	78.5	18	49.3	87.4	73.3	70	52.9	77.3	28.7	76	27	94.3	6.9	6.6	3.7	8.4	9.8	11.3	44.8
	Sangli	82.8	65.5	81.5	16	52.7	85.3	80	73.7	51.2	80.8	31.2	74.5	26.4	92.8	7.1	9.6	10.8	6.3	9.4	12.9	45.9
	Adilabad	80.6	48.5	92	1.7	25.7	65.8	57.1	24.8	28.7	86.2	12.3	66.3	14.9	84.1	1.9	2	19.8	3.8	3.2	23.8	36.5
	Nizamabad	84.3	41.3	69.6	2.4	30.8	66.8	52.6	31.4	34	89.9	11.6	71.4	18.2	90.8	2.2	1.2	13.7	4.1	3.1	22.3	36.4
	Karimnagar	88.3	55.7	92	3.4	36.3	72.2	65.1	30.4	34.8	93.6	20.5	76.4	20.7	87.3	3.1	2	15.7	6.6	4.3	31.2	41.4
	Medak	81.5	37.2	54.3	2.2	34.1	68.6	45.3	33.3	31.4	90.5	14.2	71.1	14.8	89	3.2	5.3	6.8	5	5.8	35.6	36.4
**Telangana**	Hyderabad	94.2	70.9	81.3	5.6	21.3	89.1	77.8	75.6	58.2	98.4	19.3	91.4	53.6	98.2	13.8	16.5	39.3	32	18.5	46.2	54.6

**Telangana**	Rangareddy	91.3	64	82.5	4.7	20.4	81.7	74.2	63.2	52.1	95.9	18.9	88	47.7	95.2	8.2	12.7	34.9	21.9	15.2	31.4	49.3
	Mahbubnagar	79.1	37.7	76.2	1.4	16.3	64.7	40.4	28.7	27.6	86.4	14	66.2	12.8	89.7	1.9	1.4	8	3.4	2.7	19.7	33.2
	Nalgonda	87	46.3	89.8	1.6	30.9	61.9	49.3	32.8	37.2	93.8	14.1	73.7	16.9	89.5	2.5	1.7	18.7	5.3	3.7	34.3	39.3
	Warangal	83.9	55.4	88.5	4.3	32.2	63.1	45.4	31	28.6	90.7	12	73.5	14.5	84.5	2.9	3.4	14.9	6.1	4.2	39.2	38.9
	Khammam	91.9	69.5	94.3	0.8	31	72.9	59.6	41.6	32.3	93.7	14.1	78.3	22.6	86.3	2	2.4	25.2	7.8	3.1	39.9	43.3
**Andhra Pradesh**	Srikakulam	92	60.2	85.6	1.3	33.8	78.4	62	41	23.4	94.9	8.2	74.4	16.8	86.5	1	2.1	7.1	3.2	2.2	10	37.8
	Vizianagaram	90	73	95.6	0.9	34.4	73.6	65.1	41.1	23.9	94	8.2	77.5	18.9	85	2	2.7	6.2	5.2	3.7	15.2	39.6
	Visakhapatnam	92.2	62.7	91.9	3.3	29.8	80.2	67.6	51.5	36.7	91.2	14.5	80	34.4	86.5	4.8	8.2	15.1	14.7	9.2	30.6	44.6
	East Godavari	94	61.2	94.5	2.3	61.5	85.1	74.5	43.3	40.1	96.5	18.1	82.6	29.1	90.1	2.9	6.3	11.2	9.2	6.6	31.3	46.3
	West Godavari	95.2	51	93.8	2.4	49.6	73.6	70	39.1	34.7	94.9	17.8	80.4	23.4	85.8	1.7	3.4	11.8	5.4	4.3	39.8	43.7
	Krishna	96	67.8	94.4	2.2	49.9	79.5	65	44.7	36.8	97.1	13.7	82.1	27	90.6	2.5	3.6	17.7	8.6	5.7	29.9	45
	Guntur	92.2	68.2	97.8	1.7	41.2	71.3	54.5	41.8	31.7	96	13.4	79.9	21.4	86.7	1.4	2.5	16.7	6	2.6	36.3	42.8
	Prakasam	89.7	61.5	94.7	1.4	35.1	68.2	60.1	44.4	34.2	91.8	11	78.1	18.4	86	1.2	1.8	13.2	4.5	1.8	21.9	40
	Sri Potti Sriramulu N	90.9	56	94.5	2.5	29.9	77.1	68.2	43.7	30.1	94.2	13.6	85.2	27.7	88.8	0.5	5.2	11.8	10.5	3.7	24.4	42
	Y.S.R.	91.7	65.1	94.9	2.8	23.8	77.6	77.8	58.2	36.4	95.1	13.3	85.1	32.4	91.1	1.5	2.2	24.4	13.6	4.5	24	44.7
	Kurnool	84.1	67.8	87.5	0.7	40.5	77.4	60.4	50.9	36.5	96.3	16.8	84.6	20.7	92.3	1.4	2.1	13.7	4.3	2.5	19	41.8
	Anantapur	89.1	52	88.4	1.5	30.9	82.8	64.4	42.9	35.5	95	22.2	83.1	19.9	91.4	0.8	3.9	9.1	3.3	2.7	18.4	40.7
	Chittoor	88.1	56.5	81.5	2.7	28.3	75.1	72.9	47.8	38.3	88.9	11.4	81.7	26.8	87.8	2.1	3.6	10.3	8.3	4.9	25.6	41.3
	Belgaum	87.3	58.4	84.4	4.5	55.6	92.7	92.4	68.9	49.3	79.9	28.5	77.5	14.2	92.6	3.2	2.9	2.6	4.6	4.3	6	43.6
	Bagalkot	80.2	58.1	73.3	3.2	41.9	81.4	87	44.4	36.2	73.2	18.5	71.2	8.2	91.3	1.5	3	1.2	1.5	2	4.7	37.5

**Karnataka**	Bijapur	76.6	52.9	72.4	4.7	30.3	76.5	84.9	42.1	31.8	65.8	21.3	69.2	11.7	87.6	3	3.5	2.5	3.1	3	9	36.2
	Bidar	83.1	61.8	81.1	3.4	35.8	85.4	87	52.8	34.6	79.4	22.1	69.9	10.8	90.1	1.1	2.2	5.1	5.2	3.2	4.7	39.2
	Raichur	74.2	55	77.6	2.6	30.8	82.6	89.7	34	38.1	81.2	20.6	71.3	13.4	85.8	2.2	3.2	4.6	4.5	3.6	5.3	37.4
	Koppal	78.1	54.5	69.9	4.4	43.3	85.9	95	43.2	41.5	78.5	20.7	77.8	7.4	89.3	1.3	3.1	0.7	2	1.5	5.3	38.5
	Gadag	75.4	47.5	71.2	6.1	31.9	74.4	77.4	37.3	30	69	18.3	70.6	9.4	81	3	4.3	1.3	4	3.2	5.4	34.6
	Dharwad	83.8	68.8	85.6	6.5	32.8	91.5	88.3	62.2	43.7	77.2	23.7	81.2	26.6	94.4	7.2	7.4	4.6	9.9	10.7	19.1	45
	Uttara Kannada	94.6	72.2	81	7.4	42.2	88.1	93.6	56.1	39.4	73.1	24.7	77.3	22	90.5	5.5	7.5	2.4	9	5.9	18.4	44.3
	Haveri	80.3	59	71	5.4	40.6	88.7	94.2	52.8	36.3	73.3	20	77.3	7.1	88.5	1.8	3.9	0.8	2	2.3	7.4	39.1
	Bellary	80.6	62.7	77.7	4.5	35.2	84.2	90.8	54.6	39.8	84.6	22	80.2	13.8	91.1	1.9	3.3	3.8	4.1	3.7	4.9	40.4
	Chitradurga	80.3	55.1	73.7	6.4	31.6	83.1	84.1	51.1	38.7	73.2	17.5	74.9	13.6	86.7	3.2	5.7	1.3	4.9	4	7.1	38.3
	Davanagere	88.2	68.7	79.7	4.1	40.8	89.8	83.6	63.6	42.6	87.6	18.9	85.8	16.2	92.4	4.1	6.3	2.4	7.8	7.2	9.2	43.2
	Shimoga	92	72.2	82.6	11.8	42	86.6	95.6	67.6	45.8	68.1	24.6	83.7	21.1	90.2	9.8	10.6	4.1	13.3	7.7	16.5	45.8
	Udupi	93.3	75.2	87.2	11.9	34.4	94.8	89.2	58.5	45.5	91.4	19.2	86.8	42.8	96.3	12.3	11.9	4.9	16.5	11.5	50.6	51.7
	Chikmagalur	88.9	66.7	83.8	8.5	32.5	84.7	89	64.9	37.5	57.5	14	82.5	13.5	91.2	6.9	7.8	1.6	8.5	4.5	11.6	41.3
	Tumkur	82.4	63.1	85.3	4	35	87.6	89.7	57.7	38.3	74.3	12.7	77.8	12	88.1	3.5	2.9	0.5	5.7	2.9	9.8	40.1
	Bangalore	94.8	86.2	93.6	11	32.7	94.3	92.2	94.1	67	96.7	19.9	93.5	60.2	97.5	17.5	34	14.8	41.4	28.2	13.8	57
	Mandya	87.8	69.2	84.2	9.5	48.4	87.9	92.5	70.9	49.4	74.2	15	83.7	13.2	91.2	2.8	5.5	2.2	4.4	3.7	6.2	43.2
	Hassan	86	69.7	92.2	10	30.2	93	94.8	72.4	42.3	60	16.9	82.4	16.2	91.8	7.3	3.9	1.1	8.7	3.7	19.4	43.9
	Dakshina Kannada	95	82.4	82.8	20.3	26.6	90.1	85.2	62	40.1	90.5	26.9	83.9	41.5	95.2	14.5	17.9	7.2	20	15.6	28.3	50.2
	Kodagu	92.6	85.4	91.3	26.3	20.6	90.8	95.5	74.5	31.3	57.9	21.5	82.7	24.4	92	16.1	10.9	2.4	11.9	5.9	14.3	45.8

	Mysore	85.4	63.3	80.8	12.9	39.7	87.7	79.9	71.8	44.3	67.9	20.8	80.7	21.3	88.4	5.7	12.7	2.4	12.2	8.4	8.1	43
	Chamarajanagar	74.2	52.4	74	5.3	45.7	83	86.6	45.1	27.5	49.6	10.6	72	3.4	83.1	2	1.9	0.6	3.1	1.8	4.9	34.8
	Gulbarga	77.9	58	75.2	4.6	38.9	78.7	83.3	45	33.2	76.3	17.9	79.3	12.9	91.1	3.5	3.6	4.4	5.5	3	2.2	37.9
	Yadgir	72.7	43.7	72.1	2.2	28.8	76.3	90.4	25.2	30.3	70.2	15.4	62.2	8.2	84.8	2	4.2	3.4	2.6	2.6	6.3	33.8
	Kolar	84.4	66.1	80.6	5.6	38.7	90.5	88.9	72	42.1	80.1	16.1	81.5	20	90.9	3.3	6	2.4	9.3	6.5	6.8	42.8
	Chikkaballapura	80.2	69.9	82.7	6.4	29	87.2	94.8	67.1	33.9	70.1	14	74.6	15.9	90.3	3.9	6.3	1.9	6.3	5.5	9.8	40.9
	Bangalore Rural	85.4	68.3	88.5	7.9	38.4	89.1	93.7	76.1	46.3	80.4	18.5	82.7	26.1	90.8	6.2	4.9	1.1	11.1	5.7	10.4	44.8
	Ramanagara	89.2	68.6	83.6	10.6	37.3	91.7	95.4	75.6	41.5	79.1	17	84.3	19.3	89.5	3	6.2	2.4	8.2	5.4	10.6	44.2
**Goa**	North Goa	95.9	88.6	83.8	11.7	28.7	98.2	95.8	87.6	72.3	97.7	39.5	91.8	65.6	97.6	30.3	19.4	20.1	49.2	22.6	7.9	57.7
	South Goa	95.3	83.8	89.3	12.5	23.2	98.5	91	86.5	76.5	98.5	35.4	94.5	75	97.1	28.7	16.1	17.8	50.1	22.3	4.1	57.2
**Lakshadweep**	Lakshadweep	99.2	98.4	99.1	16.9	82.8	95.6	94.5	86.4	67.3	99.7	43.1	73.5	83.5	98.7	2.6	3	12.7	41	12.7	85.8	65.8
**Kerala**	Kasaragod	98.3	93.4	95.1	13.5	22.1	93.8	92.1	81.6	44.3	95.3	33.3	88.8	66.6	98	18.2	7.8	8.2	27	14.7	70.2	58.7
	Kannur	99.2	97.4	98.4	21	19.5	96.9	96.6	88.1	45	97.3	36.1	90.9	77.2	97.1	21.3	9.6	10.9	33.9	17.1	73.4	61.9
	Wayanad	96.2	87.4	91.9	14.8	19.6	89.7	86.6	71.4	37.4	66.7	26.3	84.7	38.5	96.3	12.7	6.1	1.4	15.8	12.6	48.3	50.1
	Kozhikode	99.6	97.2	98.9	23.9	33.1	96	96.6	90.2	61.8	97.7	40.2	91.6	73.9	98.4	24.4	13.8	11.8	36.6	23.9	72.9	64.5
	Malappuram	99.7	96.2	98.5	15.4	26.2	91.2	94.5	89.5	57.3	97.7	39.7	87.2	70.9	98.5	25.7	10.9	9.9	35.1	19.2	76.5	62.7
	Palakkad	98.9	87.7	94.7	9.6	24.4	94.4	88.1	78.6	60.8	95.9	26.5	92	60.6	97.6	22.7	13.5	8.3	31.2	21.8	51.5	57.6
	Thrissur	99	95.8	98.6	24.4	33.7	97	96	90.6	59.8	99.2	43.4	94.9	78.2	98.1	24.8	18.2	15.2	42.8	27.7	73.4	65.9
	Ernakulam	99.7	98.2	99.6	26	29.1	97.5	96.5	95	67.5	99.4	41.3	96.2	82.1	98.7	38.6	24.7	21.4	51.9	37.7	62.9	67.9
	Idukki	97.5	90.5	96.9	15.8	25.7	93.2	87.4	76.4	48.8	83.5	32.7	94.7	54.7	96.7	17.3	9.5	3.6	26.1	18	51.2	55.8

Kottayam	100	99.1	99.7	16.3	17.7	95.9	95.3	91.1	55.4	98.9	38.6	93.7	78.9	97.9	28.6	15.1	12.3	35.8	25.4	59.9	62.7
Alappuzha	98.8	90	98.3	25.4	46.3	95.7	89.7	83.7	54.3	98.3	32.6	93.5	68.7	96.4	17.9	12.8	7.9	27.3	21.8	57.3	60.7
Pathanamthitta	99.8	94.1	99.5	22.2	23.6	93.2	92.6	82.5	53.3	97	35.9	93.1	75.3	97.4	24.7	15.4	8.2	32.5	22.2	64.5	61.5
Kollam	98.6	93.1	96.5	12.9	27.6	90.6	90.8	81.1	56.7	95.3	39.9	92.8	75	96.2	18.1	13.2	7.1	25.6	23.1	66	60.3
Thiruvananthapuram	98.4	92	97.4	25.5	22.8	95	81.8	69.7	54.7	96.8	32.4	90.4	70	96.1	23.7	16.5	12.6	24.8	26.6	58.3	59.2
Thiruvallur	93.5	66.2	73.8	9	50.1	93.8	63.4	81.2	64.9	98.2	21	96.2	57.6	95.9	6.4	13.3	19.8	31.5	21.2	9.6	51.3
Chennai	96.6	66.7	83.6	12.3	51.3	96	73.8	94.6	74.5	99.5	27.8	97.5	75.4	98.3	14.6	23.9	30.4	60.2	32.9	19.2	59.4
Kancheepuram	91.2	62.3	77.1	15.9	53.9	89.7	64.4	82.1	66.7	98.6	20	97.6	66.1	96	12.3	20.2	25.4	38	26.1	22	54.7
Vellore	88.8	63.7	73.5	5.9	68.5	93.1	59.9	74.7	60	96.6	18.1	94.6	46.8	92.5	3.1	5.6	7.7	18.4	13.7	8.2	47.7
Tiruvannamalai	78	43.4	60.5	5.4	63.7	82.7	41.9	52.9	53.5	94.9	11.3	93.9	19.1	89.2	1.6	3.5	3.8	6	6	7.2	39.3
Viluppuram	82	45	64.5	6.8	57.7	89.2	38.5	48.5	54.8	97	12.2	94.3	28.8	93	1.3	3.7	3.7	8.6	9.7	2.3	40.2
Salem	89.4	56.3	81.7	9	60.5	90.4	53.8	68.5	63.5	95.2	18	95.5	33	92.7	3.6	5.4	5.3	12.8	14.4	2.8	45.4
Namakkal	93.3	52.3	84.1	14.8	61.3	85.6	49.4	83	63.9	96.8	24.2	96.5	33.4	92.8	3.1	5.1	4.4	11.7	10.2	7	46.7
Erode	93.6	53.5	84.1	12.1	59.2	88.8	53	86.5	67.6	95.2	15.5	95.9	32.7	93.3	3.7	5.5	2.2	11	11	4.9	46.4
The Nilgiris	93.1	72.6	88.8	6	22.8	90.2	78.4	80.1	32.5	37.7	15.7	97.1	9.6	93.1	5.1	3.6	0.5	13.6	10.1	2.2	40.7
Dindigul	90.9	41.9	66	7.1	55.7	89.8	45.4	70.8	48.3	90.7	11.1	93.3	21.6	94.4	1.9	2.6	1.8	11.7	7.2	3.9	40.9
Karur	94.5	59.8	85.1	10.6	57.9	89.3	57.8	77.2	65.1	97.5	19.4	95.9	32.6	92	5.9	5.4	5.8	13.1	10.6	2	46.6
Tiruchirappalli	88.4	59	75.3	20.9	52.2	88.4	45.8	69.4	59.5	96.2	17.1	92.4	38.1	91.4	6.3	9.9	9.8	21	18.3	14.4	47.1
Perambalur	85.4	48.9	74	6	51.3	88.2	44.9	54.9	52.2	96.7	15	93.1	30.8	91.6	2.4	4.3	4.9	9.9	10.9	2.5	41.4
Ariyalur	82.1	42.5	64.7	9.1	58	84.8	33.1	44.7	45.1	94.6	10.6	92.2	15.9	90.7	1.5	1.8	3	4.3	5.1	11	38.4

**Tamil Nadu**	Cuddalore	84.4	47.2	59.5	14.5	61.5	88.2	38.2	47.3	52.1	96.2	16.8	92.8	33.3	92.5	3.9	5.2	6	9.8	11.5	9.5	41.9
	Nagapattinam	89.3	42.3	60.3	15.6	57.4	83.1	35.9	48.8	43.3	95.1	12	92.4	28.9	90.7	2.9	5	3.2	9.2	8.6	23.7	41.5
	Thiruvarur	92.2	51	56.5	18.2	69.3	89.4	36	43.7	47.6	94.9	16.1	92.3	27	92	1.8	4.1	3.8	7.8	9.8	16.8	42.2
	Thanjavur	89.9	53.8	62	12.4	63	86.5	41.3	49.9	52.4	94	16.1	90.1	28.9	89.3	3.2	4.9	6.3	11.6	11.5	13.8	42.6
	Pudukkottai	86.6	46	68.8	10.7	60.4	85.5	40.4	37	57.5	93	11	92.1	25.4	92.7	2.3	4.2	4	7.9	9.9	6	40.3
	Sivaganga	89.9	43.4	60.9	18.7	53	91.8	43.6	54.7	52	95.6	22.7	91.5	39.3	92.9	4.7	3.6	5.3	12.5	11.5	8.6	43.1
	Madurai	82.1	44.6	74	22	46.4	84.9	42.3	72.8	46.3	97.5	23.5	94.2	38.1	92	4.5	8.7	8	20.7	15.7	16.8	45.3
	Theni	85.5	46.6	75.5	10.7	46.8	88.1	45.5	69.7	41.3	94.4	19.7	92.3	20.8	88.6	2.9	4.4	5	14.9	7.2	12.4	42.1
	Virudhunagar	77.5	41.4	69.9	13.3	51.8	85.7	36.5	62.1	40	96.8	16.7	91.2	24	86.4	3.2	4.7	5.9	11.4	6.4	13.1	40.5
	Ramanathapuram	83.5	40.9	65.1	12.9	42.6	81.8	38.9	42.9	37.6	93.4	15.4	90.4	31.5	91.8	2.4	4.5	3.7	11.2	8.2	5.8	38.6
	Thoothukkudi	84.3	54.5	76.2	17.7	54.2	80.2	42	61.1	46	94.7	20.1	90.6	27.5	88.4	3.7	5.1	4.4	13.8	11.9	13.4	43
	Tirunelveli	83.6	45	72.2	14.2	51.8	87.5	39.1	50.4	36.5	94.9	16.1	89.6	21.2	89.4	3.3	3.8	5.9	11	10.1	15	40.7
	Kanniyakumari	98.4	80.9	87	15.9	32.7	93.1	65.4	60.7	53.1	97.6	29.3	94.1	59	95.9	7.8	9.2	4.1	17.2	18.2	12.1	49.7
	Dharmapuri	87.6	55.9	77.1	6.7	63.6	89.6	53.6	68.9	60.7	95.5	17.6	94.7	25.7	92	1.9	5.6	3.4	6.7	8.3	6.8	44.2
	Krishnagiri	89.6	49.8	68.6	9.1	56.8	90.5	56.1	67	61.4	96.6	15.8	96.2	34.3	96.3	4.3	7.2	4.5	13.6	13.3	5.6	44.9
	Coimbatore	93.9	60.5	74.6	13	50.6	91	57.3	82.2	63.3	95.1	16.8	97.1	39.1	96.5	4.5	6.5	4.5	24.1	13.2	5.9	47.4
	Tiruppur	94.1	67.4	71.8	7.6	56.5	90.8	51.3	86.2	68.2	96.9	16	96.5	29.2	94	1.2	3.3	3.9	14.6	7.4	3.6	45.9
	Yanam	97.1	75	87.6	3.7	65.4	91.8	85.3	64.4	46.2	98.2	21.6	90.4	51.3	95.7	3.6	6.9	13.9	19.5	8.7	21.8	50.9
**Puducherry**	Puducherry	90.8	57.4	68.8	10.9	60.5	92.9	59	78.2	72	98.9	19.6	95.5	66.3	96.1	9.5	13.9	12.9	33	17.7	7.2	50.9
	Mahe	99.6	97.3	96.4	16.4	34.1	94.5	94.6	93.9	53.8	98.3	29.7	96.7	75.4	96.5	18.6	12.4	17.8	45	17.9	13.8	57.9

	Karaikal	92.9	63.5	70.7	17.6	58.6	90.5	59	67.2	62.9	97.1	24	94.5	56.1	92.2	7.4	9.9	17.4	27.1	16	10.8	49.8
**Andaman and Nicobar**	Nicobars	81.7	85.6	57.1	51.8	61.6	92.4	35.8	77.8	32.9	94.4	58.1	75.8	34.9	80	5.3	2.4	0.1	11	1.9	0.8	44.9
	North & Middle	92.1	78	78.4	5	19.2	90.2	47.4	58.2	23	86.8	16.6	80.4	49.4	89.9	5.5	11.3	0.1	12.6	6	11.3	41.6
	South Andaman	92.6	90.3	92.3	11.6	12.5	93.3	73.1	84.7	46.7	97.3	21.2	93.2	70.7	97.1	13	20.5	4.1	36.9	17.7	5.7	51.4
